# Structure—yeast α-glucosidase inhibitory activity relationship of 9-*O*-berberrubine carboxylates

**DOI:** 10.1038/s41598-023-45116-0

**Published:** 2023-11-01

**Authors:** Duy Vu Nguyen, Kowit Hengphasatporn, Ade Danova, Aphinya Suroengrit, Siwaporn Boonyasuppayakorn, Ryo Fujiki, Yasuteru Shigeta, Thanyada Rungrotmongkol, Warinthorn Chavasiri

**Affiliations:** 1 Department of Chemistry, Faculty of Science, Center of Excellence in Natural Products Chemistry, Chulalongkorn University, Pathumwan, Bangkok, 10330 Thailand; 2 Center for Computational Sciences, University of Tsukuba, 1-1-1 Tennodai, Tsukuba, Ibaraki 305-8577 Japan; 3 Organic Chemistry Division, Department of Chemistry, Faculty of Mathematics and Natural Sciences, Institut Teknologi Bandung, Bandung, West Java 40132 Indonesia; 4 Department of Microbiology, Faculty of Medicine, Center of Excellence in Applied Medical Virology, Chulalongkorn University, Bangkok, 10330 Thailand; 5 Bioinformatics and Computational Biology Program, Graduated School, Chulalongkorn University, Bangkok, 10330 Thailand; 6 Department of Biochemistry, Faculty of Science, Center of Excellence in Biocatalyst and Sustainable Biotechnology, Chulalongkorn University, Bangkok, 10330 Thailand

**Keywords:** Drug discovery, Chemistry

## Abstract

Thirty-five 9-*O*-berberrubine carboxylate derivatives were synthesized and evaluated for yeast α-glucosidase inhibitory activity. All compounds demonstrated better inhibitory activities than the parent compounds berberine (**BBR**) and berberrubine (**BBRB**), and a positive control, acarbose. The structure–activity correlation study indicated that most of the substituents on the benzoate moiety such as methoxy, hydroxy, methylenedioxy, benzyloxy, halogen, trifluoromethyl, nitro and alkyl can contribute to the activities except multi-methoxy, fluoro and cyano. In addition, replacing benzoate with naphthoate, cinnamate, piperate or diphenylacetate also led to an increase in inhibitory activities except with phenyl acetate. **9**, **26**, **27**, **28** and **33** exhibited the most potent α-glucosidase inhibitory activities with the IC_50_ values in the range of 1.61–2.67 μM. Kinetic study revealed that **9**, **26**, **28** and **33** interacted with the enzyme via competitive mode. These four compounds were also proved to be not cytotoxic at their IC_50_ values. The competitive inhibition mechanism of these four compounds against yeast α-glucosidase was investigated using molecular docking and molecular dynamics simulations. The binding free energy calculations suggest that **26** exhibited the strongest binding affinity, and its binding stability is supported by hydrophobic interactions with D68, F157, F158 and F177. Therefore, **9**, **26**, **28** and **33** would be promising candidates for further studies of antidiabetic activity.

## Introduction

Diabetes mellitus is a chronic metabolic disorder characterized by uncontrolled hyperglycemia due to inadequate production or ineffective use of insulin in the body which can result in many consequences such as neuropathy, nephropathy, stroke and cardiovascular disease^1^. In 2021, there are approximately 537 million adults diagnosed to have diabetes in the world and the number is predicted to reach 783 million by 2045^2^. In addition, 6.7 million death due to diabetes was recorded and at least 966 billion dollars were expensed for treatment of diabetes^2^. Type 2 diabetes, the repercussion of excess body weight and physical inactivity, accounts for more than 95% of the people living with diabetes. This type of diabetes was diagnosed only in adults but now it tends to appear frequently in children^3^. A potential therapeutic approach for diabetes mellitus, particularly in type 2 diabetes, is to decline postprandial hyperglycemia by using α-glucosidase inhibitors to prevent carbohydrate digestion. α-Glucosidase located in the brush-border surface membrane of intestinal cells, is an essential hydrolytic enzyme in the carbohydrates digestion process, it degrades oligosaccharide to monosaccharide^4^. The production of these absorbable glucose results in postprandial hyperglycemia in patients with diabetes. Some α-glucosidase inhibitors such as acarbose, miglitol and voglibose, which are carbohydrate mimetics, have been used in the clinic for the treatment of type 2 diabetes. Nevertheless, because of their side effects including flatulence, diarrhea, stomach ache and liver damage, it is necessary to develop new, efficient and benign α-glucosidase inhibitors for the treatment of diabetes mellitus^5^. Recently, there have been several research about α-glucosidase inhibitors^6–9^.

Berberine (**BBR**), a quaternary ammonium salt from the protoberberine group of isoquinoline plant alkaloids, is used for medicinal purposes in 3000 years-long histories of the Indian and Chinese. Berberine was found as an active component in the root, rhizome and stem bark of many well-known medicinal plants such as *Hydrastis canadensis* (goldenseal), *Coptis chinensis* (Coptis or goldenthread), *Berberis aquifolium* (Oregongrape), *Berberis vulgaris* (barberry) and *Berberis aristata* (Treeturmeric)^10^. Berberine possesses a wide range of biological activities such as antimicrobial^11^, anti-Alzheimer^12^, antidiabetic^13^, antihypertensive^14^, anticancer^15^, and anti-inflammatory activity^16^. Nevertheless, efficient applications of berberine are hindered by its poor bioavailability which is less than 5%^17,18^. It is attributed to its poor aqueous solubility (~ 1.8 mg/mL at 20 °C) since berberine has a temperature-dependent aqueous solubility which increases with an increase in temperature^19^. In addition, berberine is a hydrophilic compound with a log *p* value of − 1.5 which makes berberine lipophobic with limited membrane permeability, and this leads to low gastrointestinal absorption^20^. Therefore, to improve the bioavailability of berberine, its new derivatives have to be designed and synthesized with their enhanced aqueous solubility and permeability through intestinal membranes.

**Figure 1:**
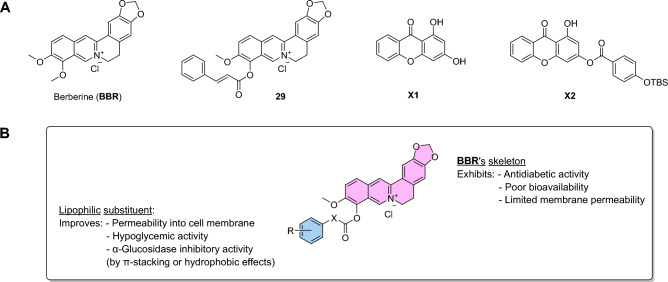
(**A**) Structures of berberine, 1,3-dihydroxyxanthone and their derivatives. (**B**) intended modification of **BBR** to enhance its α-glucosidase inhibitory activity.

There have been several reports about the antidiabetic activity of berberine. It was proved that the antihyperglycemic activity of berberine in the Caco-2 cell line is partly based on its α-glucosidase inhibitory activity^21,22^. Maltase and sucrase activities were also suppressed by berberine^23^. Additionally, treatment of berberine could considerably attenuate the activities of intestinal disaccharidases in STZ-induced diabetic rats and normal rats^24,25^. In addition, to enhance the antidiabetic activity of berberine, the introduction of lipophilic substituents has been proved to be a potent strategy since it can improve the pharmacological activities and bioavailability of berberine^12,26–28^. A series of 9-*O*-berberrubine carboxylates possessing the 9-*O*-lipophilic group were reported to show low cytotoxicity and good hypoglycemic activity against HepG2 cells^29^. Compound **29** demonstrated the most substantial increase in hypoglycemic activity with glucose consumption (GC) of 6.73 mM compared with berberine (GC = 5.04 mM) while its cytotoxicity was lower than berberine (Fig. 1). On the other hand, it was reported that yeast α-glucosidase inhibitory activity of xanthone derivatives was considerably improved by adding an aromatic ring to their structure^30,31^. 3-Arylacyloxyxanthone (**X2**) was one of the outstanding candidates with the IC_50_ value of 10.6 μM which was superior to 1,3-dihydroxyxanthone (**X1**) (IC_50_ = 145 μM)^32^. It was claimed that the π-stacking and hydrophobic effects of the additional benzene rings to the α-glucosidase enzyme were the key factors for enhancing inhibitory activity. Therefore, 9-*O*-berberrubine carboxylate derivatives were designed with the 9-substitution of lipophilic moiety for evaluation of yeast α-glucosidase inhibitory activity (Fig. 1). In this study, baker’s yeast α-glucosidase was used to evaluate inhibitory activity of compounds. Further studies involving rat, human intestinal enzymes and cell-based experiments need to be performed to confirm their antidiabetic activity^33,34^.

## Results and discussion

### Synthesis

**Figure 2:**
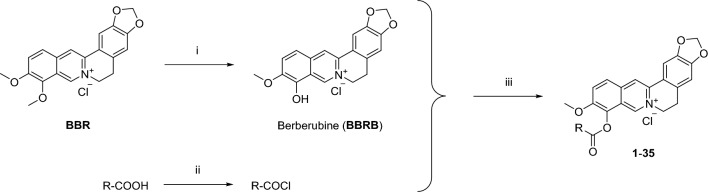
Synthetic routes of **BBRB** and **1**–**35**. Reagents and conditions: (i) 190^o^ C, 2 h; (ii) PPh_3_, Cl_3_CCN, CH_2_Cl_2_, rt, 2–3 h; (iii) 4-Picoline, CH_2_Cl_2_, reflux, 6–8 h.

The preparation of **BBRB** and 9-*O*-berberrubine carboxylates (**1**–**35**) is shown in Fig. 2. **BBRB** could be efficiently synthesized in the yield of 90% by selective demethylation of **BBR** chloride at the 9-OCH_3_ group. Berberine was heated at 190 °C for 1–2 h^29^. Various carboxylic acids such as benzoic acid, naphthoic acid, cinnamic acid, phenylacetic acid and piperic acid were used to form an ester with berberrubine. Acid chloride derivatives were prepared in situ at room temperature in CH_2_Cl_2_ solvent from the corresponding carboxylic acid using the reagents such as triphenylphosphine and trichloroacetonitrile. Then, **BBRB** and 4-picoline were added to the mixture, and the reaction was refluxed for 8 h to obtain 9-*O*-berberrubine ester derivatives (**1**–**35**) in low-to-moderate yield^35,36^. ^1^H-, ^13^C-NMR and HR-MS spectra of compounds can be seen in the Supplementary Information.

#### α-Glucosidase inhibitory activity

The inhibitory activity of berberrubine derivatives toward yeast α-glucosidase was evaluated using a protocol similar to those described in the literature^37^. The percentage inhibition at 50 μM and IC_50_ values of **1**–**35**, together with **BBR**, **BBRB** and acarbose, for comparison are displayed in Tables 1 and 2.

**Table 1:** α-Glucosidase inhibitory activity of 9-*O*-berberrubine benzoate derivatives.


Compd	R	Inhibition at 50 μM (%)	IC_50_ (μM)	Compd	R	Inhibition at 50 μM (%)	IC_50_ (μM)
**BBR**	-	NI	–	**14**	3-F	62.98	32.84 ± 1.88
**BBRB**	-	NI	–	**15**	4-F	73.86	21.29 ± 1.97
**1**	H	57.67	25.32 ± 2.63	**16**	2-Br	95.32	6.45 ± 0.52
**2**	2-OCH_3_	79.08	19.52 ± 1.62	**17**	2-I	94.91	9.24 ± 0.50
**3**	3-OCH_3_	89.67	11.62 ± 1.19	**18**	2-CF_3_	86.10	15.73 ± 1.34
**4**	4-OCH_3_	80.03	18.44 ± 1.31	**19**	2-NO_2_	81.61	10.54 ± 1.03
**5**	3-OH	98.11	7.51 ± 0.42	**20**	4-CN	55.62	26.53 ± 2.50
**6**	3,4-diOCH_3_	73.89	19.12 ± 1.01	**21**	2,6-diCl	96.64	5.86 ± 0.35
**7**	3,4-OCH_2_O	99.72	6.44 ± 0.50	**22**	2-CH_3_	90.60	18.12 ± 0.68
**8**	3,4,5-triOCH_3_	71.95	23.87 ± 1.87	**23**	4-CH_3_	78.92	17.50 ± 1.11
**9**	2-OCH_2_Ph	100.30	2.29 ± 0.08	**24**	4-C_2_H_5_	81.04	12.52 ± 0.99
**10**	2-Cl	88.45	12.21 ± 0.82	**25**	4-C(CH_3_)_3_	98.87	3.53 ± 0.31
**11**	3-Cl	95.47	9.43 ± 0.80	**26**	3,5-diC(CH_3_)_3_	99.84	1.61 ± 0.08
**12**	4-Cl	96.50	10.58 ± 0.60	Acarbose	–	–	93.60 ± 0.50
**13**	2-F	71.98	24.89 ± 1.78

**Table 2:** α-Glucosidase inhibitory activity of other 9-*O*-berberrubine carboxylate derivatives.


Compd	R	Inhibition at 50 μM (%)	IC_50_ (μM)
**27**		99.75	2.67 ± 0.27
**28**		95.72	2.63 ± 0.23
**29**		88.46	15.15 ± 0.60
**30**		91.87	10.34 ± 0.84
**31**		98.89	4.64 ± 0.55
**32**		94.81	15.96 ± 0.67
**33**		98.70	1.95 ± 0.06
**34**		93.86	7.30 ± 0.51
**35**		95.23	6.19 ± 0.32
Acarbose	–	–	93.60 ± 0.50

A series of 9-*O*-berberrubine benzoate derivatives with a wide range of substituents on the benzoate moiety were synthesized and assessed for their α-glucosidase inhibitory activities, the structures and biological results of twenty-six compounds (**1**–**26**) are presented in Table 1. 9-*O*-Berberrubine benzoate (**1**) showed better % inhibition than **BBR** and **BBRB**, i.e., **1** demonstrated 58% inhibition while **BBR** and **BBRB** showed no inhibitory activity at 50 μM, which indicated that the introduction of a benzene ester could boost the inhibitory activity as expected. Moreover, **1**–**35** displayed IC_50_ values in the range of 1.61–32.84 μM which were higher inhibitory activities than acarbose (IC_50_ 93.60 μM)—a marketed drug for type 2 diabetes. The effect of substituents such as electron-donating, electron-withdrawing and alkyl groups on the additional phenyl ring was then evaluated.

**2**–**4** exhibited higher inhibition with the IC_50_ values of 19.52, 11.62 and 18.44 μM, respectively compared with **1** (25.32 μM), indicating that the inhibitory activity increased when the methoxy group was substituted to the benzoate aromatic ring. In addition, the methoxy group at the 3-position could improve the activity better than those at the 2- or 4-position. **5** bearing 3-OH group possessed the IC_50_ value of 7.51 μM which was more active than **3** bearing the 3-OMe group. It could be rationalized by taking into account the hydrogen bonding or other electrostatic interaction with the enzyme^31^. Hydroxy group is known as an H-bonding donor/acceptor; therefore, it can efficiently form hydrogen-bonding interaction with α-glucosidase enzyme while the methoxy group only acts as an H-bonding acceptor^38^. Some studies on α-glucosidase inhibitors also proved the importance of the hydroxy group in enhancing inhibitory activity^39,40^. For multi-methoxy substituent, **6** bearing 3,4-diOCH_3_ and **8** bearing 3,4,5-triOCH_3_ groups displayed comparable or even lower activity than mono-methoxy substituent (**2**–**4**) with the IC_50_ values of 19.12 and 23.87 μM, respectively, indicating that addition of more methoxy groups was not a proper way to strengthen the activity. Surprisingly, inhibitory activity was significantly amplified by inserting the 3,4-methylenedioxy group into the phenyl moiety. **7** demonstrated a potent IC_50_ value of 6.44 μM, which might result from the compactness of the methylenedioxy group compared to the dimethoxy group. Another aromatic ring was added by replacing the 2-methoxy group with the 2-benzyloxy group of benzoate moiety, leading to dramatic enhancement of α-glucosidase inhibitory activity of **9** (IC_50_ = 2.29 μM) compared with **2** (IC_50_ = 19.52 μM), which re-emphasized the crucial role of phenyl moiety for the inhibitory activity.

Halogen and other electron-withdrawing groups were also found to have a considerable contribution to the activity. **10**–**12** bearing chloro substituent at 2-, 3- and 4-position, respectively possessed the IC_50_ values of 12.21, 9.43 and 10.58 μM, which were better than **1**. Because of the potential of chloro group for the activity, other halogen groups such as fluoro, bromo and iodo together with trifluoromethyl, nitro and cyano were introduced. It is worth noting that the fluoro substituent at 2-, 3- or 4-position of **13**–**15** completely abolished the activity with the IC_50_ values of 24.89, 32.84 and 21.29 μM, respectively. Nevertheless, **16** and **17** possessing 2-Br and 2-I groups showed better inhibitory activity with the IC_50_ values of 6.45 and 9.24 μM compared with **10** possessing 2-Cl, especially **16**. It has been reported that the chemical softness is an important factor in alleviating α-glucosidase inhibitory activity; thus, chloro, bromo and iodo substituents tend to enhance the activity better than fluoro substituent^31,41^. On the other hand, **18** and **19** bearing strong EWGs such as 2-CF_3_ and 2-NO_2_ groups exhibited the IC_50_ values of 15.73 and 10.54 μM, which were comparable to **10**. Interestingly, the activity of **20** bearing the 4-CN group was diminished with the IC_50_ value of 26.53 μM. **21** with 2,6-diCl substituents showed significantly high inhibitory activity compared with **10** with the IC_50_ value of 5.86 μM, indicating the potential of multi-halogen benzoate moiety for α-glucosidase inhibitory activity.

**22** and **23** possessing 2- and 4-CH_3_ groups displayed higher activities than **1**, their IC_50_ values are 18.12 and 17.50 μM, respectively. Replacing the 4-CH_3_ group with the 4-C_2_H_5_ group rendered the amelioration of the inhibitory activity in **24** with the IC_50_ value of 12.52 μM. The considerably higher IC_50_ value of 3.53 μM was obtained by **25** possessing 4-C(CH_3_)_3_ substituent, implying that the hydrophobic effect from branched alkyl groups is essential for α-glucosidase inhibitory activity. In addition to π-stacking and hydrophobic interaction from aromatic rings which can increase the activity^30–32^, other structural moieties such as the nonconjugated π-system can also be used to modulate the inhibitory activity by hydrophobic interaction^42^. To prove the hypothesis, **26** bearing two lipophilic *tert*-butyl groups at 3,5-positions were evaluated for the activity. **26** exhibited even higher inhibitory activity than **25** with the IC_50_ value of 1.61 μM.

**Figure 3:**
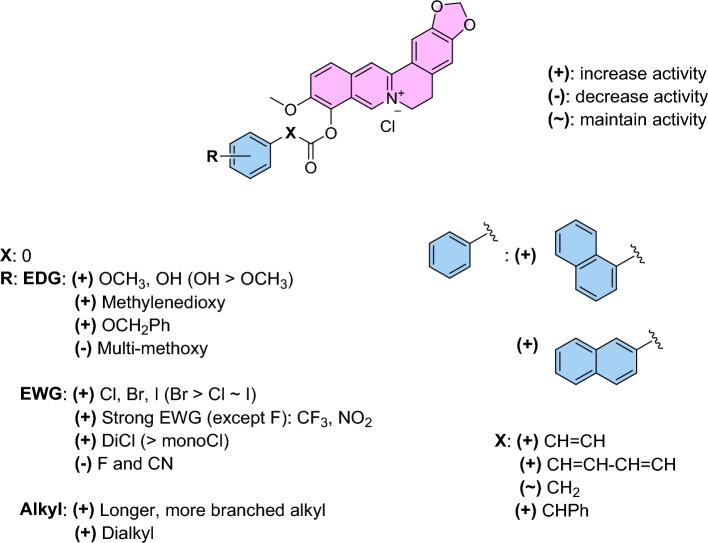
SAR analysis of 9-*O*-berberrubine carboxylate derivatives.

Besides, other kinds of 9-*O*-berberrubine carboxylates (**27**–**35**) were utilized to evaluate their structure-relationship correlation and their biological results are displayed in Table 2. 9-*O*-Berberrubine 1-naphthoate (**27**) and 9-*O*-berberrubine 2-naphthoate (**28**) demonstrated dramatically high activities with the IC_50_ values of 2.67 and 2.63 μM. Their inhibitory activities were much higher than **1** due to the π-stacking and hydrophobic effect between extended π-system of the naphthalene ring and α-glucosidase enzyme^30,38,42^. In addition, their identical IC_50_ values indicated that there is no preference between linearly and angularly fused aromatic rings to elicit the activity. 9-*O*-Berberrubine cinnamate (**29**) and 9-*O*-berberrubine α-methyl cinnamate (**30**) exhibited higher activities than **1** with the IC_50_ values of 15.15 and 10.34 μM. The increase in their inhibitory activities results from the π-stacking and hydrophobic effect of π-conjugated systems of the styryl moiety. The methyl group at α-position of **30** seemed to amplify the hydrophobic effect leading to the higher activity of **30** compared with **29**. 9-*O*-Berberrubine 3,4-methylenedioxycinnamate (**31**) also showed higher inhibitory activity than **7** with the IC_50_ of 4.64 μM. 9-*O*-Berberrubine piperate (**33**) bearing one more conjugated double bond than **31** displayed even better activity than **31** with the IC_50_ value of 1.95 μM, which confirmed that π-stacking and hydrophobic effect of long π-conjugated systems involving aromatic rings and double bonds could be utilized to modulate α-glucosidase inhibitory activity. However, there was one exception that 9-*O*-berberrubine 2,6-dichlorocinnamate (**32**) showed lower activity than 9-*O*-berberrubine 2,6-dichlorobenzoate (**21**), the IC_50_ value of **32** was 15.96 μM. 9-*O*-Berberrubine 2,6-dichlorophenylacetate (**34**) exhibited comparable inhibitory activity to **21** with the IC_50_ value of 7.30 μM indicating that the conjugation between carbonyl and phenyl groups was not important for the activity. 9-*O*-Berberrubine diphenylacetate (**35**) displayed substantially higher inhibitory activity than 9-*O*-berberrubine benzoate (**1**) with the IC_50_ value of 6.19 μM, which re-emphasized that inserting a benzene ring can strengthen the activity. From the analysis of the structure–activity relationship which is also summarized in Fig. 3, five compounds **9**, **26**, **27**, **28** and **33** exhibited the strongest inhibitory activities with the IC_50_ values of 2.29, 1.61, 2.67, 2.63 and 1.95 μM, respectively. Four compounds (**9**, **26**, **28** and **33**) were selected for kinetic study because of the resemblance of structures and activities between **27** and **28**. Due to several differences in size and amino acid sequences among yeast, rat and human intestinal α-glucosidase enzymes, additional research relating to rat, human intestinal enzymes and cell-based experiments need to be carried out to corroborate the antidiabetic activity of the potent compounds.^43^.

### Kinetic study

**Figure 4:**
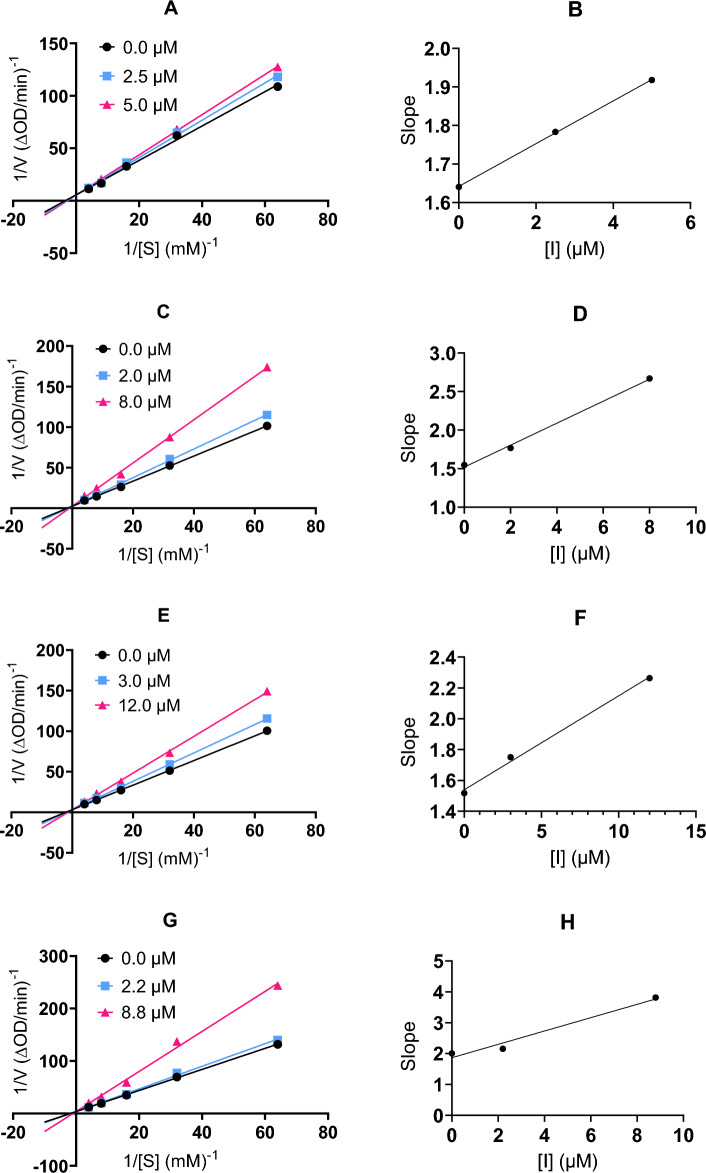
Lineweaver − Burk plots for α-glucosidase inhibition by **9** (**A**), **26** (**C**), **28** (**E**) and **33** (**G**). Plots of slopes versus concentration of **9** (**B**), **26** (**D**), **28** (**F**) and **33** (**H**) for the determination of the inhibition constant *K*_i._

To further investigate how these 9-*O*-berberrubine carboxylates interact with yeast α-glucosidase, the inhibition types of four potential compounds, **9**, **26**, **28** and **33** were studied by using Lineweaver–Burk plot analysis. As shown in Fig. 4, the double reciprocal plots showed straight lines with the same *V*_max_, indicating that **9**, **26**, **28** and **33** are competitive inhibitors of α-glucosidase. The inhibition constants (*K*_i_) were 29.64 μM for **9**, 10.67 μM for **26**, 25.27 μM for **28** and 8.64 μM for **33**.

### Cytotoxicity

**Figure 5:**
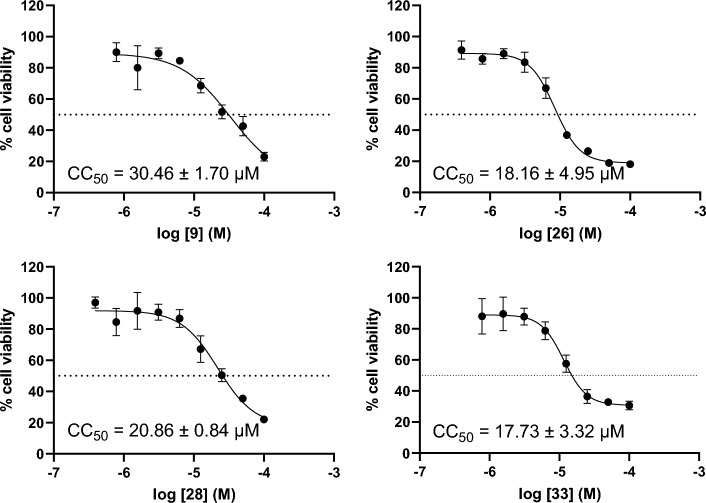
The cytotoxicity of HEK-293 cells when incubated with the compounds **9**, **26**, **28** and **33**, respectively.

To determine the toxicities of the 9-*O*-berberrubine carboxylate derivatives, the most four potent compounds were tested with HEK-293 cells. The results from three independent experiments showed that **9**, **26**, **28** and **33** displayed CC_50_ values of 30.46, 18.16, 20.86 and 17.73 μM, respectively (Fig. 5), indicating that these compounds are not cytotoxic at their IC_50_ values in the range of 2.63–1.61 μM.

### Molecular mechanism of potent compounds

**Figure 6:**
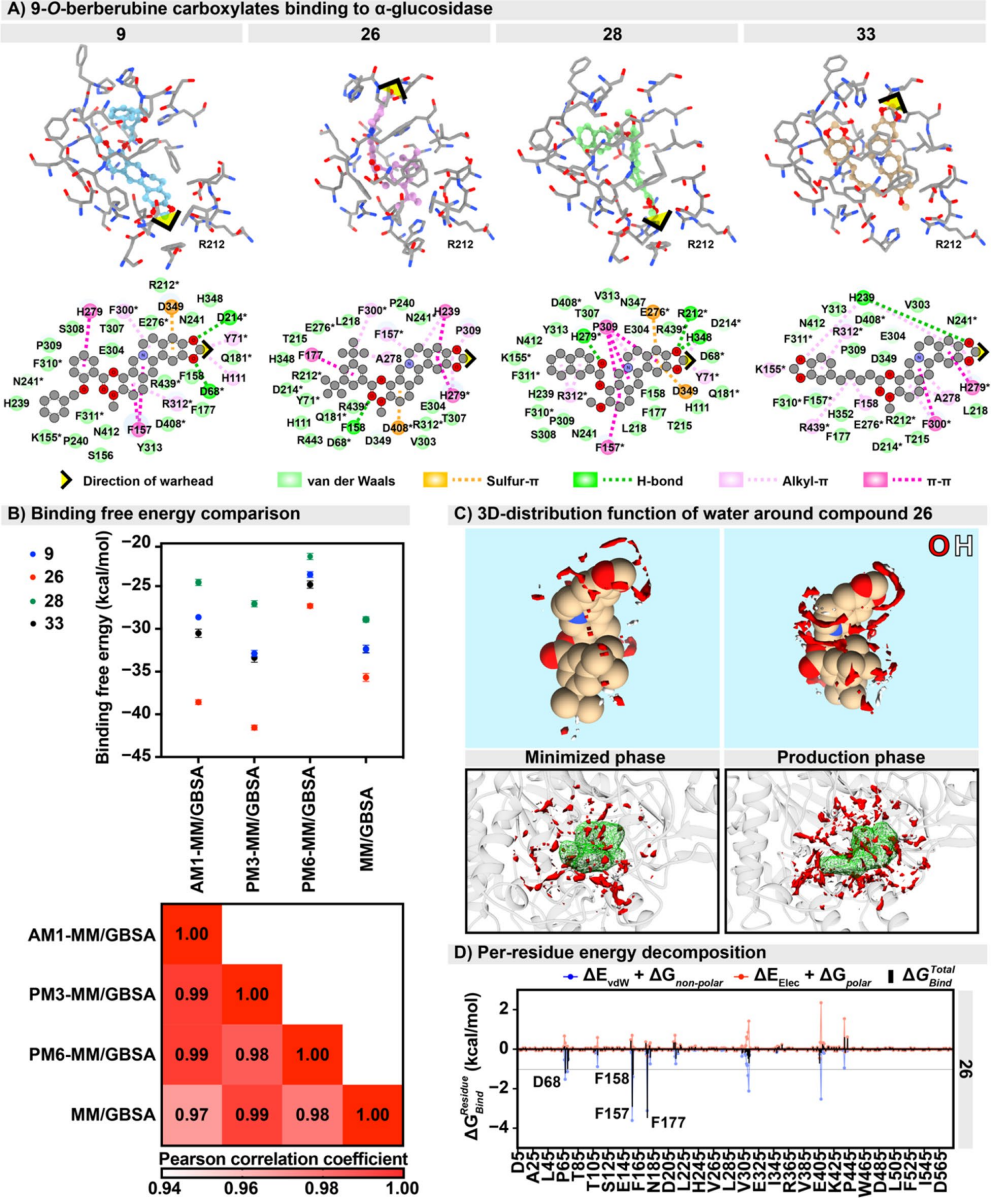
(**A**) Conformation and interaction of four potent 9-*O*-berberrubine carboxylates **9**, **26**, **28**, and **33** binding to yeast α-glucosidase at the active site resulted from molecular docking study. Black arrow and R212 are used as a reference point for this picture. The residue with asterisk refered to the interacted residue was found in the acarbose/α-glucosidase complex. (**B**) MM/GBSA and QM-GBSA binding free energies and Pearson correlation coefficient for the four simulated complexes using the 200–300 ns MD trajectories. (**C**) Water distribution function around **26** via 3D-RISM calculation with g(r) > 5.0 on the minimized structure and the last MD snapshot. The red and white color represent water oxygen and hydrogen distributions. (**D**) MM/GBSA per-residue energy decomposition for **26/**α-glucosidase complex. Key binding residues with binding free energy ≤ − 1.0 kcal/mol are labelled.

To understand the competitive inhibition mechanism at the atomistic level suggested by the kinetic study, **9**, **26**, **28**, and **33** were docked to the active site of yeast α-glucosidase using the Autodock Vina 1.2.3 program. In this study, the 3D structure of α-glucosidase in the molecular docking study was performed using the same amino sequence as we used in the experimental study, which is a different sequence compared to the available structure in the protein databank (PDB ID: 3A4A). These sequences show 84.9% similarity based on needle pairwise alignment as shown in the Supplementary Information, resulting in a slightly different residue number. Figure 6A shows that the binding pattern for these four compounds is somewhat diverse. The warhead of **9** and **28** inserts to the inner pocket, while that of **26** and **33** points out of the binding pocket. The van der Waals (vdW), π–π, S-π, and alkyl-π interactions are significantly contributed for 9-*O*-berberrubine carboxylates binding in particular at the core structure. Their theoretical binding affinities are likely comparable, i.e., − 10.40, − 10.87, − 10.46, and − 11.39 kcal/mol for **9**, **26**, **28**, and **33**, respectively. By comparing to previous studies^44,45^, the binding interaction energies of the native inhibitor (acarbose) and the glyceollin were of − 8.70 and − 10.30 kcal/mol, where the electrostatic interaction was identified as a major contribution in the acarbose/α-glucosidase complex^46,47^. Our compounds shared same interacting residues with acarbose (residues with an asterisk in Fig. 6A). The results suggested that our compounds are potential to be the novel yeast α-glucosidase inhibitor.

To elucidate the structural dynamics of 9-*O*-berberrubine carboxylates/α-glucosidase complexes, all-atom molecular dynamics (MD) simulation was performed for 300 ns. A total of 100 snapshots extracted from the last 100 ns was analyzed in terms of binding strength based on MM/GBSA and QM-MM/GBSA with AM1, PM3, and PM6 methods treated on the compound molecule only. The plot of binding free energies predicted from all calculations in Fig. 6B reveals that **26** shows the greatest binding affinity among the four studied compounds. Additionally, Pearson correlation coefficient among the calculated binding free energies for these potent compounds shows that our prediction based on four different methods is highly correlated to each other (r^2^ ≥ 0.97). Importance of water molecules in complexation was then studied by 3D-RISM solvation free energy calculation. The surrounding waters around **26** before and after MD simulation were compared in Fig. 6C. The 3D-RISM solvation free energy difference of ligand between minimized (249.19 kcal/mol) and production phases (232.84 kcal/mol) is about − 16 kcal/mol^48,49^. The rearrangement of **26** upon simulation increased the solvent molecules as the bridge of interaction between interacted residues and ligand. The MM/GBSA per-residue decomposition free energy analysis on **26**/α-glucosidase complex in Fig. 6D revealed that D68, F157, F158 and F177 in the active site were important for the binding of **26**.

## Conclusion

In conclusion, a series of 9-*O*-berberrubine carboxylates have been synthesized and evaluated as yeast α-glucosidase inhibitors. All compounds showed higher inhibitory activities compared with **BBR**, **BBRB** and acarbose. The analysis of structure–activity relationship revealed that hydrogen bonding, π-stacking, hydrophobic effect and chemical softness are essential factors modulating the activity. **9**, **26**, **27**, **28** and **33** displayed the strongest activities with the IC_50_ values in the range of 2.67–1.61 μM. **9**, **26**, **28** and **33** were found to inhibit yeast α-glucosidase via a competitive mechanism. Additionally, cytotoxicity results verified the safety of these four compounds at their IC_50_ values because their CC_50_ values were higher than 15 μM. The theoretical binding affinities of these four compounds (− 10.40 to − 11.39 kcal/mol) were higher than those of acarbose and glyceollin (− 8.47 and − 10.30 kcal/mol). These ligands showed a high binding stability over the 300-ns MD simulations. Among the outstanding compounds, **26** was the most susceptible compound toward yeast α-glucosidase. Overall, these potent 9-*O*-berberrubine carboxylates could be utilized for further studies of antidiabetic activity.

## Methods

### General information

The reagents (chemicals) were purchased from TCI and used without further purification. α-Glucosidase from *Saccharomyces cerevisiae* (EC.3.2.1.2.0) and p-NPG (p-nitrophenyl-α-D-glucopyranoside), and acarbose (positive control) were purchased from Sigma Aldrich. Nuclear magnetic resonance (NMR) spectroscopy was performed on JEOL JNM-ECZ500R/S1 spectrometer at 500 MHz (500 MHz for ^1^H NMR, 125 MHz for ^13^C NMR). Chemical shifts were reported in parts per million (ppm, d) referenced to the residual solvent signals (DMSO-*d*_6_: *δ*_H_ = 2.50, *δ*_C_ = 39.5 ppm; Methanol-*d*_4_: *δ*_H_ = 3.31, *δ*_C_ = 49.0 ppm). Proton coupling patterns were described as singlet (s), doublet (d), triplet (t), quartet (q), multiplet (m), and broad (br). HRESIMS were determined on a micrOTOF-Q II 10,335. All reactions were monitored by thin-layer chromatography (TLC) on silica gel G1P4S6. Anhydrous solvents were purchased from commercial suppliers.

### General procedure for pyrolysis of berberine

The pyrolysis of berberine (**BBR**) (3.0 g, 8.068 mmol) was performed at 190 °C for 1–2 h to produce berberrubine (**BBRB**).

*9-hydroxy-10-methoxy-5,6-dihydro-[1,3]dioxolo[4,5-g]isoquinolino[3,2-a]isoquinolin-7-ium chloride (****BBRB****):* Dark red powder (2.598 g, 90%); ^1^H NMR (500 MHz, CD_3_OD): δ ppm 9.24 (1H, s), 8.00 (1H, s), 7.50 (d, *J* = 8.5 Hz, 1H), 7.39 (s, 1H), 6.89 (d, *J* = 8.0 Hz, 1H), 6.81 (s, 1H), 6.01 (s, 2H), 4.59 (t,* J* = 6.0 Hz, 2H), 3.85 (s, 3H), 3.10 (t, *J* = 6.5 Hz, 2H); ^13^C NMR (125 MHz, CD_3_OD): 162.8, 150.9, 150.7, 149.5, 147.2, 135.8, 133.7, 130.7, 124.1, 122.8, 121.4, 119.8, 109.2, 105.8, 103.3, 98.8, 56.7, 55.7, 28.9.

### General procedure for synthesizing compounds 1–35

Trichloroacetonitrile (0.3 mL, 3 mmol) was added to a mixture of carboxylic acid (1.5 mmol) and triphenylphosphine (0.787 g, 3 mmol) in CH_2_Cl_2_ (20 mL) at room temperature. The mixture was stirred for 1–2 h. After that, berberrubine (0.358 g, 1 mmol) and 4-picoline (0.292 mL, 3 mmol) were added to the above mixture and the reaction mixture was refluxed for 8 h. Then, the organic layer was extracted with 1 M HCl and saturated NaHCO_3_, dried over anhydrous MgSO_4_, and evaporated in vacuo. The residue was purified by silica gel column chromatography (chloroform/methanol) to afford the products (**1**–**35**).

*9-(benzoyloxy)-10-methoxy-5,6-dihydro-[1,3]dioxolo[4,5-g]isoquinolino[3,2-a]isoquinolin-7-ium chloride (****1****):* Yellow powder (0.168 g, 36%); ^1^H NMR (500 MHz, DMSO-d_6_): *δ* ppm 9.92 (1H, s), 9.02 (1H, s), 8.26 (1H, d, *J* = 9.5 Hz), 8.21 (2H, m), 8.20 (1H, d,* J* = 8.0 Hz), 7.78 (1H, t,* J* = 7.5 Hz), 7.76 (1H, d, *J* = 5.0 Hz), 7.63 (1H, s), 7.62 (1H, t,* J* = 8.0 Hz), 7.01 (1H, s), 6.10 (2H, s), 4.84 (2H, t,* J* = 6.5 Hz), 3.95 (3H, s), 3.12 (2H, t, *J* = 6.0 Hz); ^13^C NMR (125 MHz, DMSO-d_6_): 163.5, 150.5, 150.0, 147.8, 144.6, 138.2, 134.7, 133.6, 133.0, 130.9, 130.5, 129.2, 128.0, 127.0, 125.9, 121.3, 120.7, 120.4, 108.5, 105.6, 102.2, 57.3, 55.3, 26.2.

*10-methoxy-9-((2-methoxybenzoyl)oxy)-5,6-dihydro-[1,3]dioxolo[4,5-g]isoquinolino[3,2-a]isoquinolin-7-ium chloride (****2****):* Yellow powder (0.023 g, 5%); ^1^H NMR (500 MHz, DMSO-d_6_): δ ppm 9.85 (1H, s), 9.05 (1H, s), 8.32 (1H, d, *J* = 9.0 Hz), 8.25 (1H, d,* J* = 9.0 Hz), 8.18 (1H, dd,* J* = 8.0, 2.0 Hz), 7.82 (1H, s), 7.75 (1H, td, *J* = 7.8, 1.5 Hz), 7.32 (1H, d, *J* = 8.5 Hz), 7.18 (1H, t,* J* = 8.0 Hz), 7.08 (1H, s), 6.17 (2H, s), 4.92 (2H, t,* J* = 6.5 Hz), 4.03 (3H, s), 3.92 (3H, s), 3.20 (2H, t, *J* = 6.5 Hz); ^13^C NMR (125 MHz, DMSO-d_6_): 161.8, 160.3, 150.8, 150.1, 147.8, 144.6, 138.2, 135.8, 133.9, 132.8, 132.7, 131.1, 126.8, 126.0, 121.2, 120.7, 120.5, 120.4, 116.8, 113.1, 108.6, 105.6, 102.2, 57.4, 56.2, 55.6, 26.3; HRMS (ESI) calcd for C_27_H_22_ClNO_6_ [M–Cl]^+^ 456.14416, found 456.1442.

*10-methoxy-9-((3-methoxybenzoyl)oxy)-5,6-dihydro-[1,3]dioxolo[4,5-g]isoquinolino[3,2-a]isoquinolin-7-ium chloride (****3****):* Yellow powder (0.148 g, 30%); ^1^H NMR (500 MHz, DMSO-d_6_): δ ppm 9.98 (1H, s), 9.08 (1H, s), 8.34 (1H, d, *J* = 9.0 Hz), 8.28 (1H, d,* J* = 9.5 Hz), 7.86 (1H, d,* J* = 8.0 Hz), 7.82 (1H, s), 7.73 (1H, t, *J* = 2.5 Hz), 7.62 (1H, t, *J* = 8.0 Hz), 7.42 (1H, dd,* J* = 8.5, 3.0 Hz), 7.09 (1H, s), 6.18 (2H, s), 4.91 (2H, t,* J* = 5.5 Hz), 4.03 (3H, s), 3.89 (3H, s), 3.20 (2H, t, *J* = 6.5 Hz); ^13^C NMR (125 MHz, DMSO-d_6_): 163.6, 159.8, 150.7, 150.3, 148.0, 144.7, 138.4, 133.8, 133.3, 131.2, 130.7, 129.5, 127.4, 126.1, 123.0, 121.5, 120.9, 120.6, 115.3, 108.7, 105.8, 102.4, 57.6, 55.9, 55.6, 26.4.

*10-methoxy-9-((4-methoxybenzoyl)oxy)-5,6-dihydro-[1,3]dioxolo[4,5-g]isoquinolino[3,2-a]isoquinolin-7-ium chloride (****4****):* Yellow powder (0.338 g, 69%); ^1^H NMR (500 MHz, DMSO-d_6_): δ ppm 9.92 (1H, s), 9.04 (1H, s), 8.29 (1H, d, *J* = 9.5 Hz), 8.24 (1H, d,* J* = 9.0 Hz), 8.20 (2H, d,* J* = 8.5 Hz), 7.78 (1H, s), 7.20 (2H, d, *J* = 8.5 Hz), 7.06 (1H, s), 6.15 (2H, s), 4.90 (2H, t,* J* = 6.0 Hz), 4.00 (3H, s), 3.91 (3H, s), 3.18 (2H, t, *J* = 6.5 Hz); ^13^C NMR (125 MHz, DMSO-d_6_): 164.4, 163.2, 150.6, 150.1, 147.8, 144.6, 138.2, 133.9, 133.1, 132.9, 131.0, 127.0, 125.9, 121.5, 120.8, 120.4, 120.1, 114.6, 108.5, 105.7, 102.3, 57.4, 56.0, 55.4, 26.3.

*9-((3-hydroxybenzoyl)oxy)-10-methoxy-5,6-dihydro-[1,3]dioxolo[4,5-g]isoquinolino[3,2-a]isoquinolin-7-ium chloride (****5****):* Yellow powder (0.168 g, 35%); ^1^H NMR (500 MHz, DMSO-d_6_): δ ppm 10.29 (1H, s), 9.96 (1H, s), 9.09 (1H, s), 8.33 (1H, d, *J* = 9.5 Hz), 8.26 (1H, d,* J* = 9.5 Hz), 7.82 (1H, s), 7.69 (1H, m), 7.68 (1H, d, *J* = 2.0 Hz), 7.47 (1H, t, *J* = 8.0 Hz), 7.25 (1H, m), 7.08 (1H, s), 6.17 (2H, s), 4.92 (2H, t,* J* = 6.0 Hz), 4.02 (3H, s), 3.20 (2H, t, *J* = 6.5 Hz); ^13^C NMR (125 MHz, DMSO-d_6_): 163.5, 158.0, 150.4, 150.0, 147.7, 144.5, 138.1, 133.7, 133.0, 130.9, 130.1, 129.0, 127.0, 125.9, 121.7, 121.3, 121.0, 120.7, 120.4, 116.8, 108.4, 105.6, 102.2, 57.3, 53.3, 26.2; HRMS (ESI) calcd for C_26_H_20_ClNO_6_ [M–Cl]^+^ 442.12851, found 442.1297.

*9-((3,4-dimethoxybenzoyl)oxy)-10-methoxy-5,6-dihydro-[1,3]dioxolo[4,5-g]isoquinolino[3,2-a]isoquinolin-7-ium chloride (****6****):* Brown powder (0.32 g, 61%); ^1^H NMR (500 MHz, DMSO-d_6_): δ ppm 9.89 (1H, s), 9.03 (1H, s), 8.26 (1H, d, *J* = 9.0 Hz), 8.20 (1H, d,* J* = 9.0 Hz), 7.86 (1H, dd,* J* = 8.5, 2.0 Hz), 7.76 (1H, s), 7.63 (1H, d, *J* = 2.5 Hz), 7.18 (1H, d, *J* = 8.5 Hz), 7.02 (1H, s), 6.12 (2H, s), 4.87 (2H, t,* J* = 6.5 Hz), 3.96 (3H, s), 3.86 (3H, s), 3.82 (3H, s), 3.14 (2H, t, *J* = 6.5 Hz); ^13^C NMR (125 MHz, DMSO-d_6_): 163.2, 154.2, 150.6, 150.0, 148.7, 147.8, 144.5, 138.1, 133.8, 133.0, 130.9, 126.9, 125.9, 125.0, 121.4, 120.7, 120.4, 119.8, 112.6, 111.5, 108.4, 105.6, 102.2, 57.3, 56.0, 55.8, 55.3, 26.2.

*9-((benzo[d][1,3]dioxole-5-carbonyl)oxy)-10-methoxy-5,6-dihydro-[1,3]dioxolo[4,5-g]isoquinolino[3,2-a]isoquinolin-7-ium chloride (****7****):* Brown powder (0.188 g, 37%); ^1^H NMR (500 MHz, DMSO-d_6_): δ ppm 9.92 (1H, s), 9.04 (1H, s), 8.31 (1H, d, *J* = 9.0 Hz), 8.25 (1H, d,* J* = 9.0 Hz), 7.88 (1H, dd,* J* = 8.0, 1.5 Hz), 7.81 (1H, s), 7.69 (1H, d, *J* = 2.0 Hz), 7.20 (1H, d, *J* = 8.0 Hz), 7.08 (1H, s), 6.23 (2H, s), 6.17 (2H, s), 4.90 (2H, t,* J* = 6.0 Hz), 4.01 (3H, s), 3.19 (2H, t, *J* = 6.5 Hz); ^13^C NMR (125 MHz, DMSO-d_6_): 163.5, 153.1, 150.9, 150.5, 148.3, 148.2, 144.8, 138.6, 134.0, 133.4, 131.4, 127.5, 127.4, 126.2, 121.8, 121.7, 121.0, 120.7, 110.1, 109.2, 108.9, 106.0, 103.0, 102.6, 57.7, 55.9, 26.6.

*10-methoxy-9-((3,4,5-trimethoxybenzoyl)oxy)-5,6-dihydro-[1,3]dioxolo[4,5-g]isoquinolino[3,2-a]isoquinolin-7-ium chloride (****8****):* Brown powder (0.069 g, 12%); ^1^H NMR (500 MHz, DMSO-d_6_): δ ppm 9.96 (1H, s), 9.07 (1H, s), 8.32 (1H, d, *J* = 9.5 Hz), 8.27 (1H, d,* J* = 9.5 Hz), 7.81 (1H, s), 7.53 (2H, s), 7.08 (1H, s), 6.17 (2H, s), 4.92 (2H, t,* J* = 6.0 Hz), 4.02 (3H, s), 3.91 (6H, s), 3.81 (3H, s), 3.19 (2H, t, *J* = 6.5 Hz); ^13^C NMR (125 MHz, DMSO-d_6_): 163.1, 153.1, 150.6, 150.1, 147.8, 144.6, 143.0, 138.3, 133.6, 133.1, 131.0, 127.2, 125.9, 122.9, 121.3, 120.8, 120.5, 108.6, 107.9, 105.7, 102.3, 60.5, 57.4, 56.4, 55.4, 26.3.

*9-((2-(benzyloxy)benzoyl)oxy)-10-methoxy-5,6-dihydro-[1,3]dioxolo[4,5-g]isoquinolino[3,2-a]isoquinolin-7-ium chloride (****9****):* Yellow powder (0.171 g, 30%); ^1^H NMR (500 MHz, DMSO-d_6_): δ ppm 9.86 (1H, s), 9.08 (1H, s), 8.32 (1H, d, *J* = 9.5 Hz), 8.26 (1H, d,* J* = 9.5 Hz), 8.24 (1H, dd,* J* = 8.0, 2.0 Hz), 7.82 (1H, s), 7.75 (1H, td, *J* = 9.0, 2.0 Hz), 7.52 (2H, d, *J* = 7.0 Hz), 7.42 (1H, d,* J* = 9.0 Hz), 7.33 (2H, t,* J* = 7.5 Hz), 7.27 (1H, t, *J* = 7.0 Hz), 7.21 (1H, t, *J* = 7.0 Hz), 7.07 (1H, s), 6.17 (2H, s), 5.31 (2H, s), 4.85 (2H, t,* J* = 5.5 Hz), 4.01 (3H, s), 3.17 (2H, t, *J* = 6.5 Hz); ^13^C NMR (125 MHz, DMSO-d_6_): 161.8, 159.1, 150.5, 150.0, 147.8, 144.4, 138.1, 136.8, 135.7, 133.8, 133.1, 132.9, 130.9, 128.4, 127.8, 127.2, 126.8, 126.0, 121.4, 120.8, 120.6, 120.4, 117.2, 114.3, 108.5, 105.6, 102.2, 69.8, 57.3, 55.4, 26.2; HRMS (ESI) calcd for C_33_H_26_ClNO_6_ [M–Cl]^+^ 532.17546, found 532.1761.

*9-((2-chlorobenzoyl)oxy)-10-methoxy-5,6-dihydro-[1,3]dioxolo[4,5-g]isoquinolino[3,2-a]isoquinolin-7-ium chloride (****10****):* Yellow powder (0.077 g, 16%); ^1^H NMR (500 MHz, DMSO-d_6_): δ ppm 10.00 (1H, s), 9.09 (1H, s), 8.43 (1H, dd, *J* = 8.0, 2.0 Hz), 8.33 (1H, d,* J* = 9.0 Hz), 8.28 (1H, d,* J* = 9.5 Hz), 7.81 (1H, s), 7.79 (1H, td, *J* = 8.0, 2.0 Hz), 7.75 (1H, dd, *J* = 7.5, 1.5 Hz), 7.66 (1H, td,* J* = 7.5, 2.0 Hz), 7.08 (1H, s), 6.17 (2H, s), 4.94 (2H, t,* J* = 6.0 Hz), 4.05 (3H, s), 3.21 (2H, t, *J* = 6.5 Hz); ^13^C NMR (125 MHz, DMSO-d_6_): 161.6, 150.3, 150.1, 147.8, 144.5, 138.3, 135.0, 134.0, 133.3, 133.2, 133.1, 131.7, 131.0, 127.8, 127.3, 127.0, 126.0, 121.1, 120.8, 120.4, 108.5, 105.7, 102.2, 57.5, 55.4, 26.2; HRMS (ESI) calcd for C_26_H_19_Cl_2_NO_5_ [M–Cl]^+^ 460.09463, found 460.09953.

*9-((3-chlorobenzoyl)oxy)-10-methoxy-5,6-dihydro-[1,3]dioxolo[4,5-g]isoquinolino[3,2-a]isoquinolin-7-ium chloride (****11****):* Yellow powder (0.12 g, 24%); ^1^H NMR (500 MHz, DMSO-d_6_): δ ppm 10.02 (1H, s), 9.08 (1H, s), 8.33 (1H, d, *J* = 9.5 Hz), 8.28 (1H, d,* J* = 9.5 Hz), 8.26 (1H, d,* J* = 4.0 Hz), 8.21 (1H, dd, *J* = 8.0, 3.0 Hz), 7.93 (1H, dd, *J* = 8.0, 3.0 Hz), 7.81 (1H, s), 7.74 (1H, t, *J* = 8.0 Hz), 7.08 (1H, s), 6.17 (2H, s), 4.90 (2H, t,* J* = 6.0 Hz), 4.02 (3H, s), 3.20 (2H, t, *J* = 6.0 Hz); ^13^C NMR (125 MHz, DMSO-d_6_): 162.4, 150.4, 150.1, 147.8, 144.6, 138.3, 134.5, 133.9, 133.3, 133.1, 131.3, 131.0, 130.1, 129.9, 129.2, 127.3, 125.9, 121.1, 120.7, 120.4, 108.5, 105.7, 102.2, 57.4, 55.4, 26.2; HRMS (ESI) calcd for C_26_H_19_Cl_2_NO_5_ [M–Cl]^+^ 460.09463, found 460.09963.

*9-((4-chlorobenzoyl)oxy)-10-methoxy-5,6-dihydro-[1,3]dioxolo[4,5-g]isoquinolino[3,2-a]isoquinolin-7-ium chloride (****12****):* Brown powder (0.034 g, 7%); ^1^H NMR (500 MHz, DMSO-d_6_): δ ppm 9.97 (1H, s), 9.06 (1H, s), 8.30 (1H, d, *J* = 9.0 Hz), 8.24 (1H, d,* J* = 9.0 Hz), 8.23 (2H, d,* J* = 8.5 Hz), 7.78 (1H, s), 7.74 (2H, d, *J* = 8.0 Hz), 7.04 (1H, s), 6.14 (2H, s), 4.86 (2H, t,* J* = 7.0 Hz), 3.98 (3H, s), 3.16 (2H, t, *J* = 6.5 Hz); ^13^C NMR (125 MHz, DMSO-d_6_): 162.7, 150.4, 150.0, 147.7, 144.5, 139.6, 138.2, 133.3, 133.0, 132.3, 130.9, 129.3, 127.2, 126.8, 125.9, 121.2, 120.7, 120.4, 108.4, 105.6, 102.2, 57.3, 55.3, 26.2.

*9-((2-fluorobenzoyl)oxy)-10-methoxy-5,6-dihydro-[1,3]dioxolo[4,5-g]isoquinolino[3,2-a]isoquinolin-7-ium chloride (****13****):* Yellow powder (0.207 g, 43%); ^1^H NMR (500 MHz, DMSO-d_6_): δ ppm 10.01 (1H, s), 9.10 (1H, s), 8.34 (1H, d, *J* = 9.5 Hz), 8.28 (2H, m), 7.89 (1H, m), 7.82 (1H, s), 7.53 (1H, t, *J* = 8.0 Hz), 7.51 (1H, t, *J* = 7.0 Hz), 7.08 (1H, s), 6.17 (2H, s), 4.92 (2H, t,* J* = 6.5 Hz), 4.04 (3H, s), 3.21 (2H, t, *J* = 6.0 Hz); ^13^C NMR (125 MHz, DMSO-d_6_): 163.5, 161.4, 161.1, 161.0, 150.9, 150.5, 148.2, 145.0, 138.7, 137.5, 137.4, 133.7, 133.5, 133.4, 131.4, 127.7, 126.4, 125.6, 125.5, 121.6, 121.2, 120.9, 118.1, 118.0, 116.9, 116.8, 109.0, 106.1, 102.7, 57.9, 55.8, 26.7; HRMS (ESI) calcd for C_26_H_19_ClFNO_5_ [M–Cl]^+^ 444.12418, found 444.1247.

*9-((3-fluorobenzoyl)oxy)-10-methoxy-5,6-dihydro-[1,3]dioxolo[4,5-g]isoquinolino[3,2-a]isoquinolin-7-ium chloride (****14****):* Yellow powder (0.278 g, 58%); ^1^H NMR (500 MHz, DMSO-d_6_): δ ppm 9.96 (1H, s), 9.04 (1H, s), 8.25 (1H, d, *J* = 9.0 Hz), 8.20 (1H, d,* J* = 9.0 Hz), 8.04 (1H, d,* J* = 7.5 Hz), 7.96 (1H, dt, *J* = 9.0, 2.5 Hz), 7.73 (1H, s), 7.68 (1H, dd, *J* = 13.5, 8.5 Hz), 7.65 (1H, td, *J* = 8.5, 3.0 Hz), 6.99 (1H, s), 6.08 (2H, s), 4.84 (2H, t,* J* = 6.0 Hz), 3.94 (3H, s), 3.12 (2H, t, *J* = 6.5 Hz); ^13^C NMR (125 MHz, DMSO-d_6_): 163.1, 162.3, 161.1, 150.4, 150.0, 147.7, 144.5, 138.2, 133.2, 133.0, 131.5, 131.4, 130.9, 130.3, 130.2, 127.2, 126.7, 125.9, 121.8, 121.6, 121.0, 120.7, 120.3, 117.1, 116.9, 108.4, 105.6, 102.2, 57.4, 55.3, 26.2; HRMS (ESI) calcd for C_26_H_19_ClFNO_5_ [M–Cl]^+^ 444.12418, found 444.1247.

*9-((4-fluorobenzoyl)oxy)-10-methoxy-5,6-dihydro-[1,3]dioxolo[4,5-g]isoquinolino[3,2-a]isoquinolin-7-ium chloride (****15****):* Yellow powder (0.099 g, 21%); ^1^H NMR (500 MHz, DMSO-d_6_): δ ppm 10.01 (1H, s), 9.10 (1H, s), 8.34 (3H, m), 8.28 (1H, d,* J* = 9.5 Hz), 7.83 (1H, s), 7.55 (2H, t, *J* = 8.5 Hz), 7.09 (1H, s), 6.18 (2H, s), 4.91 (2H, t,* J* = 6.5 Hz), 4.03 (3H, s), 3.20 (2H, t, *J* = 6.0 Hz); ^13^C NMR (125 MHz, DMSO-d_6_): 167.0, 164.9, 162.5, 150.4, 150.0, 147.8, 144.5, 138.2, 133.6, 133.5, 133.4, 133.0, 130.9, 127.1, 125.9, 124.6, 121.2, 120.7, 120.4, 116.5, 116.3, 108.5, 105.6, 102.2, 57.3, 55.3, 26.2; HRMS (ESI) calcd for C_26_H_19_ClFNO_5_ [M–Cl]^+^ 444.12418, found 444.1247.

*9-((2-bromobenzoyl)oxy)-10-methoxy-5,6-dihydro-[1,3]dioxolo[4,5-g]isoquinolino[3,2-a]isoquinolin-7-ium chloride (****16****):* Brown powder (0.165 g, 30%); ^1^H NMR (500 MHz, DMSO-d_6_): δ ppm 9.97 (1H, s), 9.06 (1H, s), 8.42 (1H, d, *J* = 7.0 Hz), 8.33 (1H, d,* J* = 9.0 Hz), 8.28 (1H, d,* J* = 9.5 Hz), 7.93 (1H, d,* J* = 8.5 Hz), 7.81 (1H, s), 7.70 (1H, t, *J* = 8.5 Hz), 7.69 (1H, t,* J* = 8.5 Hz), 7.08 (1H, s), 6.17 (2H, s), 4.93 (2H, s), 4.05 (3H, s), 3.20 (2H, t, *J* = 5.5 Hz); ^13^C NMR (125 MHz, DMSO-d_6_): 162.2, 150.4, 150.2, 147.9, 144.5, 138.4, 135.1, 133.4, 133.3, 133.2, 131.0, 129.0, 128.3, 127.4, 126.0, 122.5, 121.1, 120.8, 120.5, 108.6, 105.7, 102.3, 57.5, 55.5, 26.3; HRMS (ESI) calcd for C_26_H_19_ClBrNO_5_ [M–Cl]^+^ 504.04411, found 504.0447.

*9-((2-iodobenzoyl)oxy)-10-methoxy-5,6-dihydro-[1,3]dioxolo[4,5-g]isoquinolino[3,2-a]isoquinolin-7-ium chloride (****17****):* Yellow powder (0.2 g, 34%); ^1^H NMR (500 MHz, DMSO-d_6_): δ ppm 9.99 (1H, s), 9.08 (1H, s), 8.41 (1H, d, *J* = 8.0 Hz), 8.35 (1H, d,* J* = 9.5 Hz), 8.28 (1H, d,* J* = 9.0 Hz), 8.22 (1H, dd,* J* = 7.5, 1.5 Hz), 7.83 (1H, s), 7.71 (1H, t, *J* = 7.5 Hz), 7.47 (1H, td,* J* = 7.5, 2.0 Hz), 7.10 (1H, s), 6.18 (2H, s), 4.94 (2H, t, *J* = 6.0 Hz), 4.06 (3H, s), 3.21 (2H, t, *J* = 6.5 Hz); ^13^C NMR (125 MHz, DMSO-d_6_): 162.8, 150.4, 150.2, 147.9, 144.5, 142.0, 138.4, 134.8, 133.5, 133.2, 132.8, 131.9, 131.1, 128.7, 127.3, 126.0, 121.2, 120.8, 120.5, 108.6, 105.7, 102.3, 96.8, 57.5, 55.5, 26.3; HRMS (ESI) calcd for C_26_H_19_ClINO_5_ [M–Cl]^+^ 552.03024, found 552.0307.

*10-methoxy-9-((2-(trifluoromethyl)benzoyl)oxy)-5,6-dihydro-[1,3]dioxolo[4,5-g]isoquinolino[3,2-a]isoquinolin-7-ium chloride (****18****):* Yellow powder (0.206 g, 39%); ^1^H NMR (500 MHz, DMSO-d_6_): δ ppm 9.96 (1H, s), 9.13 (1H, s), 8.62 (1H, d, *J* = 7.0 Hz), 8.35 (1H, d, *J* = 9.5 Hz), 8.30 (1H, d, *J* = 9.0 Hz), 8.07 (1H, m), 8.01 (2H, m), 7.83 (1H, s), 7.09 (1H, s), 6.18 (2H, s), 4.98 (2H, t,* J* = 6.5 Hz), 4.05 (3H, s), 3.22 (2H, t, *J* = 6.5 Hz); ^13^C NMR (125 MHz, DMSO-d_6_): 161.9, 150.4, 150.0, 147.7, 144.1, 138.3, 133.8, 133.1, 133.0, 132.9, 132.5, 130.9, 127.6, 127.5, 127.4, 126.0, 120.9, 120.8, 120.3, 108.6, 108.5, 108.4, 105.6, 102.2, 57.3, 55.5, 26.2; HRMS (ESI) calcd for C_27_H_19_ClF_3_NO_5_ [M–Cl]^+^ 494.12098, found 494.1216.

*10-methoxy-9-((2-nitrobenzoyl)oxy)-5,6-dihydro-[1,3]dioxolo[4,5-g]isoquinolino[3,2-a]isoquinolin-7-ium chloride (****19****):* Yellow powder (0.162 g, 32%); ^1^H NMR (500 MHz, DMSO-d_6_): δ ppm 9.92 (1H, s), 9.12 (1H, s), 8.46 (1H, m), 8.35 (1H, d,* J* = 9.0 Hz), 8.30 (1H, d,* J* = 9.0 Hz), 8.18 (1H, m), 8.03 (2H, m), 7.83 (1H, s), 7.09 (1H, s), 6.18 (2H, s), 4.95 (2H, t, *J* = 6.0 Hz), 4.09 (3H, s), 3.22 (2H, t, *J* = 6.5 Hz); ^13^C NMR (125 MHz, DMSO-d_6_): 161.0, 150.4, 150.1, 149.1, 147.8, 144.0, 138.4, 134.9, 133.2, 133.1, 132.4, 131.6, 130.9, 127.6, 126.0, 124.3, 122.5, 121.2, 120.8, 120.3, 108.5, 105.6, 102.2, 57.5, 55.6, 26.2.

*9-((4-cyanobenzoyl)oxy)-10-methoxy-5,6-dihydro-[1,3]dioxolo[4,5-g]isoquinolino[3,2-a]isoquinolin-7-ium chloride (****20****):* Brown powder (0.138 g, 28%); ^1^H NMR (500 MHz, DMSO-d_6_): δ ppm 10.02 (1H, s), 9.07 (1H, s), 8.42 (2H, d, *J* = 9.0 Hz), 8.34 (1H, d,* J* = 9.5 Hz), 8.29 (1H, d,* J* = 9.0 Hz), 8.18 (2H, d, *J* = 8.5 Hz), 7.82 (1H, s), 7.09 (1H, s), 6.17 (2H, s), 4.88 (2H, t,* J* = 6.0 Hz), 4.03 (3H, s), 3.20 (2H, t, *J* = 6.5 Hz); ^13^C NMR (125 MHz, DMSO-d_6_): 162.4, 150.4, 150.2, 147.8, 144.8, 138.4, 133.2, 133.1, 133.0, 132.0, 131.1, 131.0, 127.4, 126.0, 121.1, 120.7, 120.4, 118.1, 116.6, 108.5, 105.7, 102.2, 57.5, 55.4, 26.2; HRMS (ESI) calcd for C_27_H_19_ClN_2_O_5_ [M–Cl]^+^ 451.12885, found 451.1288.

*9-((2,6-dichlorobenzoyl)oxy)-10-methoxy-5,6-dihydro-[1,3]dioxolo[4,5-g]isoquinolino[3,2-a]isoquinolin-7-ium chloride (****21****):* Yellow powder (0.098 g, 18%); ^1^H NMR (500 MHz, DMSO-d_6_): δ ppm 9.64 (1H, s), 9.12 (1H, s), 8.40 (1H, d, *J* = 9.0 Hz), 8.32 (1H, d,* J* = 9.5 Hz), 7.83 (1H, s), 7.79 (1H, s), 7.78 (1H, d, *J* = 8.5 Hz), 7.71 (1H, dd, *J* = 8.5, 7.0 Hz), 7.11 (1H, s), 6.19 (2H, s), 4.91 (2H, t,* J* = 6.5 Hz), 4.12 (3H, s), 3.22 (2H, t, *J* = 7.0 Hz); ^13^C NMR (125 MHz, DMSO-d_6_): 161.3, 151.2, 150.8, 143.6, 139.1, 134.0, 133.9, 132.4, 131.9, 131.8, 131.5, 129.5, 129.4, 128.3, 128.2, 126.4, 124.0, 121.3, 120.8, 120.4, 108.8, 106.0, 102.8, 57.6, 57.2, 26.2; HRMS (ESI) calcd for C_26_H_18_Cl_3_NO_5_ [M–Cl]^+^ 494.05565, found 494.0565.

*10-methoxy-9-((2-methylbenzoyl)oxy)-5,6-dihydro-[1,3]dioxolo[4,5-g]isoquinolino[3,2-a]isoquinolin-7-ium chloride (****22****):* Yellow powder (0.014 g, 3%); ^1^H NMR (500 MHz, DMSO-d_6_): δ ppm 10.00 (1H, s), 9.09 (1H, s), 8.34 (1H, d, *J* = 9.5 Hz), 8.32 (1H, d,* J* = 7.0 Hz), 8.27 (1H, d,* J* = 9.0 Hz), 7.83 (1H, s), 7.68 (1H, t, *J* = 7.5 Hz), 7.51 (1H, d, *J* = 7.5 Hz), 7.50 (1H, t,* J* = 8.0 Hz), 7.09 (1H, s), 6.18 (2H, s), 4.92 (2H, t,* J* = 6.0 Hz), 4.04 (3H, s), 3.20 (2H, t, *J* = 6.0 Hz), 2.64 (3H, s); ^13^C NMR (125 MHz, DMSO-d_6_): 164.4, 150.9, 150.5, 148.2, 145.1, 141.6, 138.6, 134.3, 134.2, 133.5, 132.6, 132.2, 131.4, 127.7, 127.4, 126.9, 126.4, 121.8, 121.2, 120.9, 109.0, 106.1, 102.7, 57.8, 55.8, 26.7, 21.9; HRMS (ESI) calcd for C_27_H_22_ClNO_5_ [M–Cl]^+^ 440.14925, found 440.1498.

*10-methoxy-9-((4-methylbenzoyl)oxy)-5,6-dihydro-[1,3]dioxolo[4,5-g]isoquinolino[3,2-a]isoquinolin-7-ium chloride (****23****):* Yellow powder (0.2 g, 42%); ^1^H NMR (500 MHz, DMSO-d_6_): δ ppm 9.96 (1H, s), 9.09 (1H, s), 8.33 (1H, d, *J* = 9.0 Hz), 8.27 (1H, d,* J* = 9.0 Hz), 8.16 (2H, d,* J* = 8.0 Hz), 7.83 (1H, s), 7.50 (2H, d, *J* = 8.5 Hz), 7.08 (1H, s), 6.18 (2H, s), 4.91 (2H, t,* J* = 6.0 Hz), 4.02 (3H, s), 3.19 (2H, t, *J* = 6.5 Hz), 2.48 (3H, s); ^13^C NMR (125 MHz, DMSO-d_6_): 163.4, 150.5, 150.0, 147.7, 145.3, 144.5, 138.2, 133.7, 133.0, 130.9, 130.5, 129.7, 126.9, 125.9, 125.2, 121.3, 120.7, 120.4, 108.4, 105.6, 102.2, 57.3, 55.3, 26.2, 21.4.

*9-((4-ethylbenzoyl)oxy)-10-methoxy-5,6-dihydro-[1,3]dioxolo[4,5-g]isoquinolino[3,2-a]isoquinolin-7-ium chloride (****24****):* Yellow powder (0.017 g, 3%); ^1^H NMR (500 MHz, DMSO-d_6_): δ ppm 9.96 (1H, s), 9.05 (1H, s), 8.33 (1H, d, *J* = 9.0 Hz), 8.26 (1H, d,* J* = 9.5 Hz), 8.17 (2H, d,* J* = 8.0 Hz), 7.82 (1H, s), 7.53 (2H, d, *J* = 8.0 Hz), 7.08 (1H, s), 6.18 (2H, s), 4.89 (2H, t,* J* = 6.5 Hz), 4.01 (3H, s), 3.18 (2H, t, *J* = 6.5 Hz), 2.77 (2H, q, *J* = 7.5 Hz), 1.25 (3H, t, *J* = 7.5 Hz); ^13^C NMR (125 MHz, DMSO-d_6_): 163.6, 151.6, 150.6, 150.2, 147.9, 144.6, 138.3, 133.8, 133.1, 131.0, 130.8, 128.7, 127.1, 126.0, 125.6, 121.4, 120.8, 120.5, 108.6, 105.7, 102.3, 57.4, 55.4, 28.6, 26.3, 15.6; HRMS (ESI) calcd for C_28_H_24_ClNO_5_ [M–Cl]^+^ 454.16490, found 454.1654.

*9-((4-(tert-butyl)benzoyl)oxy)-10-methoxy-5,6-dihydro-[1,3]dioxolo[4,5-g]isoquinolino[3,2-a]isoquinolin-7-ium chloride (****25****):* Yellow powder (0.174 g, 34%); ^1^H NMR (500 MHz, DMSO-d_6_): δ ppm 9.95 (1H, s), 9.04 (1H, s), 8.32 (1H, d, *J* = 9.0 Hz), 8.26 (1H, d,* J* = 9.0 Hz), 8.19 (2H, d,* J* = 8.0 Hz), 7.82 (1H, s), 7.71 (2H, d, *J* = 8.5 Hz), 7.08 (1H, s), 6.17 (2H, s), 4.89 (2H, t,* J* = 6.5 Hz), 4.01 (3H, s), 3.18 (2H, t, *J* = 6.0 Hz), 1.36 (9H, s); ^13^C NMR (125 MHz, DMSO-d_6_): 163.5, 158.1, 150.6, 150.1, 147.8, 144.6, 138.3, 133.7, 133.1, 131.0, 130.5, 127.1, 126.1, 126.0, 125.3, 121.4, 120.8, 120.5, 108.6, 105.7, 102.3, 57.4, 55.3, 35.2, 30.9, 26.2.

*9-((3,5-di-tert-butylbenzoyl)oxy)-10-methoxy-5,6-dihydro-[1,3]dioxolo[4,5-g]isoquinolino[3,2-a]isoquinolin-7-ium chloride (****26****):* Yellow powder (0.205 g, 36%); ^1^H NMR (500 MHz, DMSO-d_6_): δ ppm 9.99 (1H, s), 9.07 (1H, s), 8.34 (1H, d, *J* = 9.5 Hz), 8.27 (1H, d,* J* = 9.5 Hz), 8.06 (2H, d,* J* = 2.0 Hz), 7.90 (1H, t,* J* = 2.0 Hz), 7.83 (1H, s), 7.09 (1H, s), 6.18 (2H, s), 4.90 (2H, t,* J* = 5.5 Hz), 4.03 (3H, s), 3.19 (2H, t, *J* = 6.0 Hz), 1.38 (18H, s); ^13^C NMR (125 MHz, DMSO-d_6_): 164.1, 158.4, 151.5, 150.1, 147.8, 144.6, 138.2, 133.8, 133.0, 130.9, 129.0, 127.3, 126.8, 125.6, 124.2, 121.4, 120.7, 120.4, 108.5, 105.6, 102.2, 57.3, 55.5, 34.8, 31.1, 26.2; HRMS (ESI) calcd for C_34_H_36_ClNO_5_ [M–Cl]^+^ 538.25880, found 538.2592.

*9-((1-naphthoyl)oxy)-10-methoxy-5,6-dihydro-[1,3]dioxolo[4,5-g]isoquinolino[3,2-a]isoquinolin-7-ium chloride (****27****):* Yellow powder; (0.172 g, 34%); ^1^H NMR (500 MHz, DMSO-d_6_): δ ppm 10.07 (1H, s), 9.13 (1H, s), 8.91 (1H, d, *J* = 8.5 Hz), 8.74 (1H, d, *J* = 7.5 Hz), 8.41 (1H, d,* J* = 8.5 Hz), 8.37 (1H, d,* J* = 9.5 Hz), 8.31 (1H, d, *J* = 9.5 Hz), 8.16 (1H, d, *J* = 8.0 Hz), 7.84 (1H, s), 7.80 (1H, t,* J* = 7.5 Hz), 7.75 (1H, t,* J* = 7.0 Hz), 7.69 (1H, t,* J* = 8.0 Hz), 7.08 (1H, s), 6.18 (2H, s), 4.93 (2H, t,* J* = 6.5 Hz), 4.07 (3H, s), 3.20 (2H, t, *J* = 6.5 Hz); ^13^C NMR (125 MHz, DMSO-d_6_): 163.8, 150.5, 150.0, 147.7, 144.6, 138.2, 135.1, 133.8, 133.6, 133.1, 132.3, 131.0, 130.9, 129.1, 128.6, 127.0, 126.8, 125.9, 125.1, 125.0, 124.1, 121.4, 120.7, 120.4, 108.4, 105.6, 102.2, 57.4, 55.3, 26.2.

*9-((2-naphthoyl)oxy)-10-methoxy-5,6-dihydro-[1,3]dioxolo[4,5-g]isoquinolino[3,2-a]isoquinolin-7-ium chloride (****28****):* Yellow powder (0.155 g, 30%); ^1^H NMR (500 MHz, DMSO-d_6_): δ ppm 10.06 (1H, s), 9.13 (1H, s), 9.01 (1H, s), 8.36 (1H, d, *J* = 9.5 Hz), 8.30 (1H, d,* J* = 9.5 Hz), 8.26 (1H, d,* J* = 8.0 Hz), 8.23 (1H, d, *J* = 8.5 Hz), 8.20 (1H, d, *J* = 9.0 Hz), 8.12 (1H, d, *J* = 8.0 Hz), 7.84 (1H, s), 7.78 (1H, t,* J* = 7.0 Hz), 7.71 (1H, t,* J* = 7.0 Hz), 7.08 (1H, s), 6.18 (2H, s), 4.92 (2H, t,* J* = 7.0 Hz), 4.04 (3H, s), 3.20 (2H, t, *J* = 7.0 Hz); ^13^C NMR (125 MHz, DMSO-d_6_): 163.6, 150.5, 150.0, 147.7, 144.5, 138.2, 135.6, 133.7, 133.0, 132.4, 132.1, 130.9, 129.6, 129.4, 128.7, 127.9, 127.4, 127.0, 125.9, 125.4, 125.2, 121.3, 120.7, 120.4, 108.4, 105.6, 102.2, 57.3, 55.3, 26.2.

*9-(cinnamoyloxy)-10-methoxy-5,6-dihydro-[1,3]dioxolo[4,5-g]isoquinolino[3,2-a]isoquinolin-7-ium chloride (****29****):* Yellow powder (0.051 g, 10%); ^1^H NMR (500 MHz, DMSO-d_6_): δ ppm 9.94 (1H, s), 9.02 (1H, s), 8.29 (1H, d, *J* = 9.5 Hz), 8.24 (1H, d,* J* = 9.0 Hz), 7.99 (1H, d,* J* = 16.0 Hz), 7.86 (2H, m), 7.80 (1H, s), 7.51 (1H, s), 7.50 (2H, m), 7.07 (1H, s), 7.05 (1H, d,* J* = 16.0 Hz), 6.16 (2H, s), 4.92 (2H, t,* J* = 6.0 Hz), 4.03 (3H, s), 3.20 (2H, t, *J* = 6.5 Hz); ^13^C NMR (125 MHz, DMSO-d_6_): 163.9, 150.7, 150.2, 147.9, 147.8, 144.5, 138.3, 133.9, 133.7, 133.1, 131.5, 131.0, 129.4, 129.0, 127.0, 126.0, 121.4, 120.8, 120.5, 116.4, 108.6, 105.7, 102.3, 57.4, 55.5, 26.4.

*(E)-10-methoxy-9-((2-methyl-3-phenylacryloyl)oxy)-5,6-dihydro-[1,3]dioxolo[4,5-g]isoquinolino[3,2-a]isoquinolin-7-ium chloride (****30****):* Yellow powder (0.025 g, 5%); ^1^H NMR (500 MHz, DMSO-d_6_): δ ppm 9.95 (1H, s), 9.08 (1H, s), 8.32 (1H, d, *J* = 9.5 Hz), 8.25 (1H, d,* J* = 9.5 Hz), 8.05 (1H, s), 7.83 (1H, s), 7.66 (2H, d, *J* = 7.5 Hz), 7.53 (2H, t, *J* = 7.5 Hz), 7.47 (1H, t, *J* = 7.5 Hz), 7.10 (1H, s), 6.18 (2H, s), 4.96 (2H, t,* J* = 6.5 Hz), 4.05 (3H, s), 3.21 (2H, t, *J* = 6.5 Hz), 2.32 (3H, d, *J* = 9.5 Hz); ^13^C NMR (125 MHz, DMSO-d_6_): 165.9, 151.0, 150.5, 148.2, 145.0, 142.0, 138.6, 135.3, 134.4, 133.5, 131.4, 130.7, 129.9, 129.3, 127.3, 126.7, 126.4, 121.8, 121.2, 120.9, 109.0, 106.1, 102.7, 57.8, 55.8, 26.7, 15.0; HRMS (ESI) calcd for C_29_H_24_ClNO_5_ [M–Cl]^+^ 466.16490, found 466.1654.

*(E)-9-((3-(benzo[d][1,3]dioxol-5-yl)acryloyl)oxy)-10-methoxy-5,6-dihydro-[1,3]dioxolo[4,5-g]isoquinolino[3,2-a]isoquinolin-7-ium chloride (****31****):* Brown powder (0.108 g, 20%); ^1^H NMR (500 MHz, DMSO-d_6_): δ ppm 9.93 (1H, s), 9.05 (1H, s), 8.31 (1H, d, *J* = 9.5 Hz), 8.23 (1H, d,* J* = 9.0 Hz), 7.90 (1H, d,* J* = 16.0 Hz), 7.82 (1H, s), 7.59 (1H, d,* J* = 2.0 Hz), 7.36 (1H, dd, *J* = 8.5, 2.0 Hz), 7.09 (1H, s), 7.04 (1H, d, *J* = 8.0 Hz), 6.92 (1H, d,* J* = 16.0 Hz), 6.18 (2H, s), 6.14 (2H, s), 4.93 (2H, t,* J* = 6.0 Hz), 4.04 (3H, s), 3.21 (2H, t, *J* = 6.5 Hz); ^13^C NMR (125 MHz, DMSO-d_6_): 164.2, 150.8, 150.3, 150.2, 148.4, 147.9, 147.8, 144.6, 138.3, 133.8, 133.2, 131.1, 128.4, 127.0, 126.2, 126.0, 121.6, 120.8, 120.5, 114.0, 108.9, 108.7, 107.2, 105.8, 102.4, 102.1, 57.4, 55.6, 26.4.

*(E)-9-((3-(2,6-dichlorophenyl)acryloyl)oxy)-10-methoxy-5,6-dihydro-[1,3]dioxolo[4,5-g]isoquinolino[3,2-a]isoquinolin-7-ium chloride (****32****):* Brown powder (0.024 g, 4%); ^1^H NMR (500 MHz, DMSO-d_6_): δ ppm 9.96 (1H, s), 9.04 (1H, s), 8.32 (1H, d, *J* = 9.0 Hz), 8.26 (1H, d,* J* = 9.0 Hz), 8.03 (1H, d,* J* = 16.5 Hz), 7.81 (1H, s), 7.66 (2H, d, *J* = 8.5 Hz), 7.51 (1H, t, *J* = 8.5 Hz), 7.09 (1H, d, *J* = 16.5 Hz), 7.09 (1H, s), 6.17 (2H, s), 4.92 (2H, t,* J* = 6.5 Hz), 4.06 (3H, s), 3.21 (2H, t, *J* = 6.5 Hz); ^13^C NMR (125 MHz, DMSO-d_6_): 163.0, 150.2, 147.9, 144.3, 140.5, 139.9, 138.4, 134.4, 133.3, 132.1, 130.5, 129.6, 128.0, 127.4, 127.0, 126.0, 124.7, 121.3, 120.9, 120.4, 108.6, 105.6, 102.2, 57.4, 55.5, 26.3; HRMS (ESI) calcd for C_28_H_20_Cl_3_NO_5_ [M–Cl]^+^ 520.07130, found 520.07867.

*9-(((2E,4E)-5-(benzo[d][1,3]dioxol-5-yl)penta-2,4-dienoyl)oxy)-10-methoxy-5,6-dihydro-[1,3]dioxolo[4,5-g]isoquinolino[3,2-a]isoquinolin-7-ium chloride (****33****):* Yellow powder (0.219 g, 39%); ^1^H NMR (500 MHz, DMSO-d_6_): δ ppm 9.85 (1H, s), 8.98 (1H, s), 8.27 (1H, d, *J* = 9.0 Hz), 8.22 (1H, d,* J* = 9.0 Hz), 7.78 (1H, s), 7.69 (1H, ddd,* J* = 15.0, 7.5, 3.0 Hz), 7.30 (1H, d,* J* = 2.0 Hz), 7.20 (1H, d,* J* = 15.5 Hz), 7.16 (1H, d, *J* = 15.5 Hz), 7.09 (1H, dd, *J* = 8.0, 1.5 Hz), 7.07 (1H, s), 6.96 (1H, d, *J* = 7.5 Hz), 6.41 (1H, d,* J* = 15.5 Hz), 6.15 (2H, s), 6.06 (2H, s), 4.90 (2H, t,* J* = 6.0 Hz), 4.02 (3H, s), 3.19 (2H, t, *J* = 6.5 Hz); ^13^C NMR (125 MHz, DMSO-d_6_): 164.0, 160.8, 156.6, 150.8, 150.2, 148.8, 148.3, 148.0, 144.7, 142.6, 138.4, 133.7, 133.2, 131.2, 130.6, 126.1, 124.8, 124.1, 121.6, 120.9, 120.6, 117.7, 108.9, 108.7, 106.2, 106.0, 102.6, 101.8, 57.4, 55.6, 26.4; HRMS (ESI) calcd for C_31_H_24_ClNO_7_ [M–Cl]^+^ 522.15473, found 522.1556.

*9-(2-(2,6-dichlorophenyl)acetoxy)-10-methoxy-5,6-dihydro-[1,3]dioxolo[4,5-g]isoquinolino[3,2-a]isoquinolin-7-ium chloride (****34****):* Yellow powder (0.018 g, 3%); ^1^H NMR (500 MHz, DMSO-d_6_): δ ppm 9.96 (1H, s), 9.01 (1H, s), 8.24 (2H, s), 7.78 (1H, s), 7.57 (2H, d, *J* = 8.0 Hz), 7.42 (1H, t, *J* = 8.0 Hz), 7.09 (1H, s), 6.16 (2H, s), 4.96 (2H, s), 4.59 (2H, s), 3.98 (3H, s), 3.24 (2H, s); ^13^C NMR (125 MHz, DMSO-d_6_): 167.0, 150.5, 150.1, 147.8, 144.2, 138.3, 135.7, 133.2, 133.0, 131.0, 130.5, 130.4, 128.6, 127.2, 126.0, 121.0, 120.8, 120.4, 108.6, 105.7, 102.3, 57.2, 55.6, 36.2, 26.3; HRMS (ESI) calcd for C_27_H_20_Cl_3_NO_5_ [M–Cl]^+^ 508.07130, found 508.0720.

*9-(2,2-diphenylacetoxy)-10-methoxy-5,6-dihydro-[1,3]dioxolo[4,5-g]isoquinolino[3,2-a]isoquinolin-7-ium chloride (****35****):* Yellow powder (0.063 g, 11%); ^1^H NMR (500 MHz, DMSO-d_6_): δ ppm 10.04 (1H, s), 9.04 (1H, s), 8.22 (2H, m), 7.79 (1H, s), 7.57 (4H, m), 7.42 (4H, m), 7.33 (2H, m), 7.08 (1H, s), 6.16 (2H, s), 6.09 (1H, s), 4.99 (2H, t,* J* = 5.5 Hz), 4.00 (3H, s), 3.22 (2H, t, *J* = 6.0 Hz); ^13^C NMR (125 MHz, DMSO-d_6_): 169.6, 150.4, 150.0, 147.8, 144.3, 138.6, 138.1, 133.3, 133.0, 132.6, 130.9, 129.0, 128.7, 126.1, 124.2, 121.0, 120.6, 120.3, 115.1, 108.4, 105.6, 102.2, 57.0, 55.4, 25.6; HRMS (ESI) calcd for C_33_H_26_ClNO_5_ [M–Cl]^+^ 516.18055, found 516.1815.

#### α-Glucosidase inhibitory assay

Berberrubine derivatives will be assayed for yeast α-glucosidase inhibitory activity. The protocol described by Ramadhan et al*.* will be used^37^. Briefly, yeast α-glucosidase (0.1 U/mL) and substrate (1 mM *p*-nitrophenyl-α-D-glucopyranoside) were dissolved in 0.1 M phosphate buffer (pH 6.9). A 10 μL test sample was pre-incubated with α-glucosidase (40 μL) at 37 °C for 10 min. A substrate solution (50 μL) was then added to the reaction mixture and incubated at 37 °C for an additional 20 min, and terminated by adding 1 M Na_2_CO_3_ solution (100 μL). Enzymatic activity was quantified by measuring the absorbance at 405 nm (ALLSHENG AMR-100 microplate reader). The percentage inhibition of activity was calculated as follows: % Inhibition = [(A_0 _− A_1_)/A_0_] × 100, where: A_0_ is the absorbance without the sample; A_1_ is the absorbance with the sample. The IC_50_ value was deduced from the plot of % inhibition *versus* the concentration of the test sample. Acarbose was used as standard control and the experiment was performed in triplicate.

### Kinetic study of α-glucosidase inhibition

The mode of inhibition of α-glucosidase was determined from Lineweaver–Burk plots. The inhibition type was determined using various concentrations of p-NPG substrate in the absence or presence of compounds at different concentrations. The *K*_i_ value was determined from secondary plots of slope versus [I].

### Cells culture

HEK-293 cells (ATCC® CRL-1573™) were maintained in growth medium consisting of DMEM (Gibco®, Langley, OK) supplemented with 10% fetal bovine serum (Gibco®, Langley, OK), 100 I.U./ml penicillin (Bio Basic Canada®, Ontario, CA), 100 μg/ml streptomycin (Bio Basic Canada®, Ontario, CA), and 10 mM HEPES (4-(2-hydroxyethyl)-1-piperazineethanesulfonic acid) (Sigma Aldrich®, St. Louis, MO) at 37 °C under 5% CO_2_.

### Cytotoxicity study

The cytotoxicity of the active compounds (**9**, **26**, **28** and **33**) was tested with HEK-293 cells. The cells were seeded at 1 × 10^4^ cells per well into 96-well plates in growth medium and incubated overnight. The compounds were prepared at the indicated concentrations in dimethylsulfoxide (DMSO) and added to the cells. Cells were incubated for 48 h before analyzing the cell viability using CellTiter 96® AQueous One Solution Cell Proliferation Assay kit (Promega, USA) according to manufacturer’s protocol. The plate was read at the *A*_490_ by VICTORTM X3 microplate reader (PerkinElmer, USA). The 1%DMSO treated cells as a positive control (100% viability). The CC_50_ values were calculated from nonlinear regression analysis. Results were reported as means and standard error mean (SEM) from three biological independent experiments.

### Computational details

The 3D structure of α-glucosidase MAL12 derived from the alphafold2 database (UniProt ID: P53341^50^) was used as a protein receptor. The protonation state of protein was assigned at pH 7.0 using PDB2PQR^51^ and then the structure was minimized by SANDER^52^. The 3D structures of potent compounds **9**, **26**, **28**, and **33** were constructed by gaussview 6.0^53^, and subsequently optimized with density function of theory (DFT) with B3LYP/ 6-31G* basis set using gaussian 16^53^. The antechamber and parmchk2 tools were adopted to parameterize the ligand force field using GAFF^54^, while their partial charges were assigned using the RESP charge fitting method. To predict the binding pose and affinity of potent compounds at the active site of α-glucosidase, a molecular docking was applied using AutoDock Vina 1.2.3 software^55^. Then, molecular dynamics (MD) simulation for these complexes was performed using the AMBER20 package program^52^. In brief, the ligand/protein complex was prepared by adding all missing hydrogen atoms, neutralizing by ions, and solvating in the TIP3P^56^ water model with 12 Å from the protein surface by the tLeap module^52^. Then, the complex was slowly minimized and structurally relaxed by harmonic potentials, as previously described in our studies^57–59^. Each system was heated to 300 K for 20 ps with the canonical ensemble under periodic boundary conditions and then simulated for 300 ns. The production phase extracted from the last 100 ns-MD trajectories was selected to calculate the binding free energy using MM/GBSA and QM-MM/GBSA^60^ with three different theories AM1, PM3, and PM6 treated on ligand molecule. The key residues that interacted with the most potent compound were evaluated based on MM/GBSA method. The 3D structures and 2D binding interactions were visualized by UCSF Chimera V1.16^61^ and BIOVIA Discovery Studio Visualizer^62^.

The distribution functions of solvent were produced by 3D-RISM theory. The 3D-RISM equation was solved with the Kovalenko-Hirata closure^63^. The system temperature and the density of solvent water were set at 300 K and 1.0 g/cm^3^. The Lennard–Jones parameters for solute molecules were taken from the GAFF^64^ parameter set assigned by antechamber. The TIP3P^56^ arranged for the geometrical and potential parameters for the solvent water was employed with modified hydrogen parameters (σ = 0.4 Å and ε = 0.046 kcal/mol). The 3D grid spacing with 0.5 Å was assigned for the number of grid points with 128. The calculation was performed using the RISMiCal program package^65–67^.

### Supplementary Information

Supplementary Information.

## Data Availability

All data generated or analyzed during this study are included in this published article and its Supplementary Information files.
