# Supplementary material for: Structure—yeast α-glucosidase inhibitory activity relationship of 9-O-berberrubine carboxylates
Source: Sci Rep. 2023 Nov 1;13:18865. doi: 10.1038/s41598-023-45116-0 (PMC10620162; doi:10.1038/s41598-023-45116-0)
Supplement: Supplementary file 1 — Supplementary Information. [file 41598_2023_45116_MOESM1_ESM.pdf]

Structure – yeast α-glucosidase inhibitory activity relationship of 9-*O*-berberrubine carboxylates

Duy Vu Nguyen[1], Kowit Hengphasatporn[2], Ade Danova[1,3], Aphinya Suroengrit[4], Siwaporn Boonyasuppayakorn[4], Ryo Fujiki[2], Yasuteru Shigeta[2], Thanyada Rungrotmongkol[5,6], Warinthorn Chavasiri[1,*]

[1] Center of Excellence in Natural Products Chemistry, Department of Chemistry, Faculty of Science, Chulalongkorn University, Pathumwan, Bangkok, 10330, Thailand

[2] Center for Computational Sciences, University of Tsukuba, 1-1-1 Tennodai, Tsukuba, Ibaraki 305-8577, Japan

[3] Organic Chemistry Division, Department of Chemistry, Faculty of Mathematics and Natural Sciences, Institut Teknologi Bandung, West Java, 40132, Indonesia

[4] Center of Excellence in Applied Medical Virology, Department of Microbiology, Faculty of Medicine, Chulalongkorn University, Bangkok 10330, Thailand

[5] Bioinformatics and Computational Biology Program, Graduated School, Chulalongkorn University, Bangkok, 10330, Thailand

[6] Center of Excellence in Biocatalyst and Sustainable Biotechnology, Department of Biochemistry, Faculty of Science, Chulalongkorn University, Bangkok 10330, Thailand

* Corresponding author:

Assistant Professor Dr. Warinthorn Chavasiri

Mahamakut Building, Department of Chemistry, Faculty of Science, Chulalongkorn University, Phayathai Road, Pathumwan, Bangkok 10330, Thailand.

E-mail address: warinthorn.c@chula.ac.th

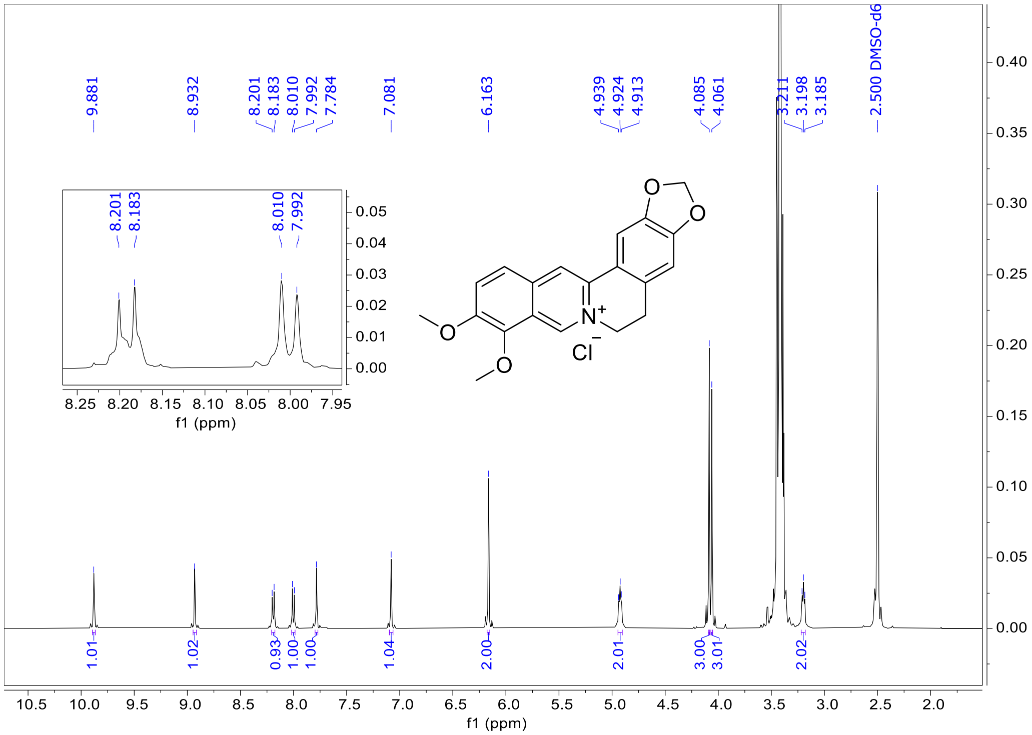

Figure S1: ^{1}H-NMR (500 MHz, DMSO-d_6) spectrum of **BBR**

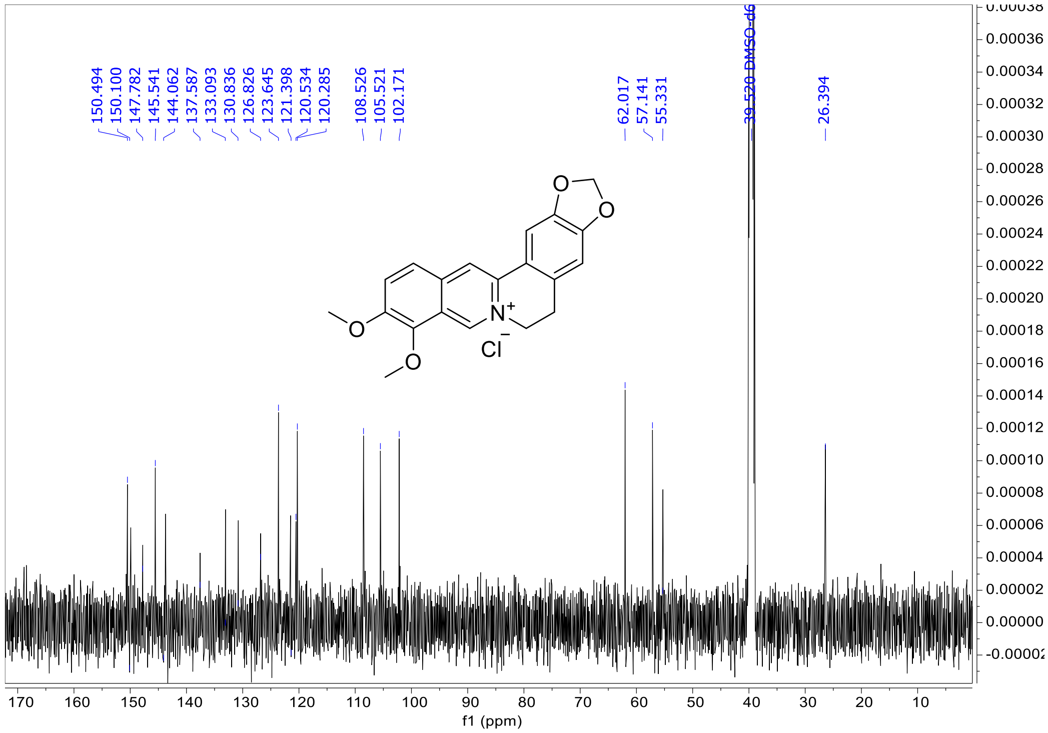

Figure S2: ^{13}C-NMR (125 MHz, DMSO-d_6) spectrum of **BBR**

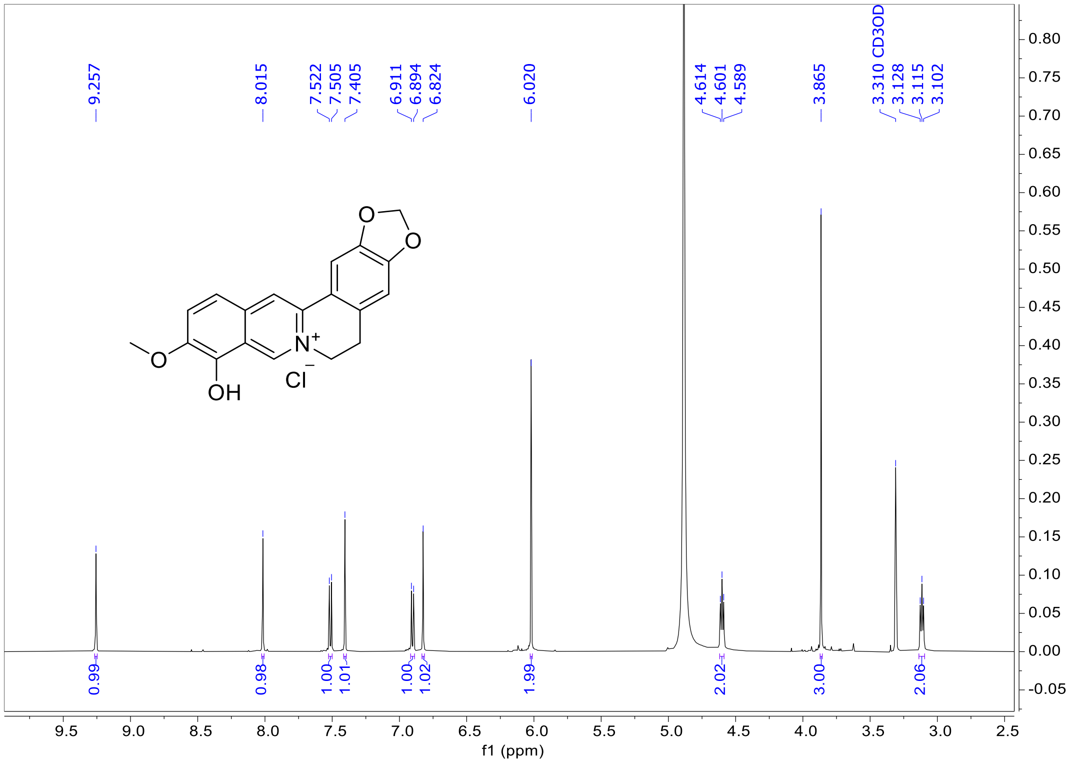

Figure S3: ^{1}H-NMR (500 MHz, DMSO-d_6) spectrum of **BBRB**

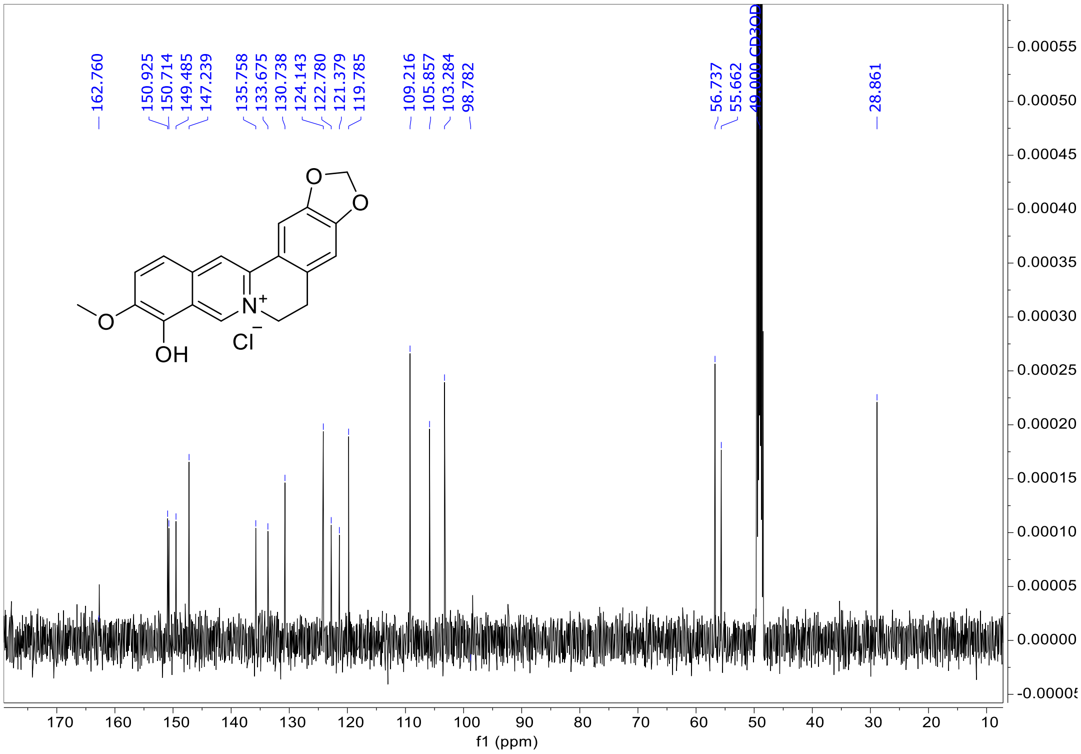

Figure S4: ^{13}C-NMR (125 MHz, DMSO-d_6) spectrum of **BBRB**

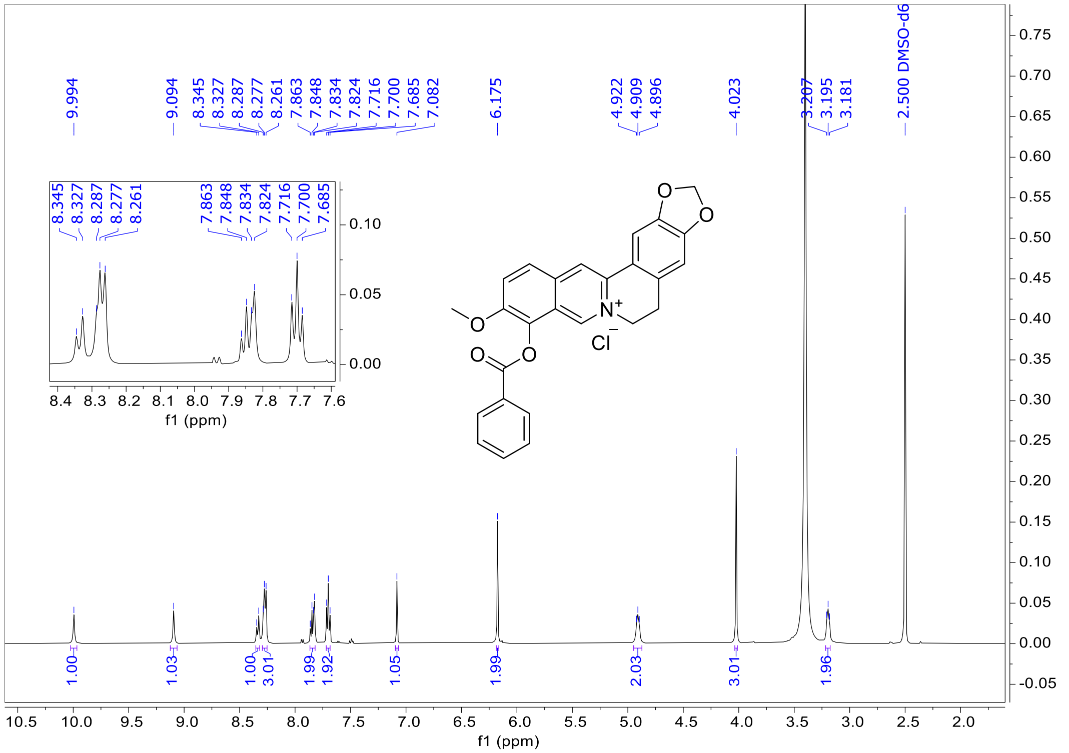

Figure S5: ^{1}H-NMR (500 MHz, DMSO-d_6) spectrum of compound **1**

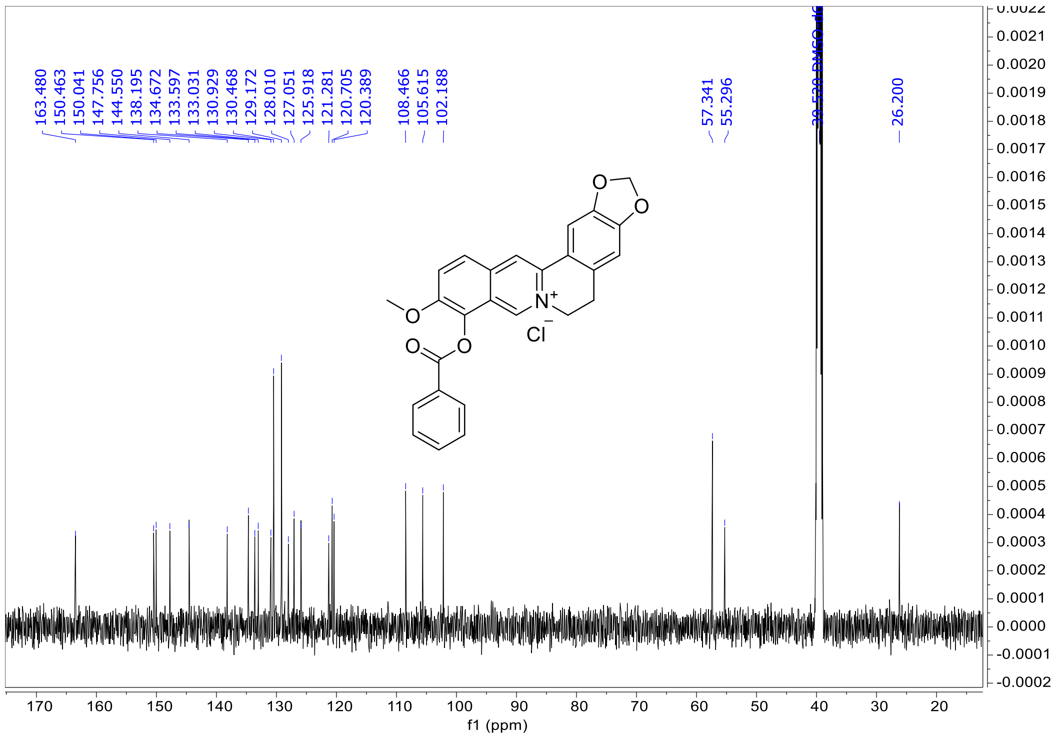

Figure S6: ^{13}C-NMR (125 MHz, DMSO-d_6) spectrum of compound **1**

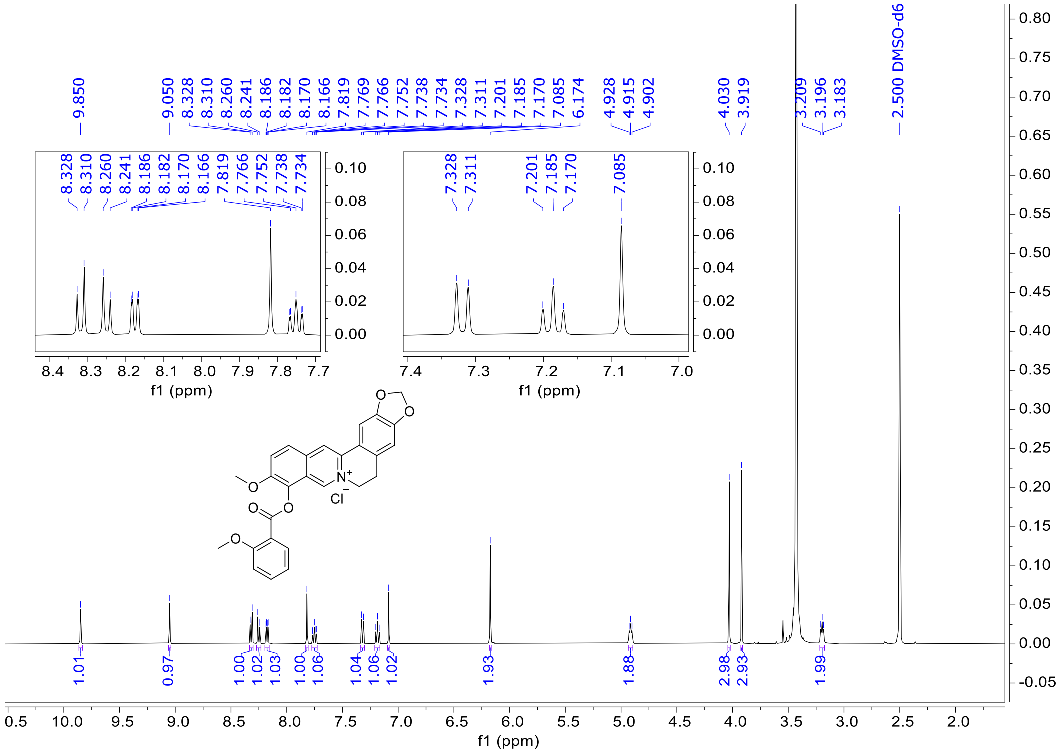

Figure S7: ^{1}H-NMR (500 MHz, DMSO-d_6) spectrum of compound **2**

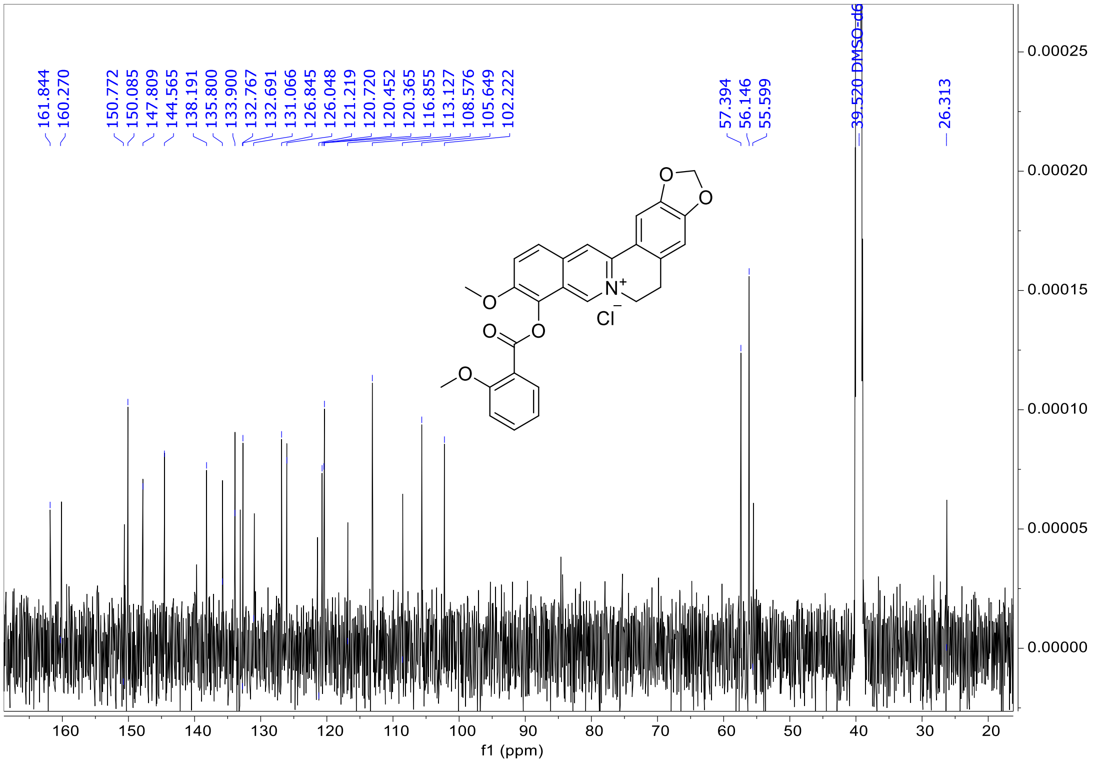

Figure S8: ^{13}C-NMR (125 MHz, DMSO-d_6) spectrum of compound **2**

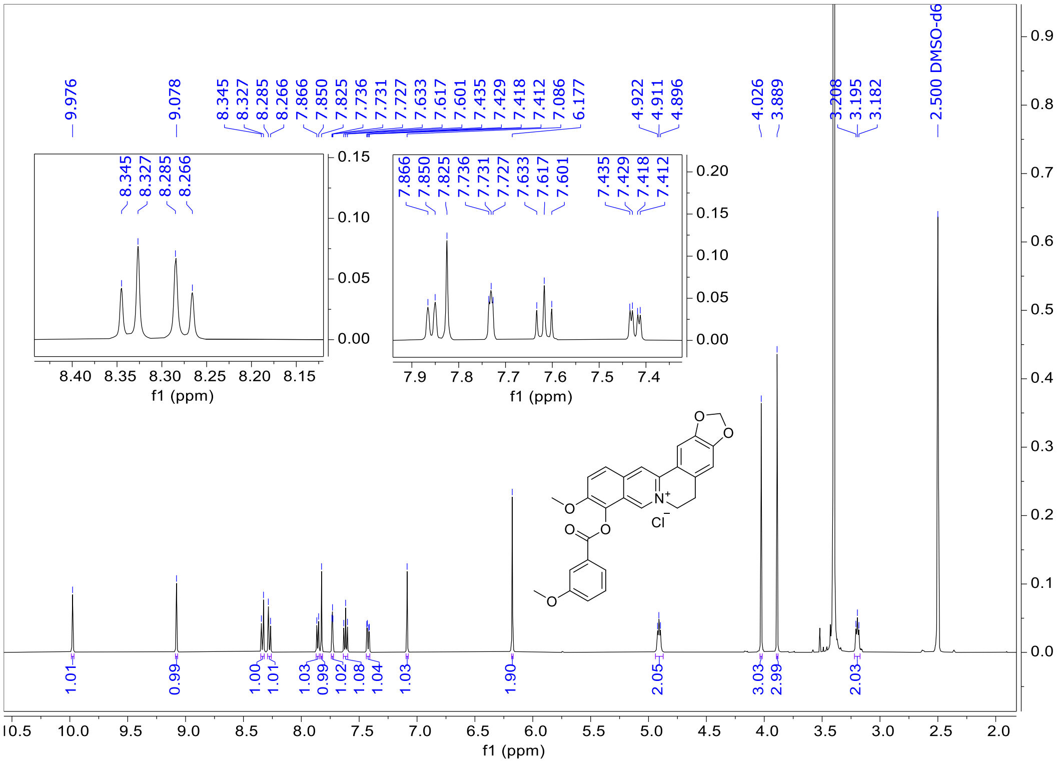

Figure S9: ^{1}H-NMR (500 MHz, DMSO-d_6) spectrum of compound **3**

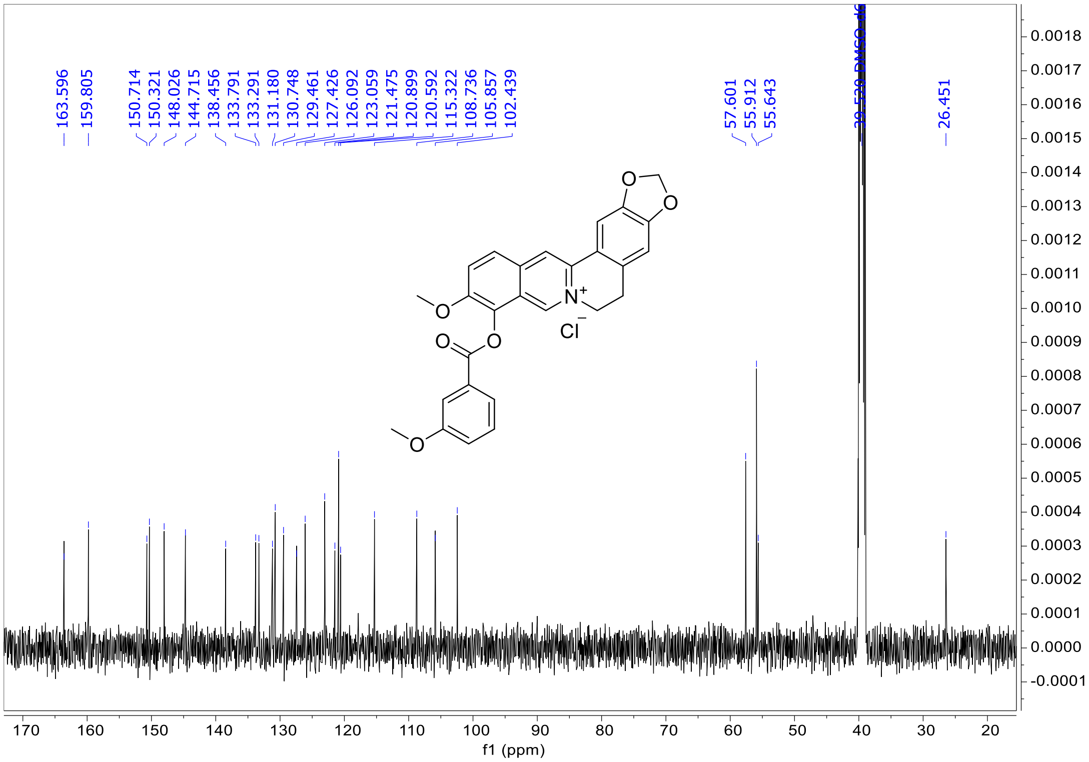

Figure S10: ^{13}C-NMR (125 MHz, DMSO-d_6) spectrum of compound **3**

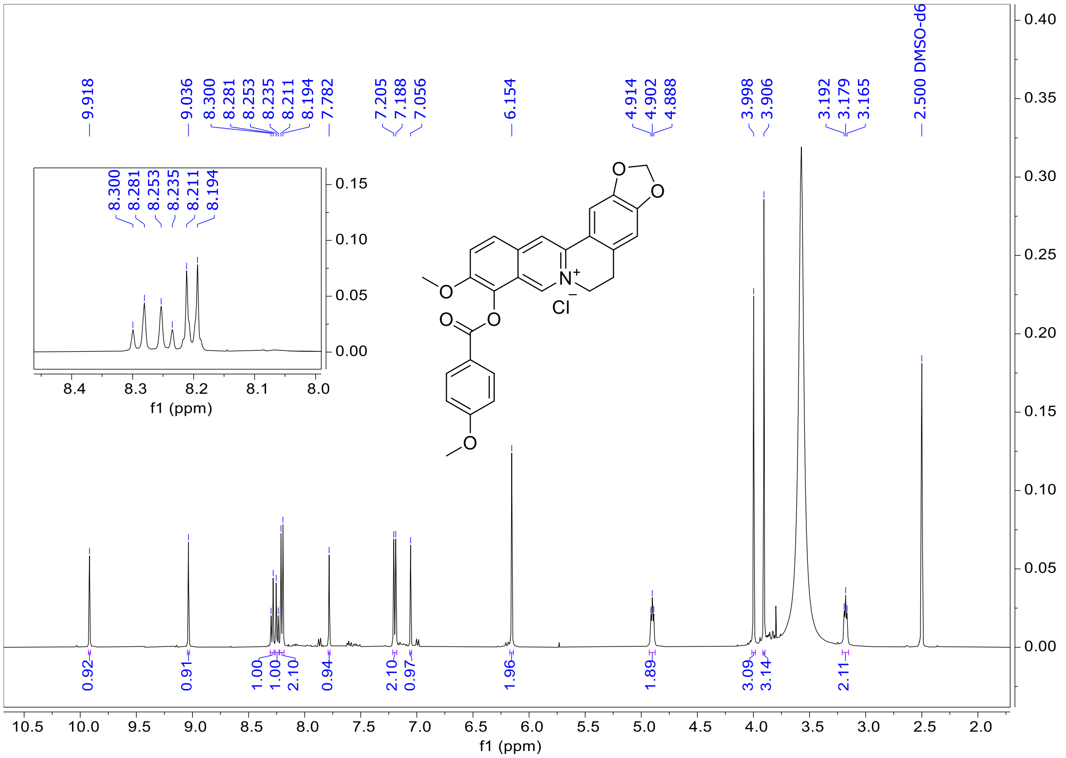

Figure S11: ^{1}H-NMR (500 MHz, DMSO-d_6) spectrum of compound **4**

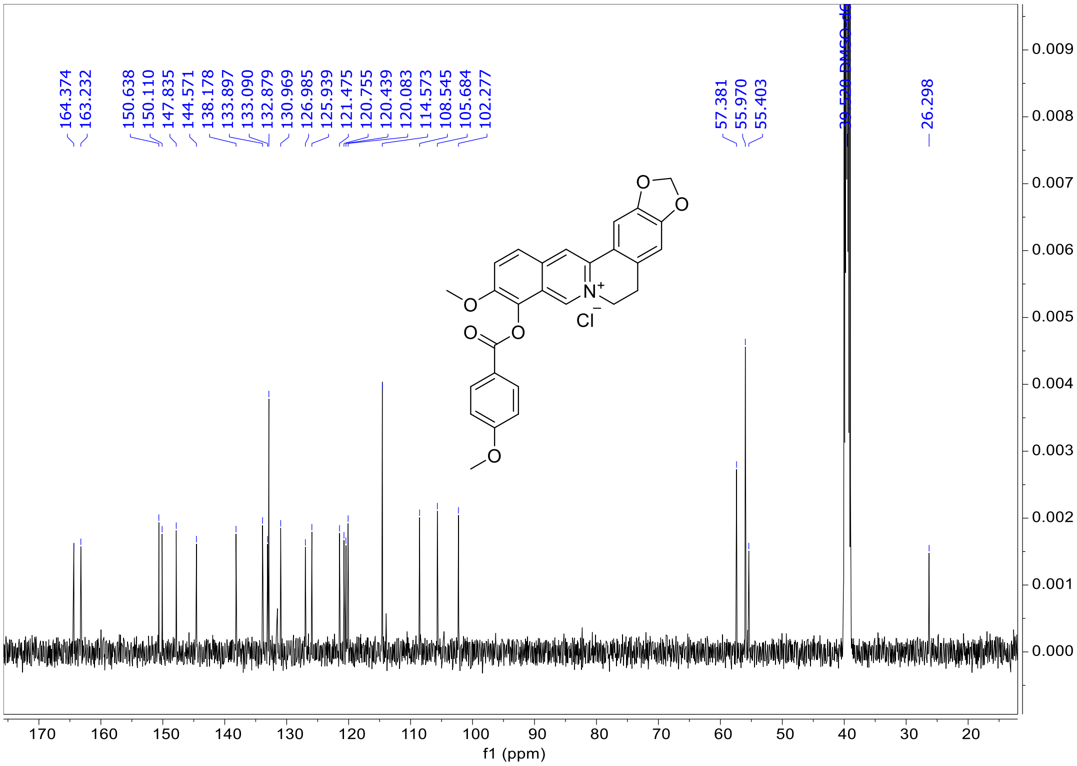

Figure S12: ^{13}C-NMR (125 MHz, DMSO-d_6) spectrum of compound **4**

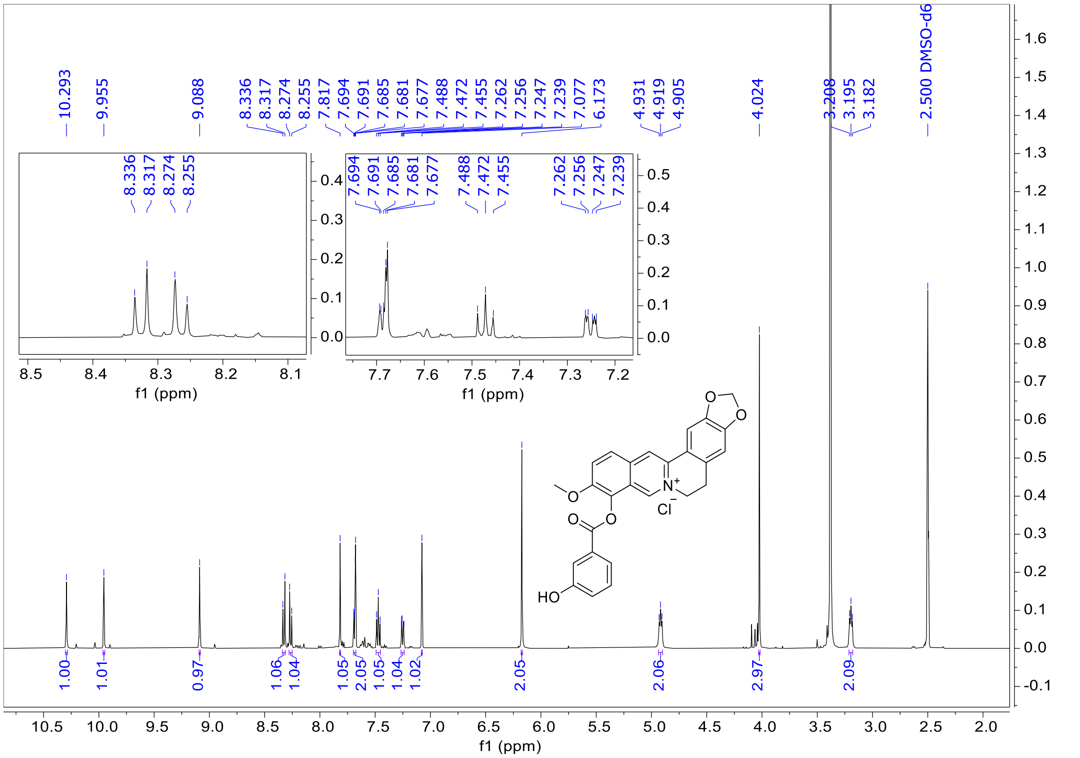

Figure S13: ^{1}H-NMR (500 MHz, DMSO-d_6) spectrum of compound **5**

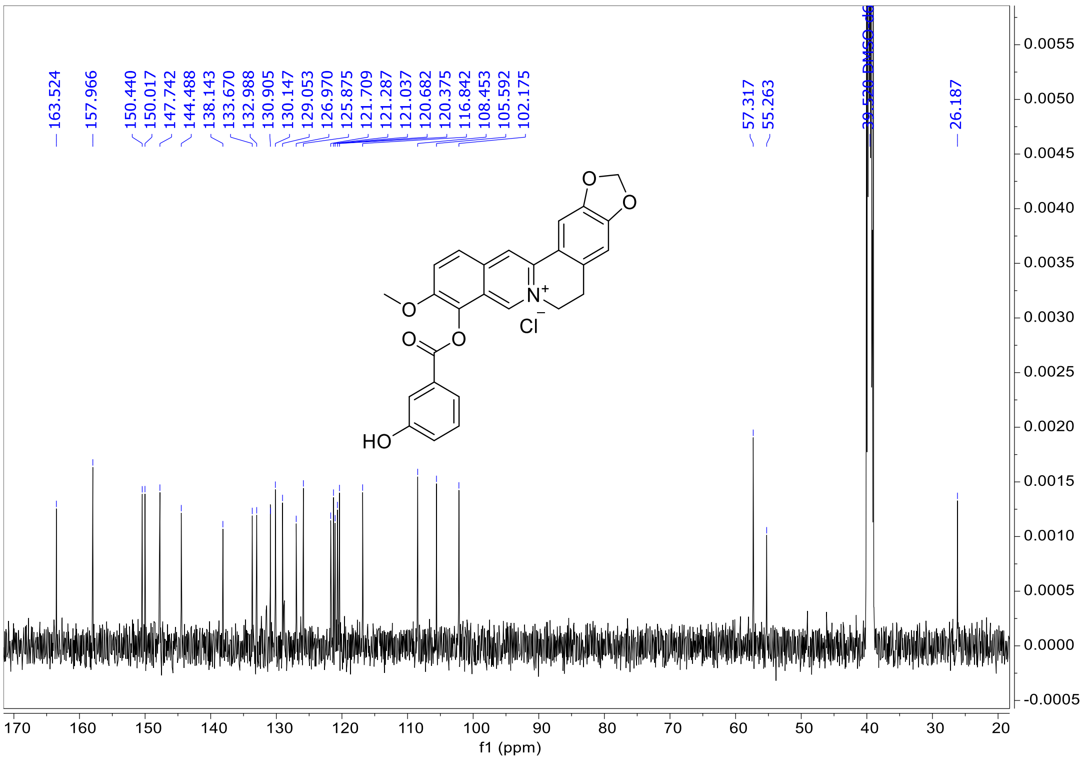

Figure S14: ^{13}C-NMR (125 MHz, DMSO-d_6) spectrum of compound **5**

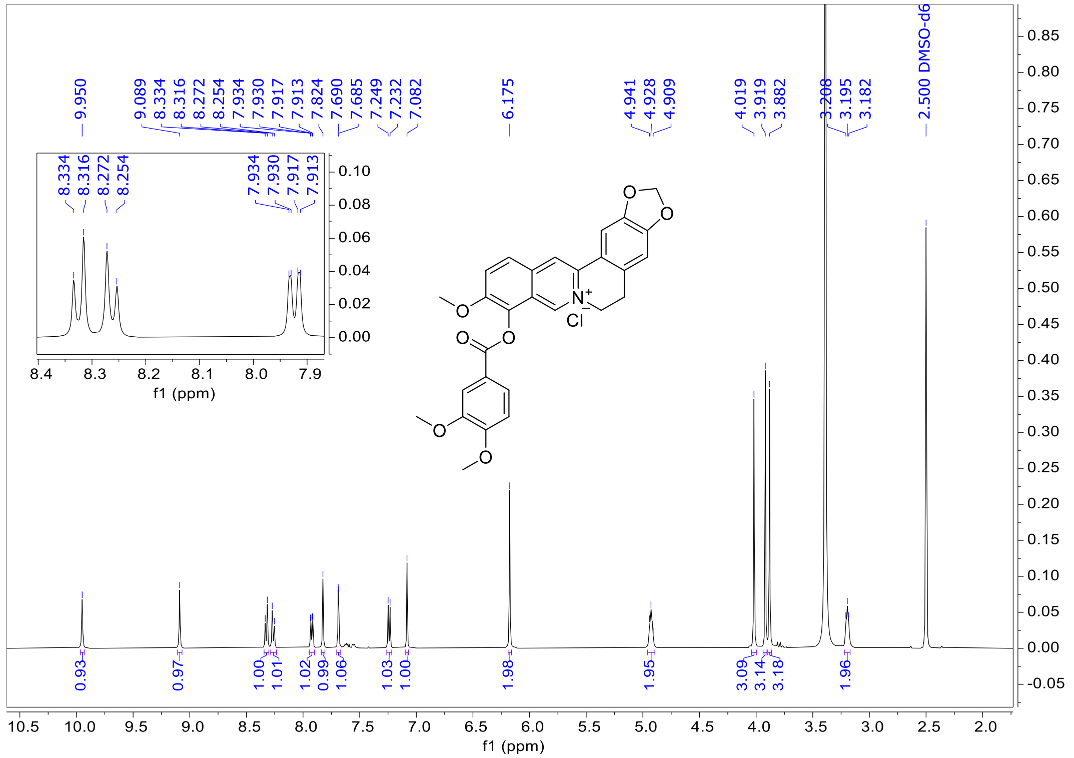

Figure S15: ^{1}H-NMR (500 MHz, DMSO-d_6) spectrum of compound **6**

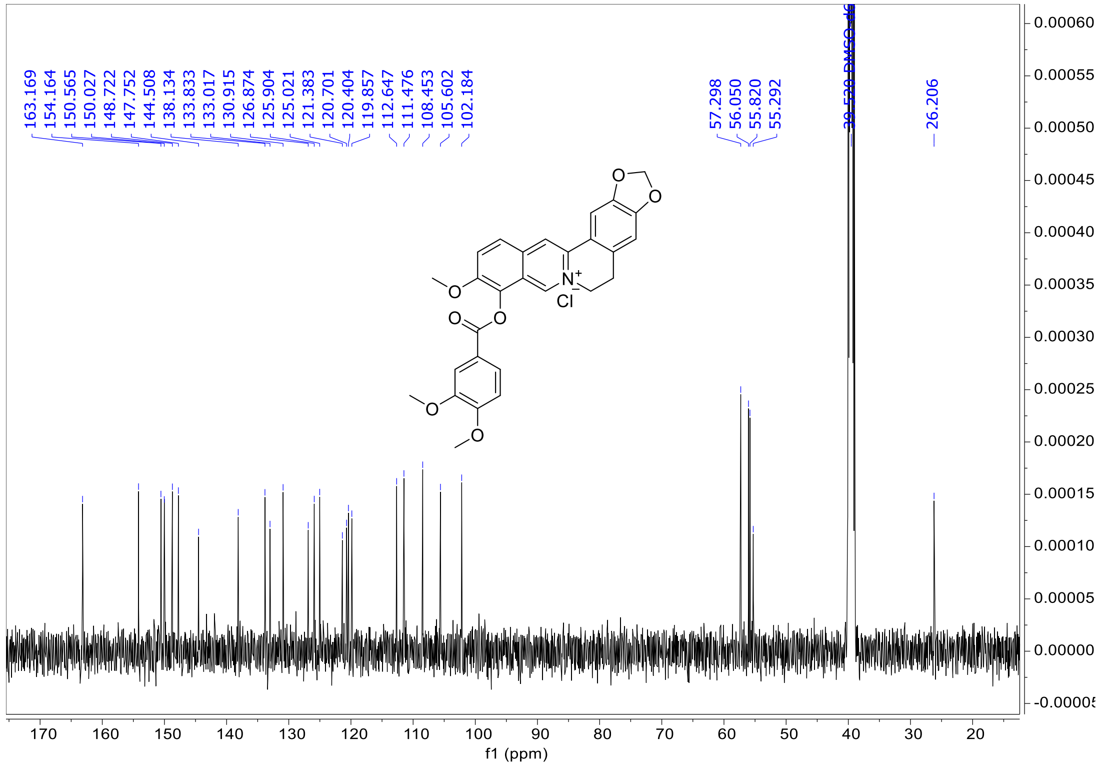

Figure S16: ^{13}C-NMR (125 MHz, DMSO-d_6) spectrum of compound **6**

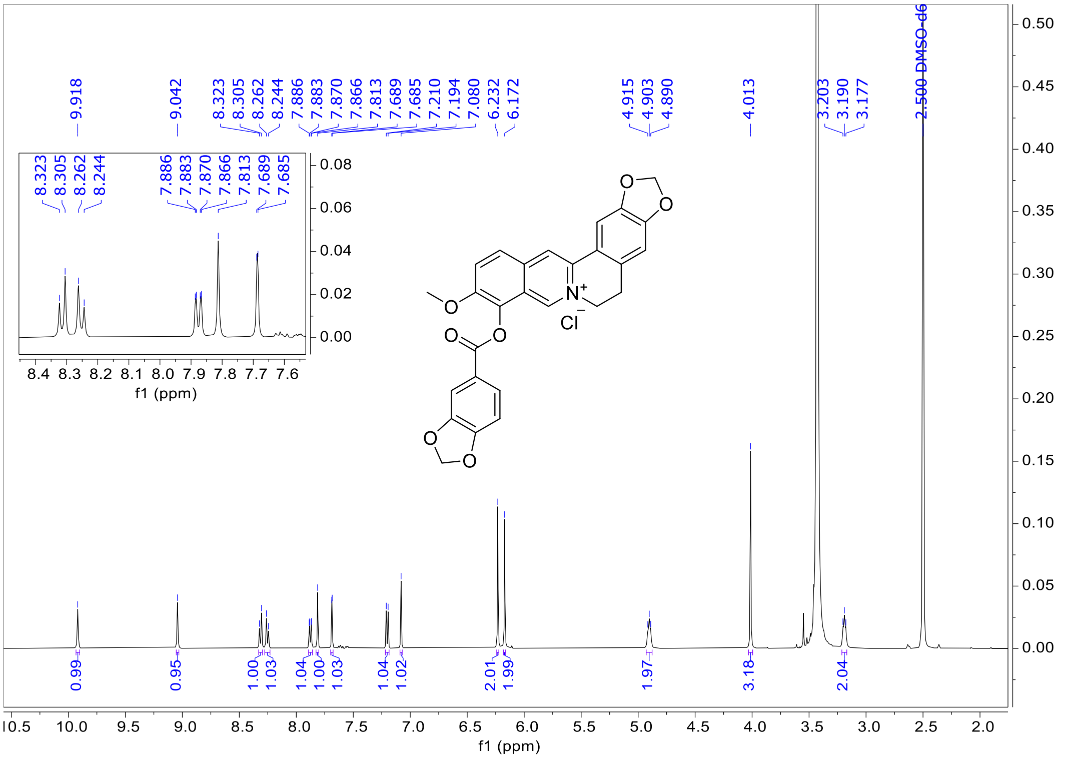

Figure S17: ^{1}H-NMR (500 MHz, DMSO-d_6) spectrum of compound **7**

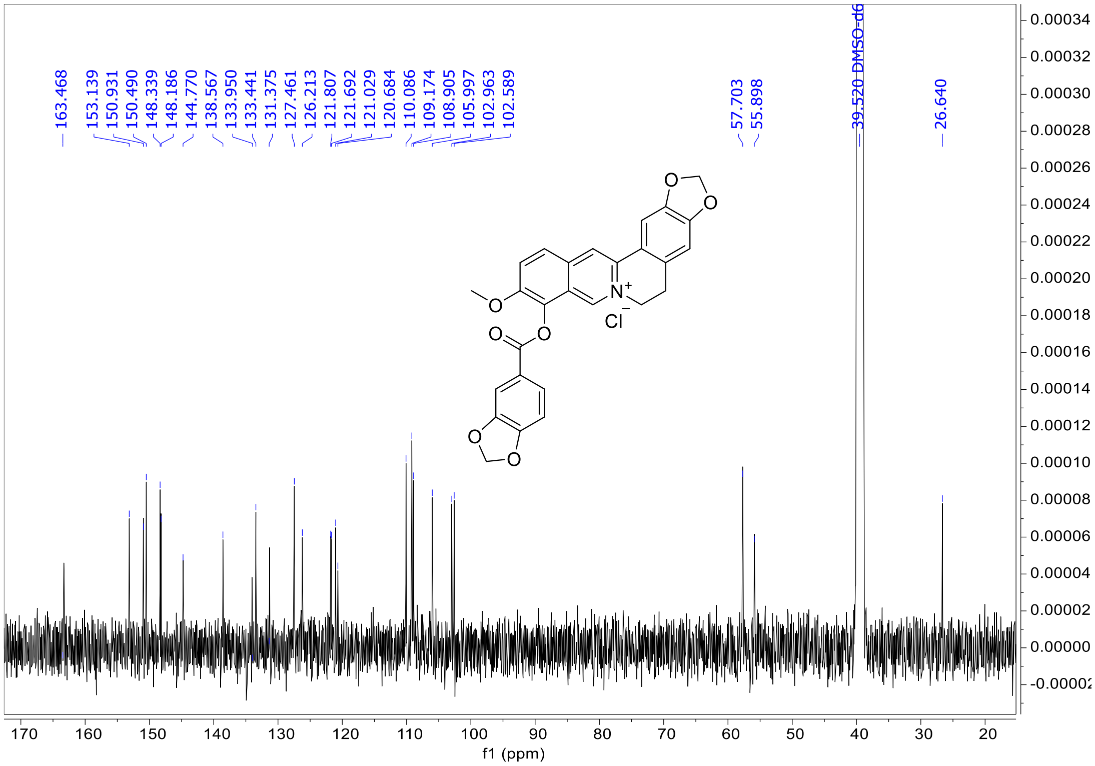

Figure S18: ^{13}C-NMR (125 MHz, DMSO-d_6) spectrum of compound **7**

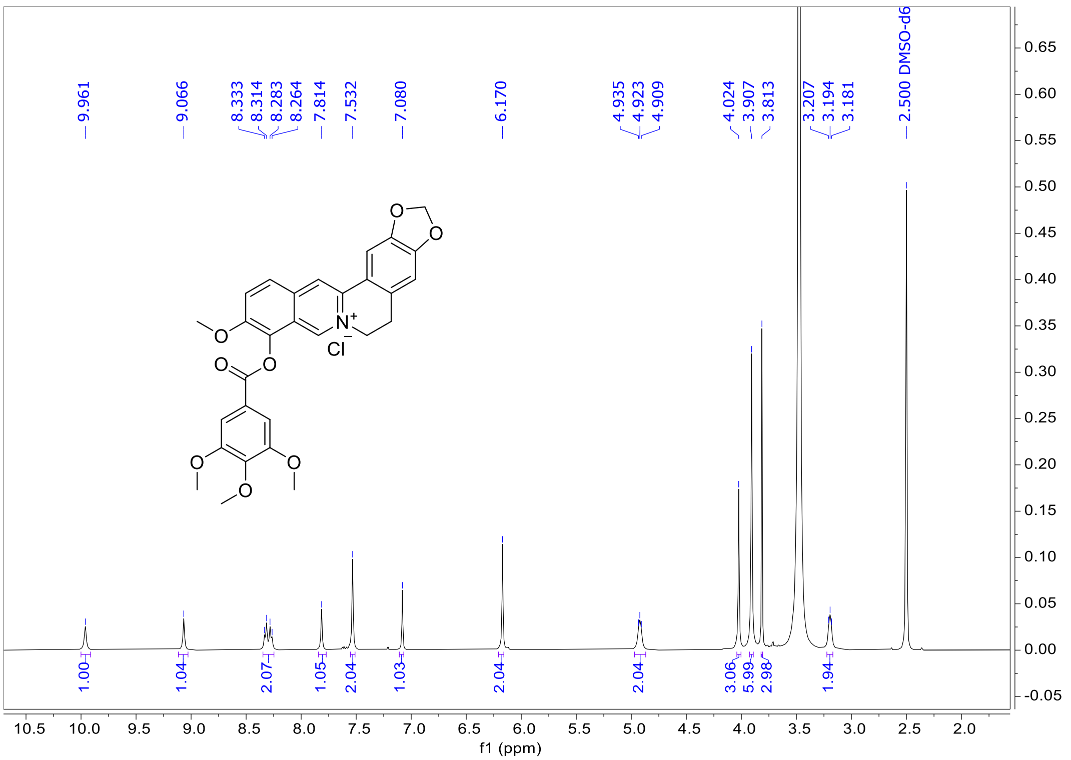

Figure S19: ^{1}H-NMR (500 MHz, DMSO-d_6) spectrum of compound **8**

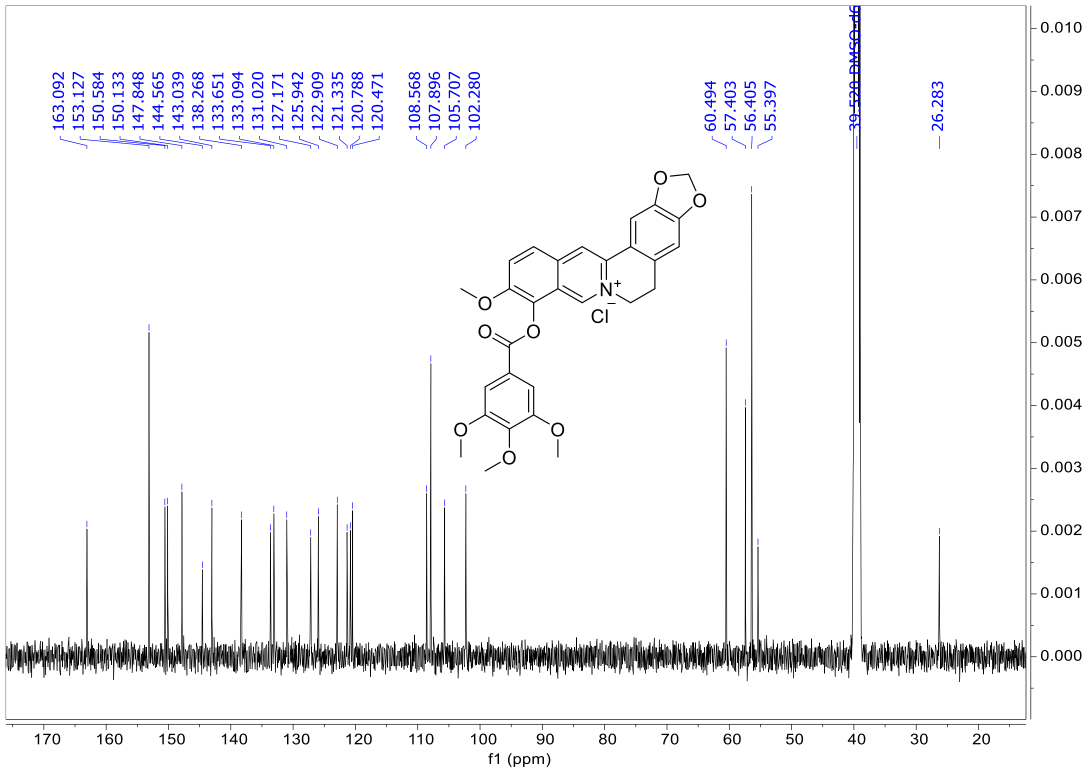

Figure S20: ^{13}C-NMR (125 MHz, DMSO-d_6) spectrum of compound **8**

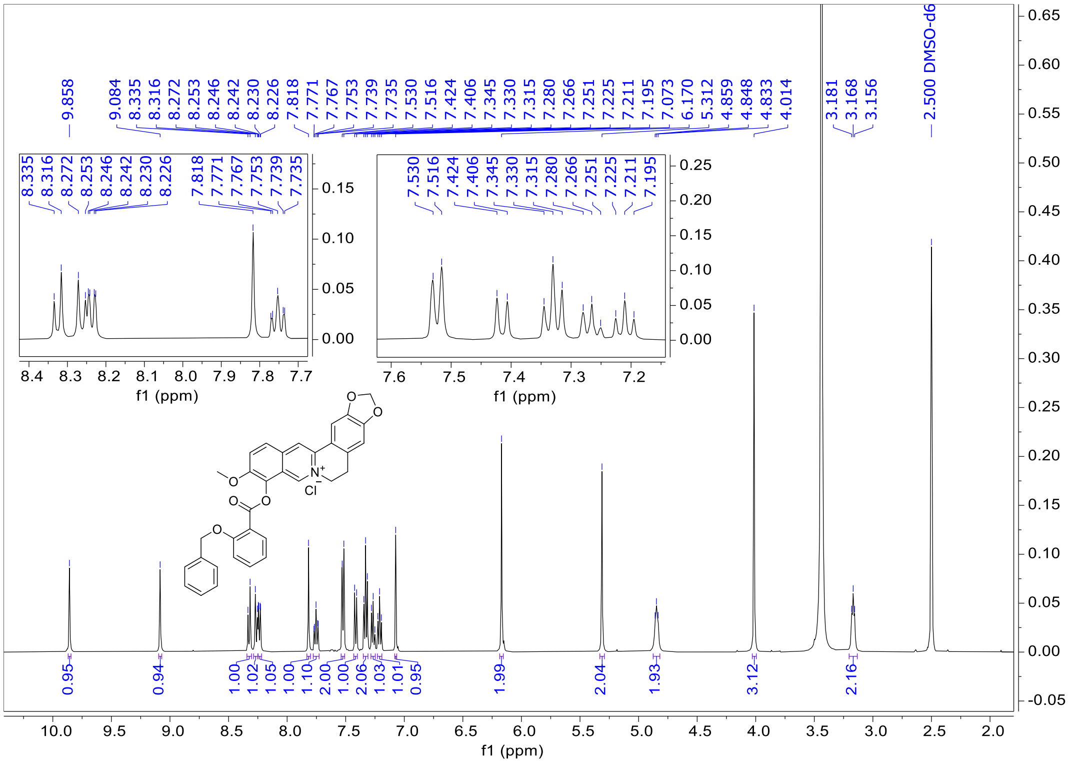

Figure S21: ^{1}H-NMR (500 MHz, DMSO-d_6) spectrum of compound **9**

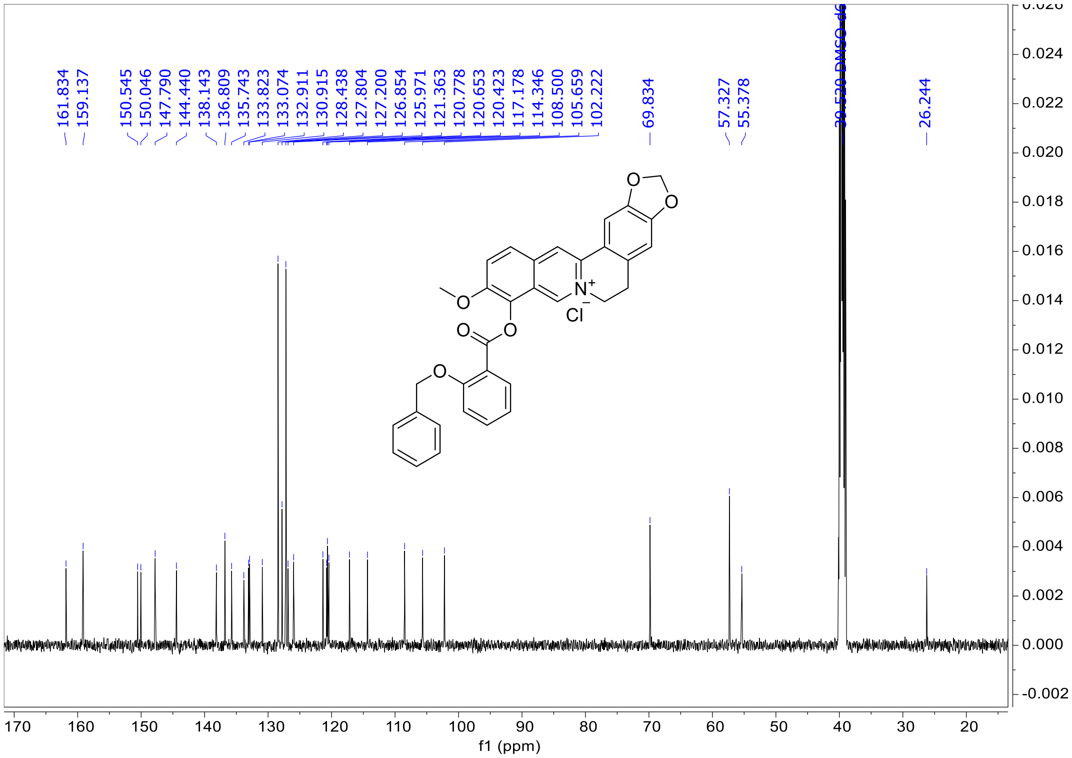

Figure S22: ^{13}C-NMR (125 MHz, DMSO-d_6) spectrum of compound **9**

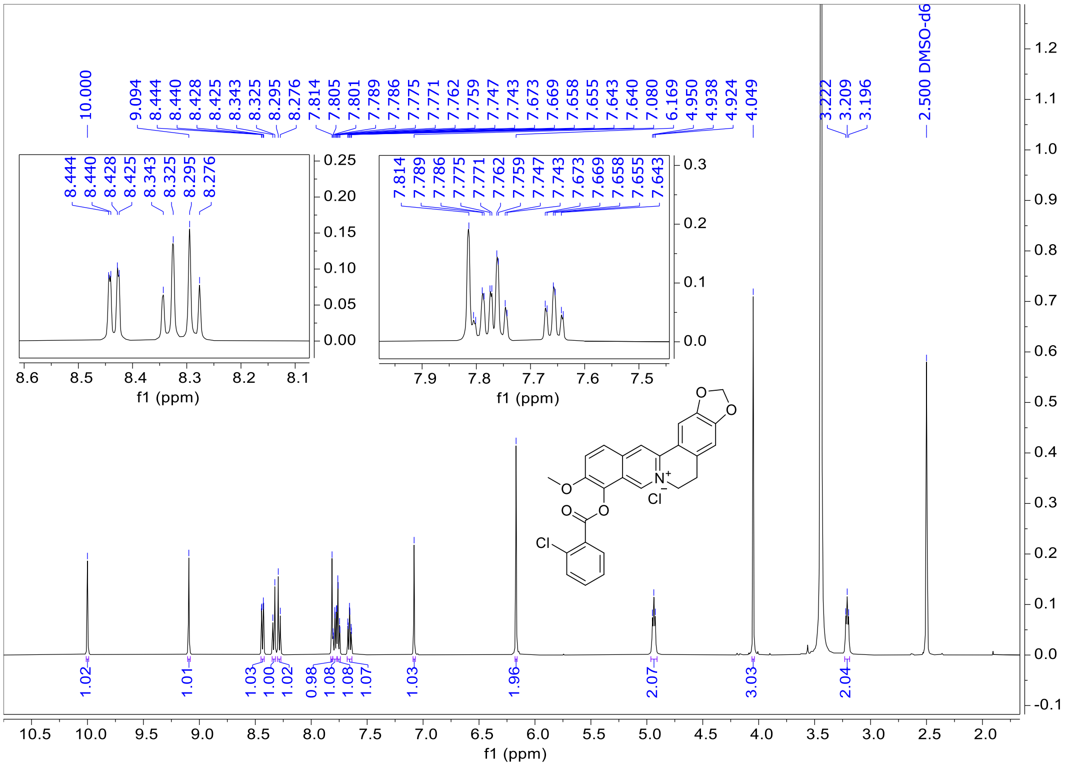

Figure S23: ^{1}H-NMR (500 MHz, DMSO-d_6) spectrum of compound **10**

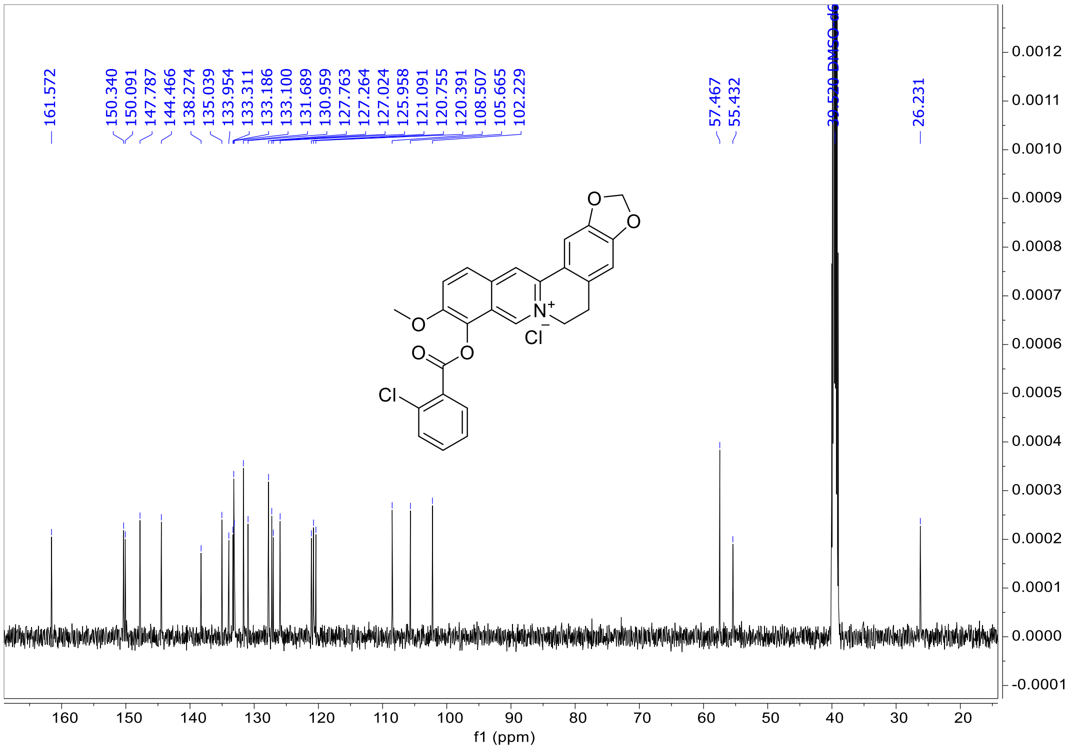

Figure S24: ^{13}C-NMR (125 MHz, DMSO-d_6) spectrum of compound **10**

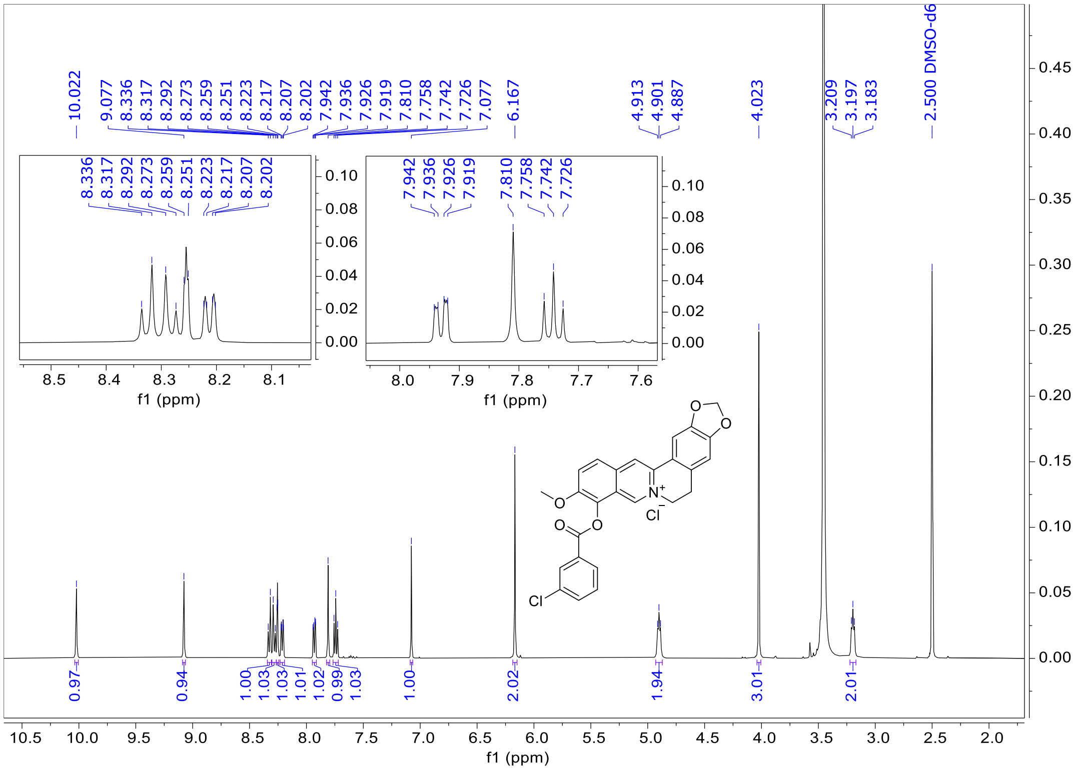

Figure S25: ^{1}H-NMR (500 MHz, DMSO-d_6) spectrum of compound **11**

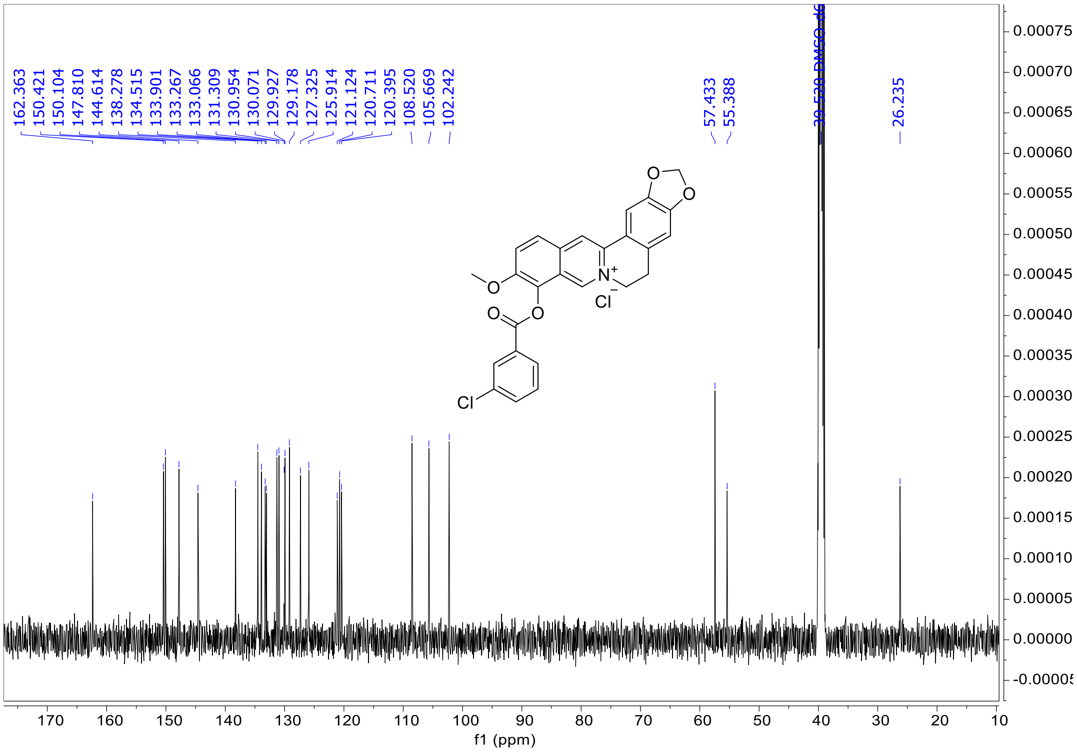

Figure S26: ^{13}C-NMR (125 MHz, DMSO-d_6) spectrum of compound **11**

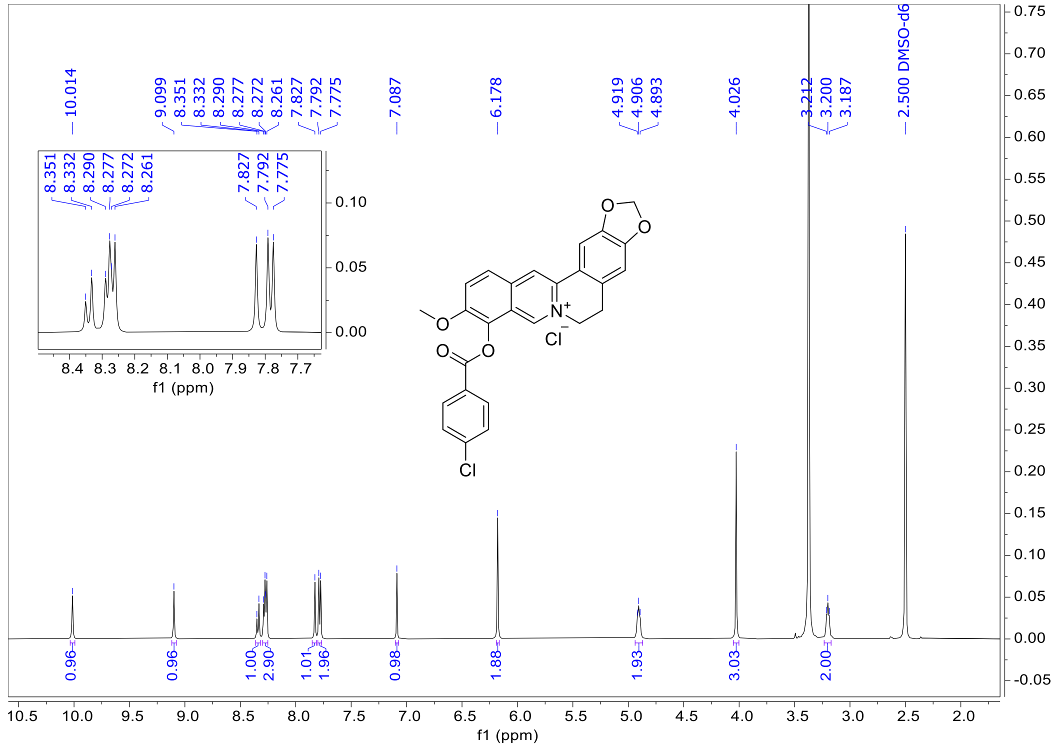

Figure S27: ^{1}H-NMR (500 MHz, DMSO-d_6) spectrum of compound **12**

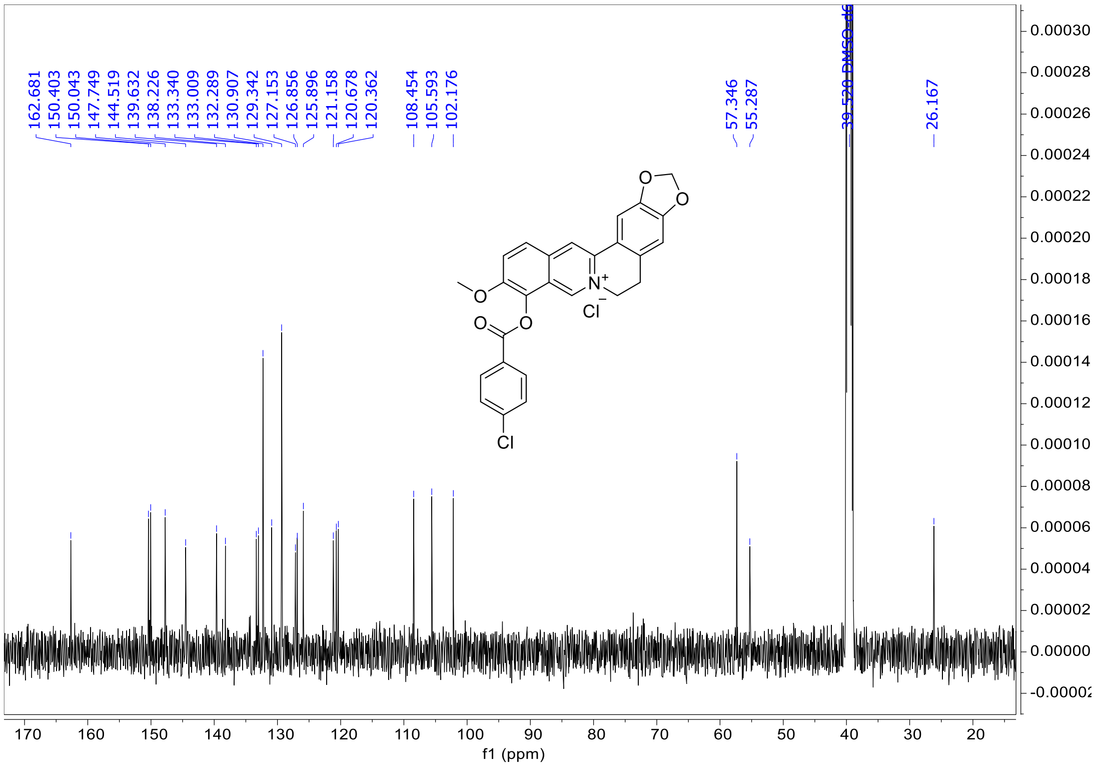

Figure S28: ^{13}C-NMR (125 MHz, DMSO-d_6) spectrum of compound **12**

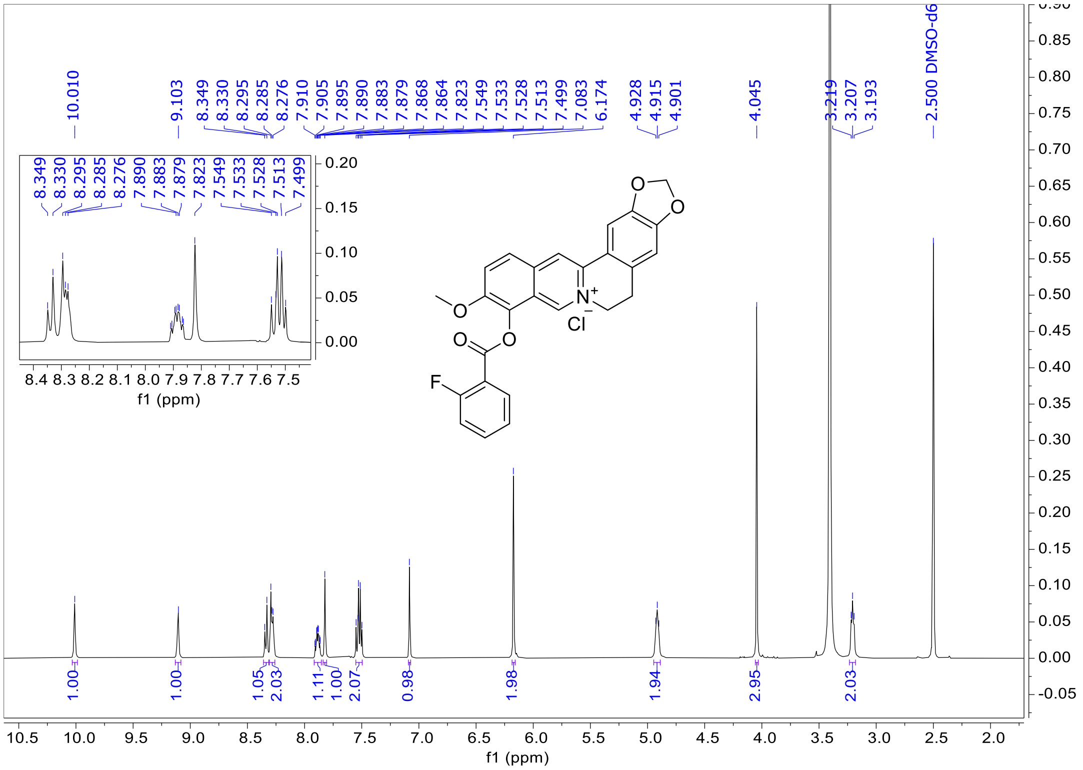

Figure S29: ^{1}H-NMR (500 MHz, DMSO-d_6) spectrum of compound **13**

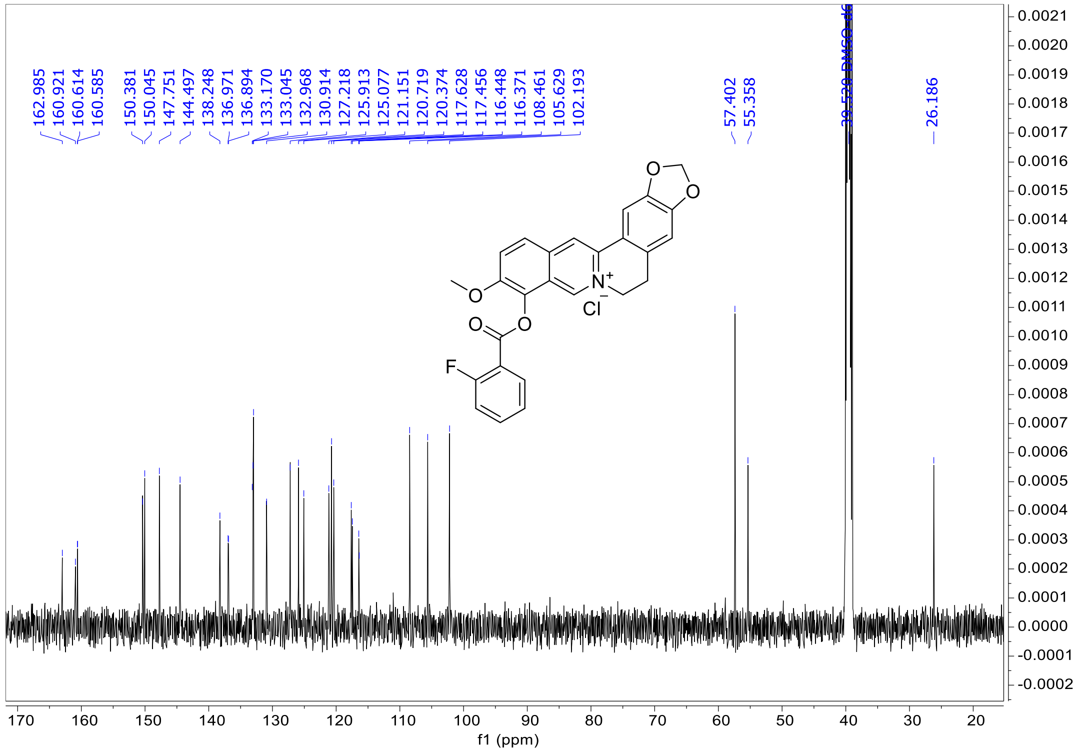

Figure S30: ^{13}C-NMR (125 MHz, DMSO-d_6) spectrum of compound **13**

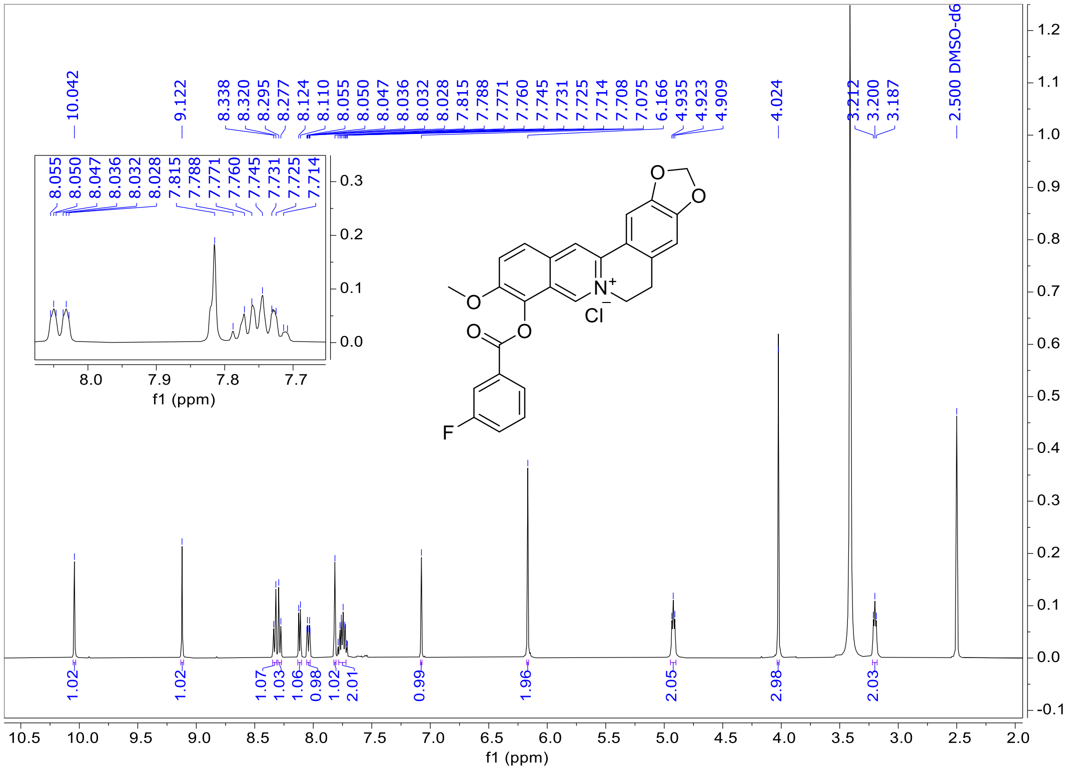

Figure S31: ^{1}H-NMR (500 MHz, DMSO-d_6) spectrum of compound **14**

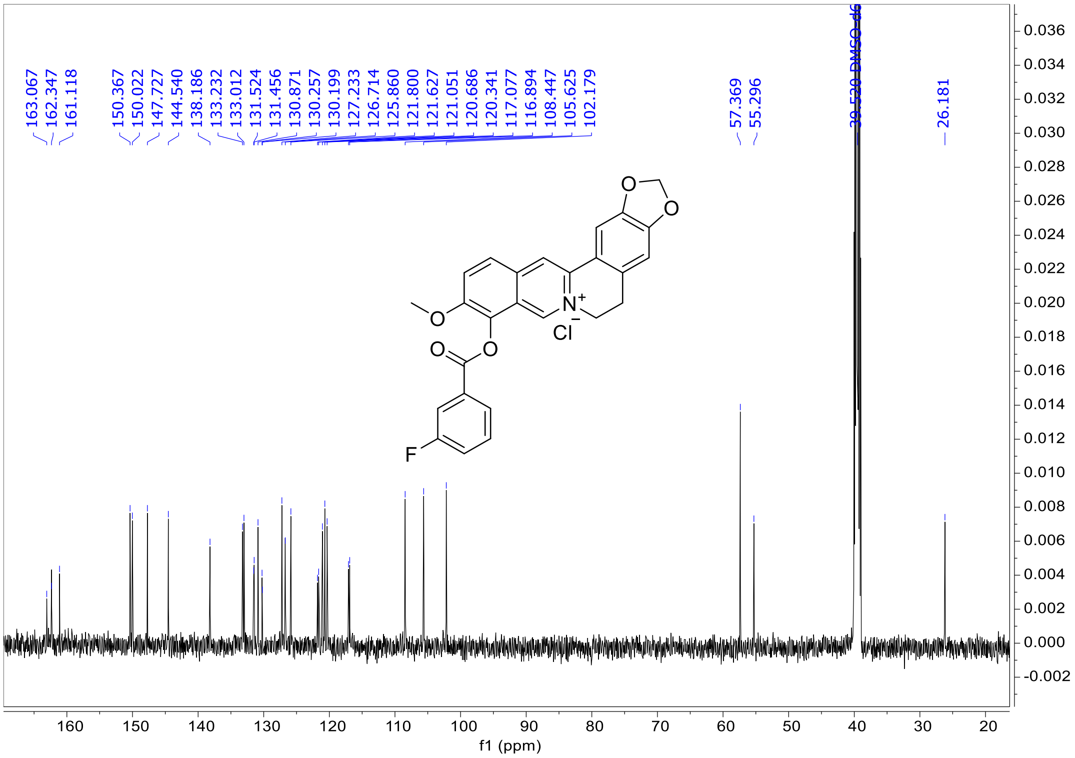

Figure S32: ^{13}C-NMR (125 MHz, DMSO-d_6) spectrum of compound **14**

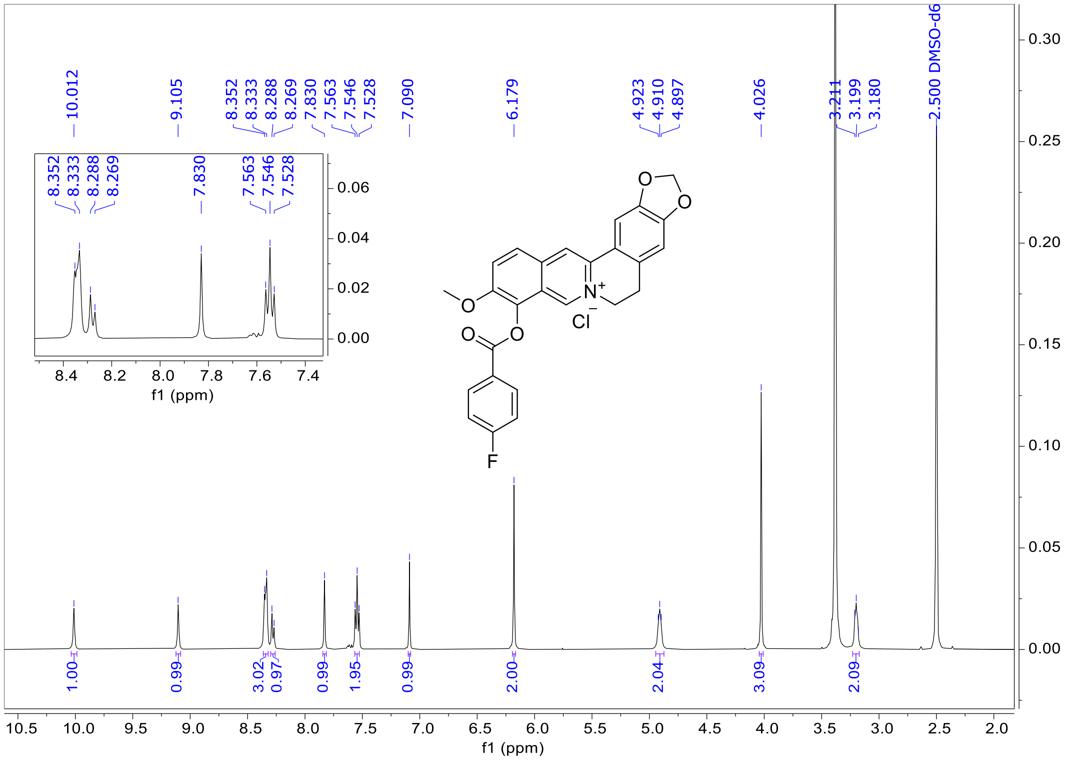

Figure S33: ^{1}H-NMR (500 MHz, DMSO-d_6) spectrum of compound **15**

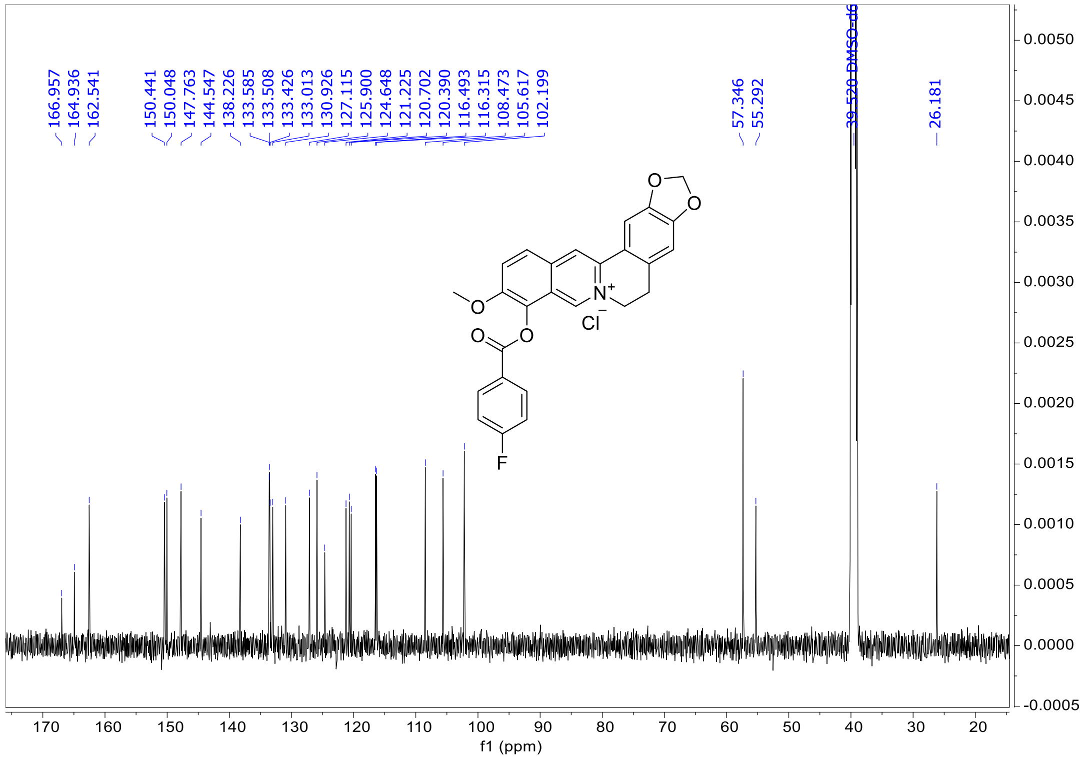

Figure S34: ^{13}C-NMR (125 MHz, DMSO-d_6) spectrum of compound **15**

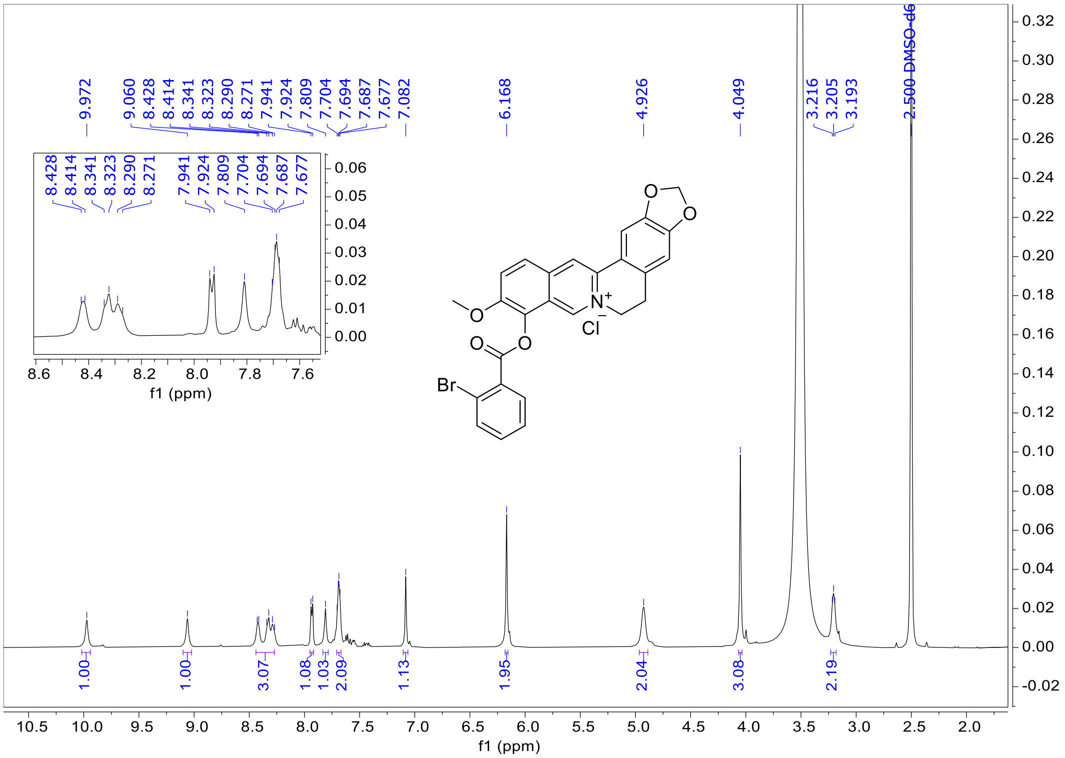

Figure S35: ¹H-NMR (500 MHz, DMSO-d_6) spectrum of compound **16**

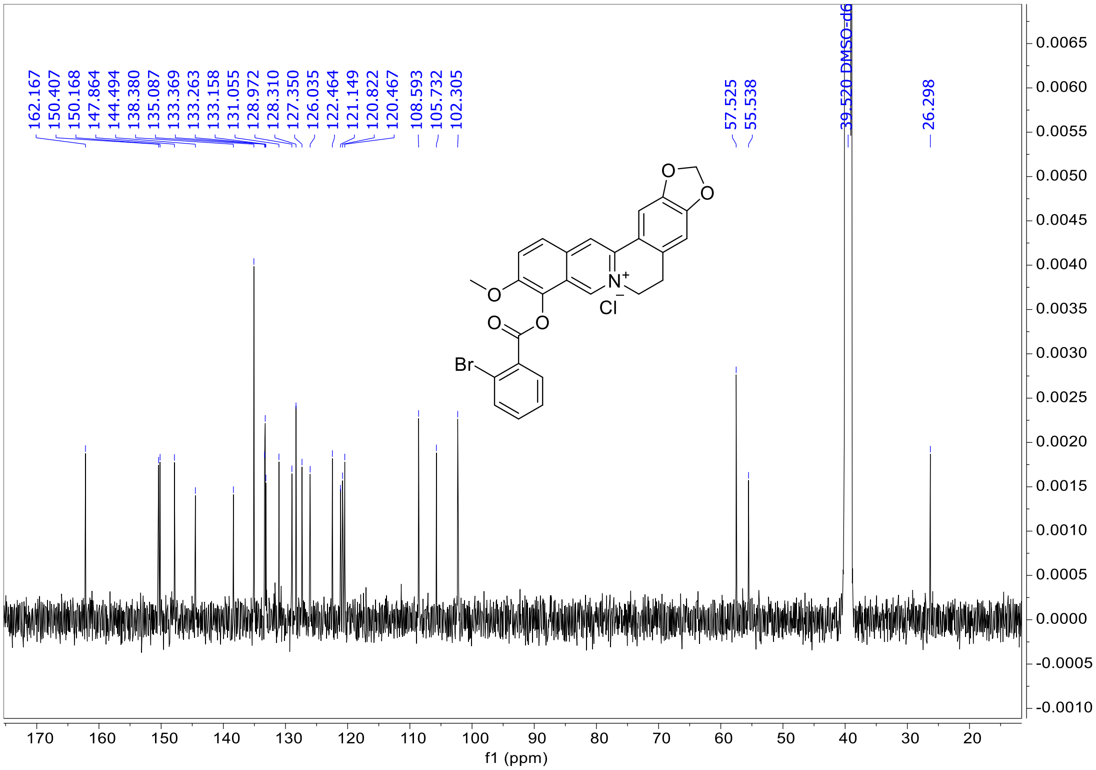

Figure S36: ¹³C-NMR (125 MHz, DMSO-d_6) spectrum of compound **16**

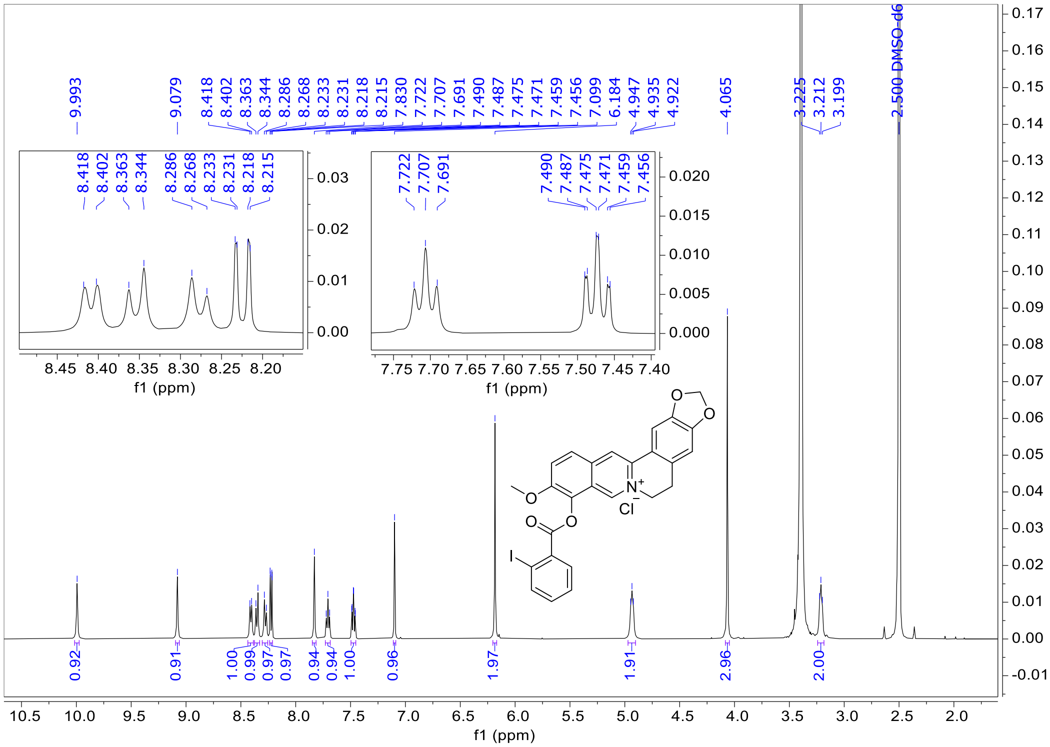

Figure S37: ^{1}H-NMR (500 MHz, DMSO-d_6) spectrum of compound **17**

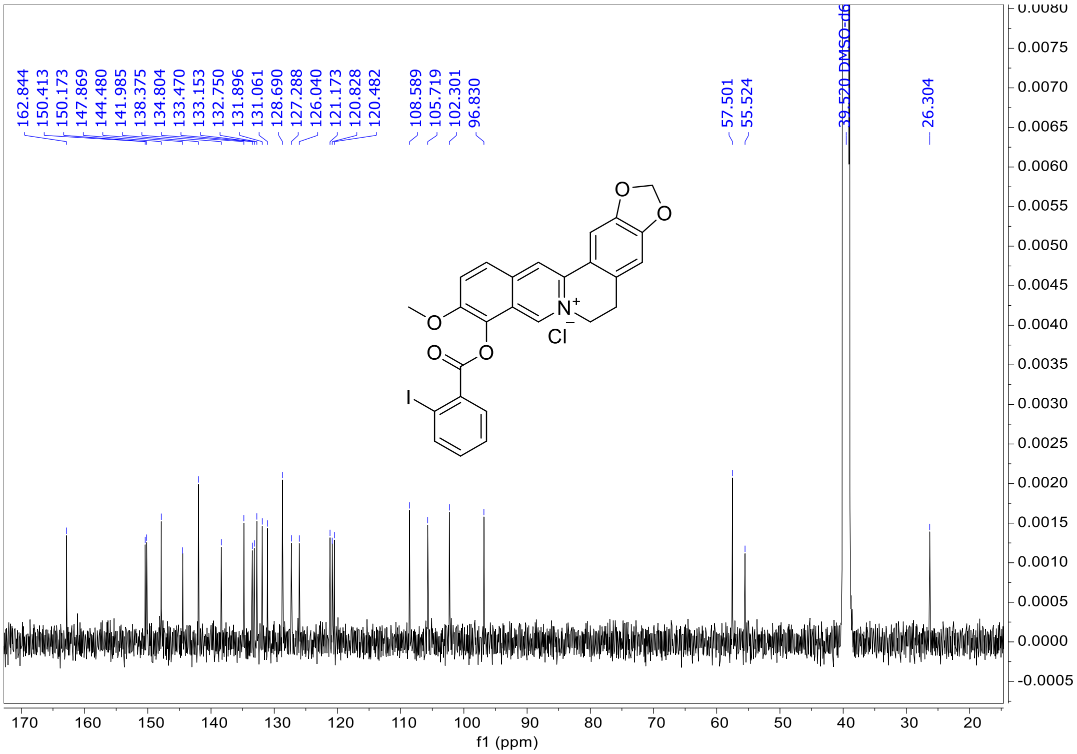

Figure S38: ^{13}C-NMR (125 MHz, DMSO-d_6) spectrum of compound **17**

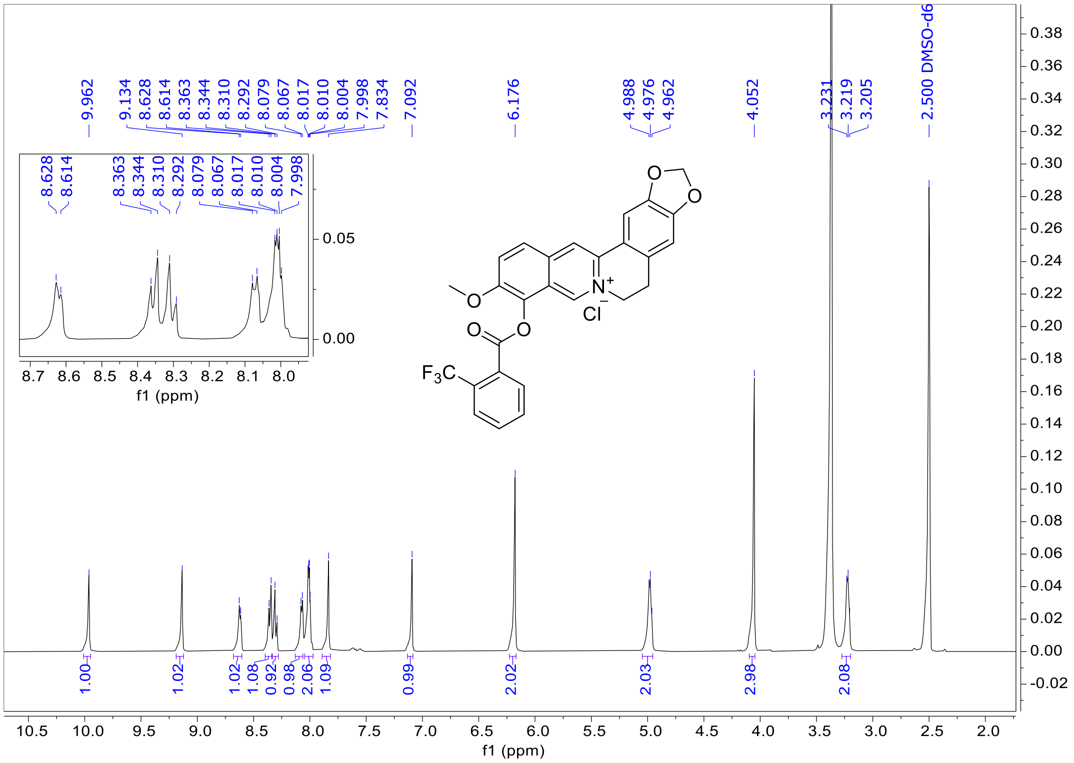

Figure S39: ^{1}H-NMR (500 MHz, DMSO-d_6) spectrum of compound **18**

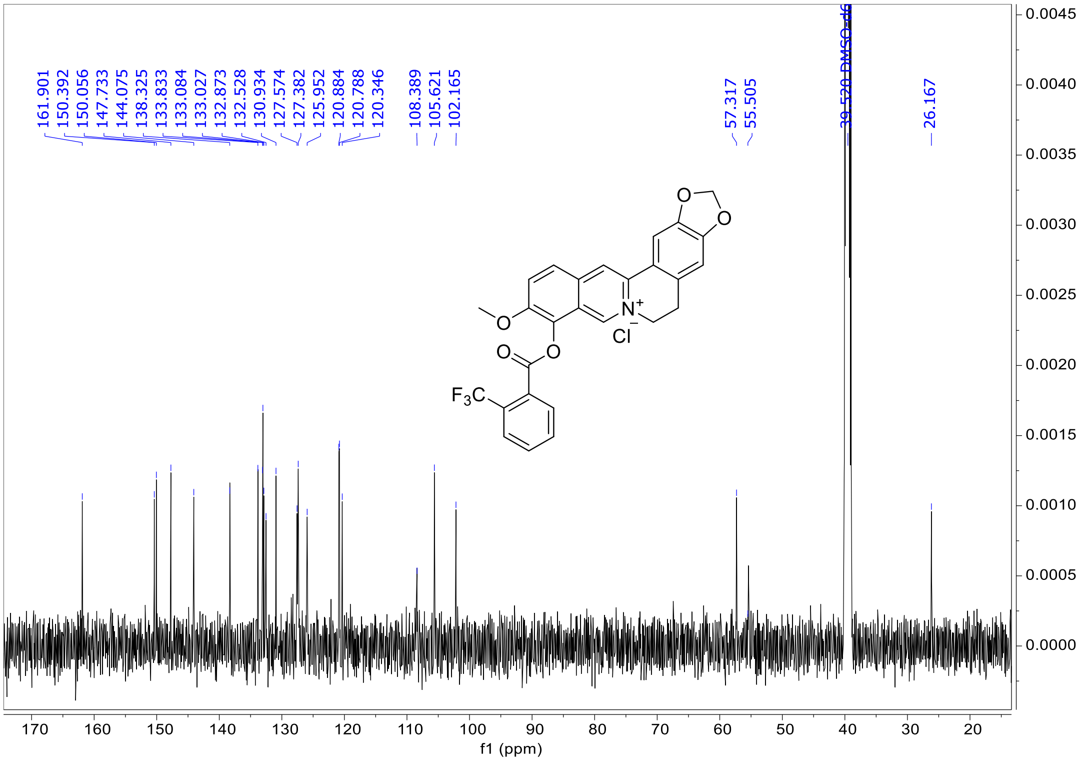

Figure S40: ^{13}C-NMR (125 MHz, DMSO-d_6) spectrum of compound **18**

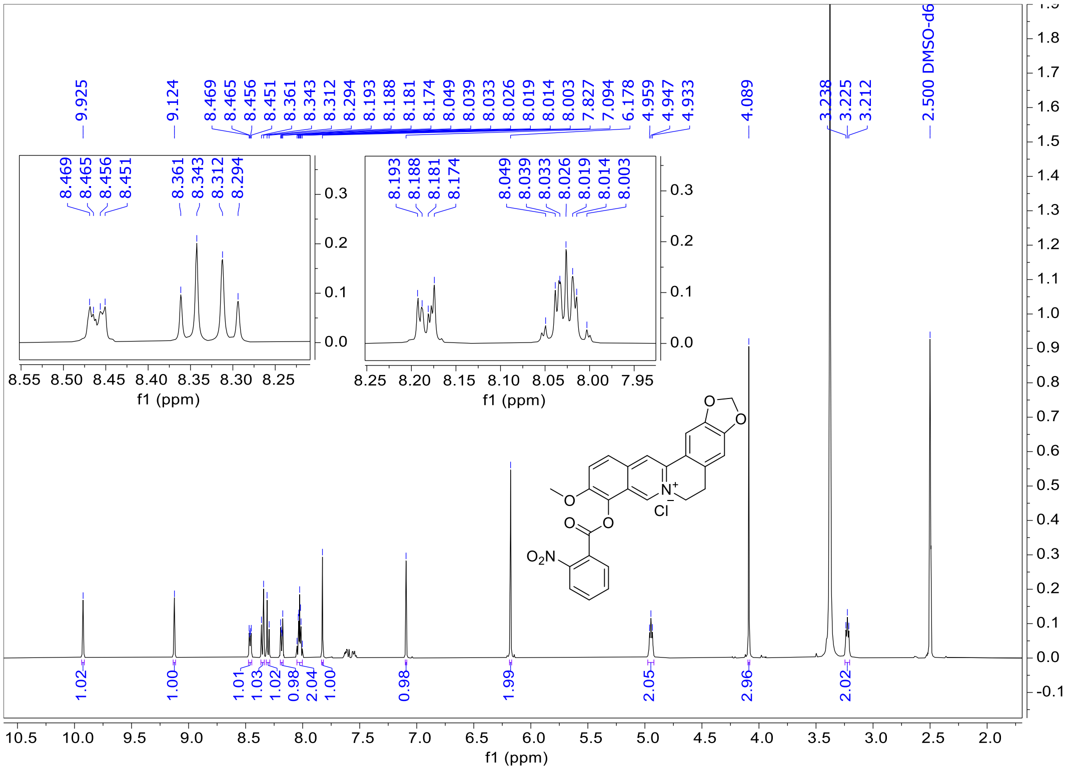

Figure S41: ^{1}H-NMR (500 MHz, DMSO-d_6) spectrum of compound **19**

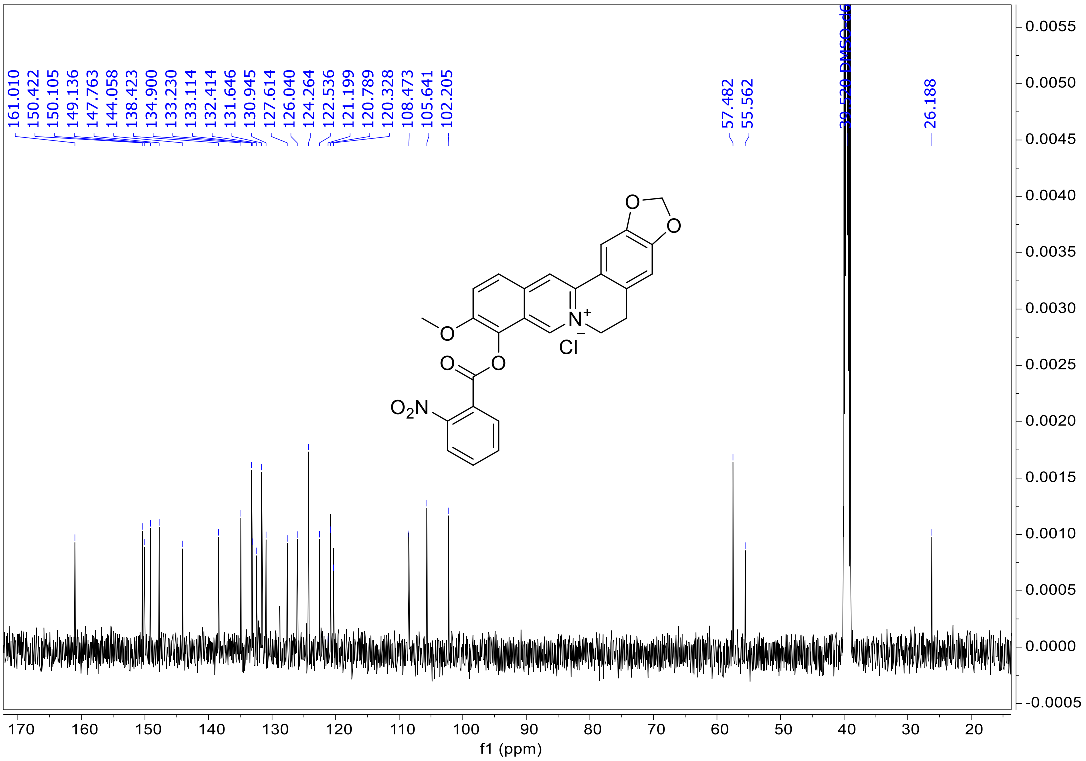

Figure S42: ^{13}C-NMR (125 MHz, DMSO-d_6) spectrum of compound **19**

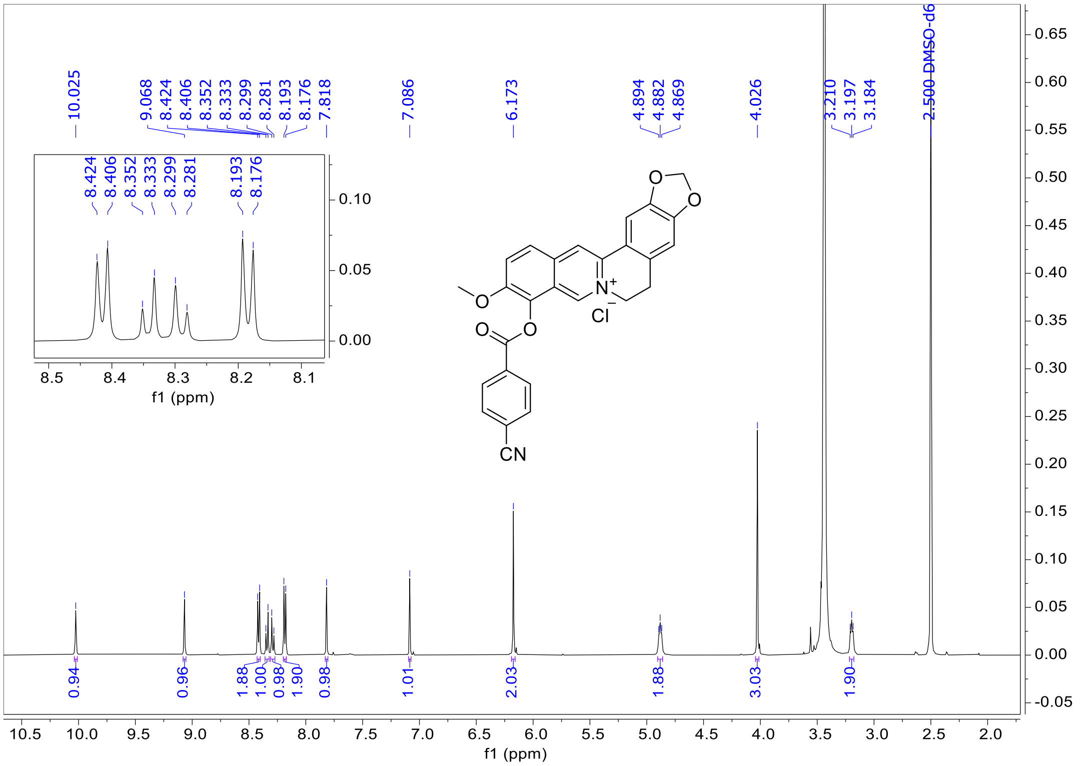

Figure S43: ^{1}H-NMR (500 MHz, DMSO-d_6) spectrum of compound **20**

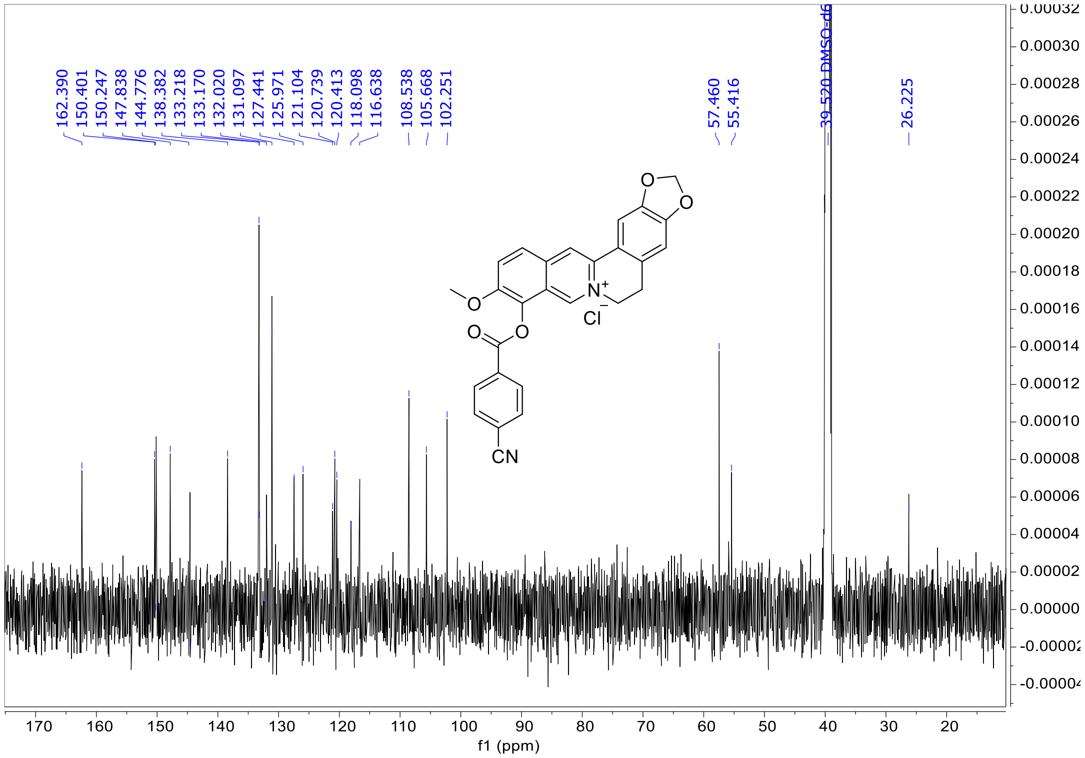

Figure S44: ^{13}C-NMR (125 MHz, DMSO-d_6) spectrum of compound **20**

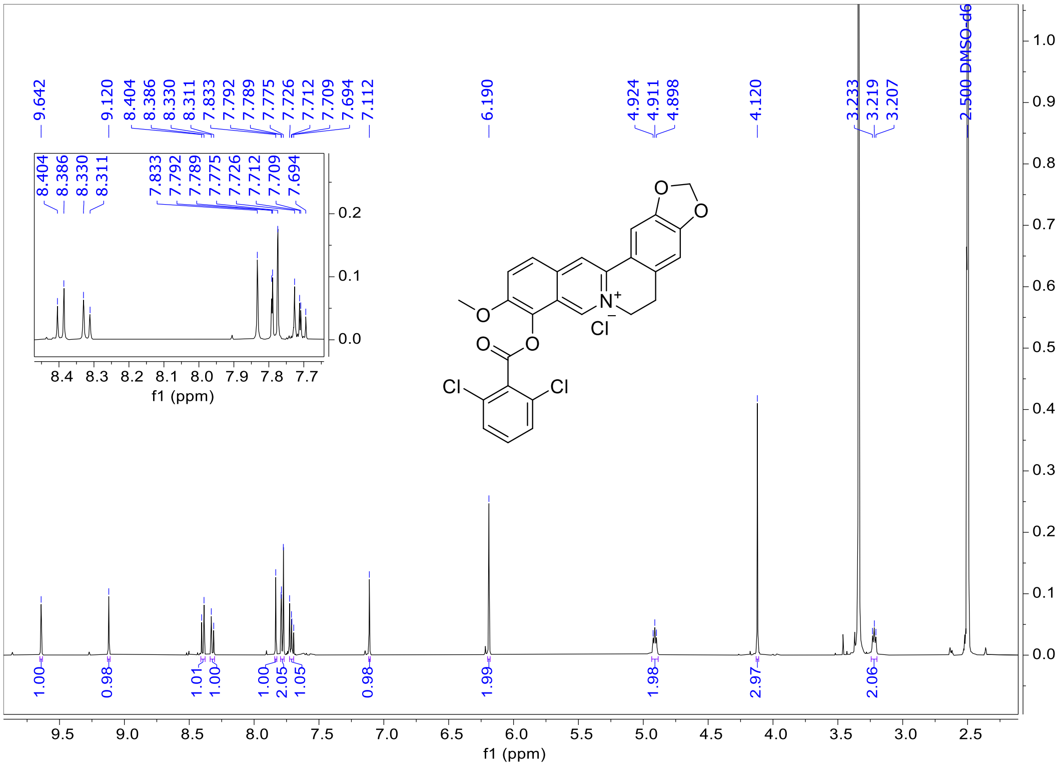

Figure S45: ^{1}H-NMR (500 MHz, DMSO-d_6) spectrum of compound **21**

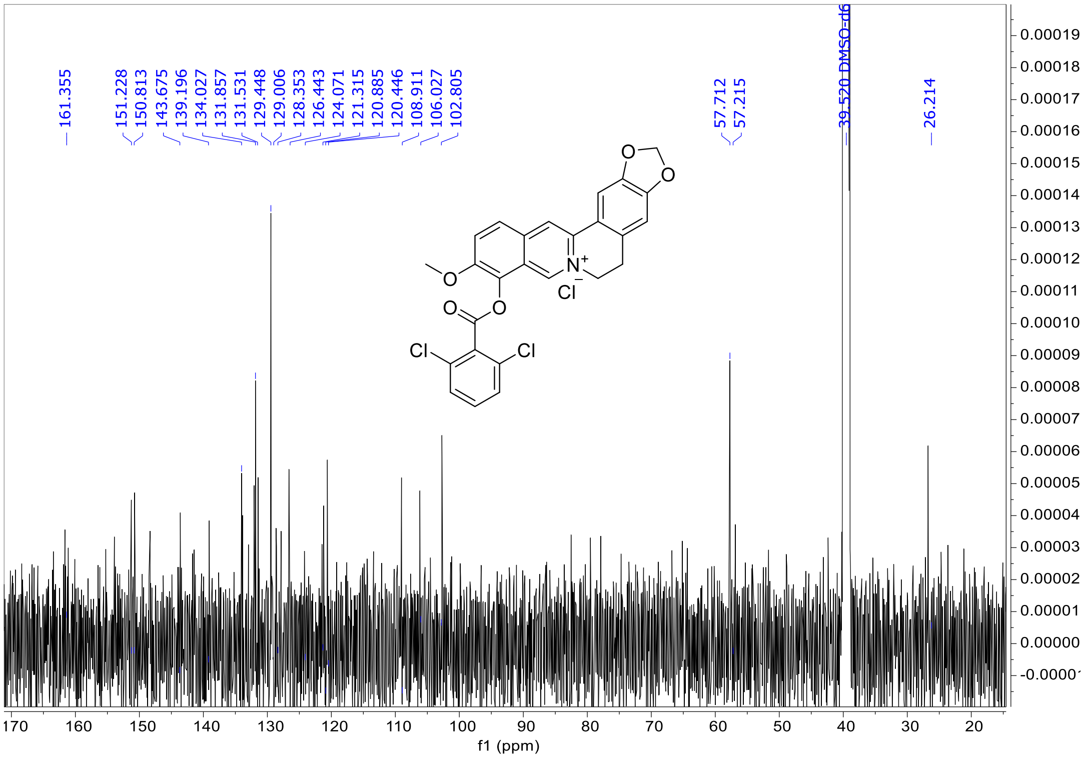

Figure S46: ^{13}C-NMR (125 MHz, DMSO-d_6) spectrum of compound **21**

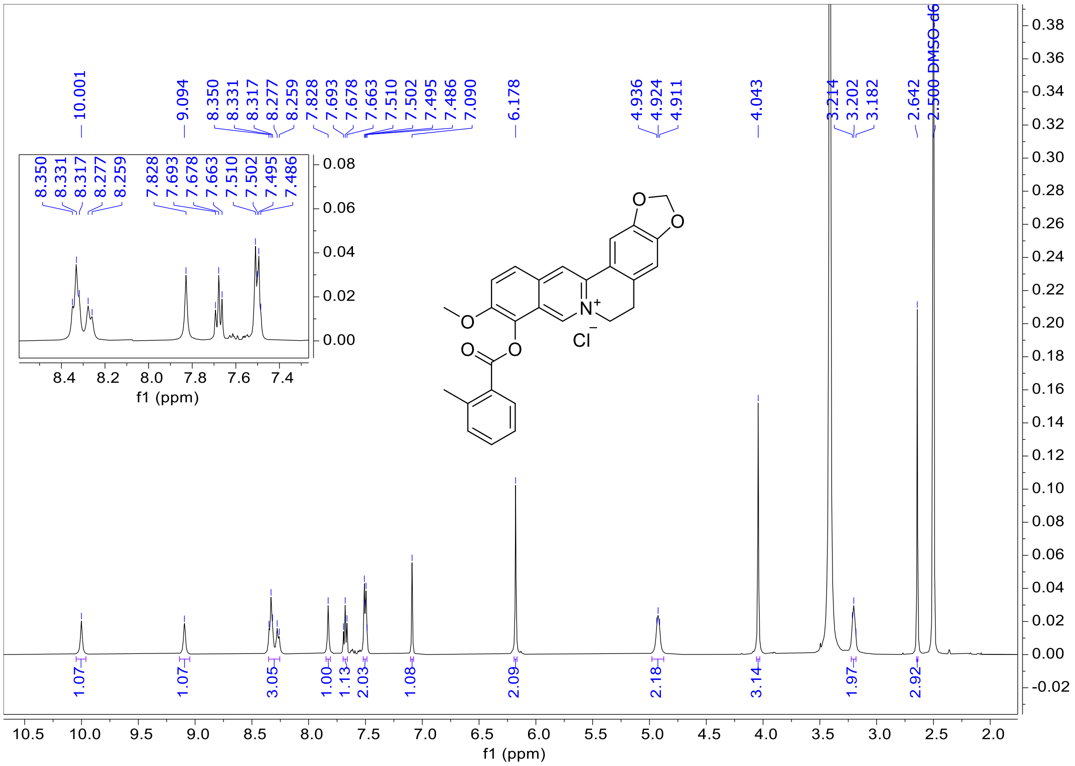

Figure S47: ^{1}H-NMR (500 MHz, DMSO-d_6) spectrum of compound **22**

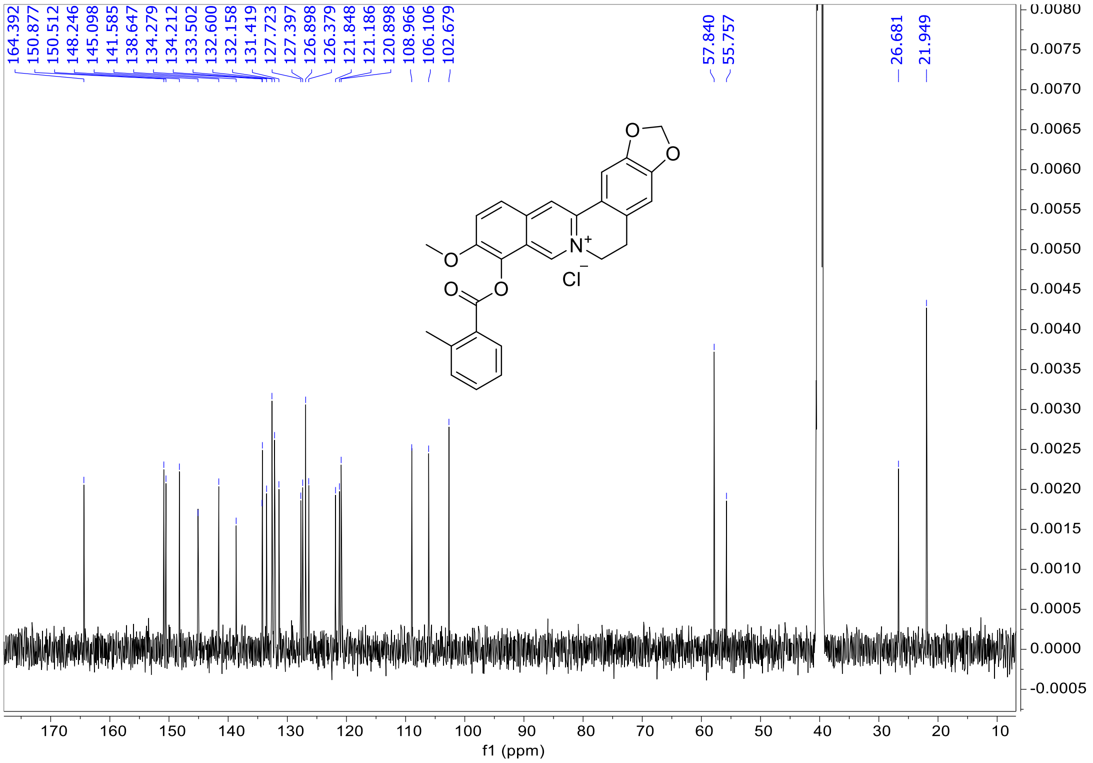

Figure S48: ^{13}C-NMR (125 MHz, DMSO-d_6) spectrum of compound **22**

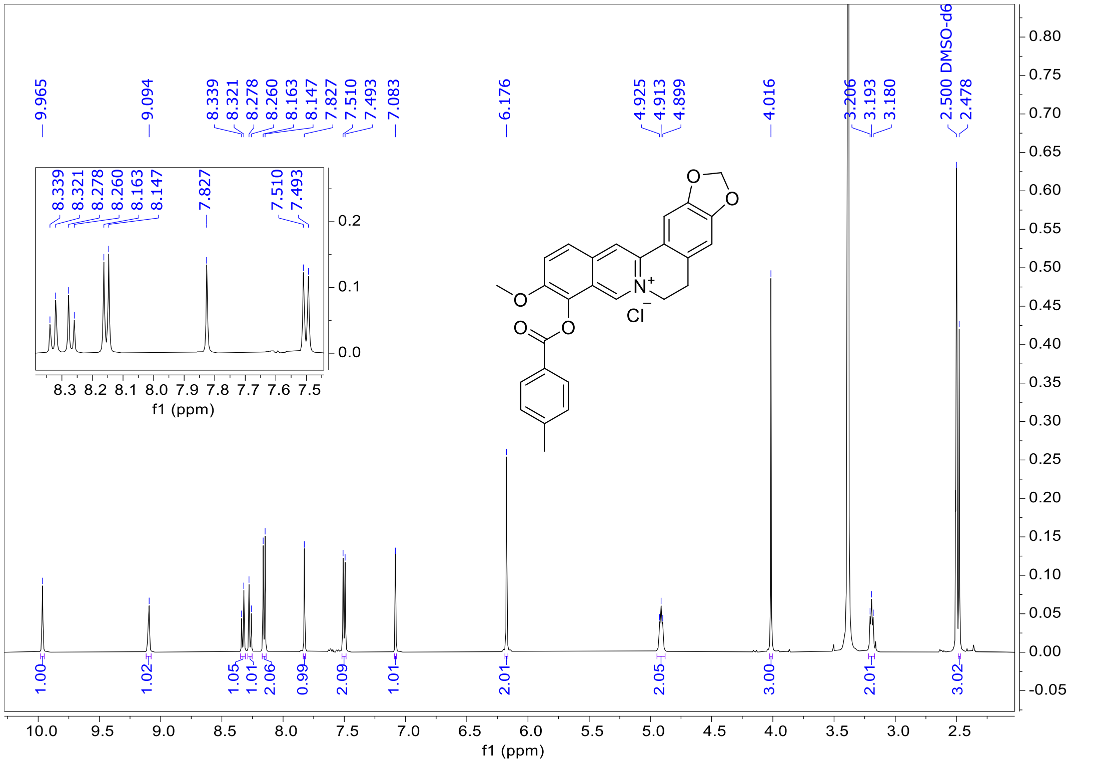

Figure S49: ^{1}H-NMR (500 MHz, DMSO-d_6) spectrum of compound **23**

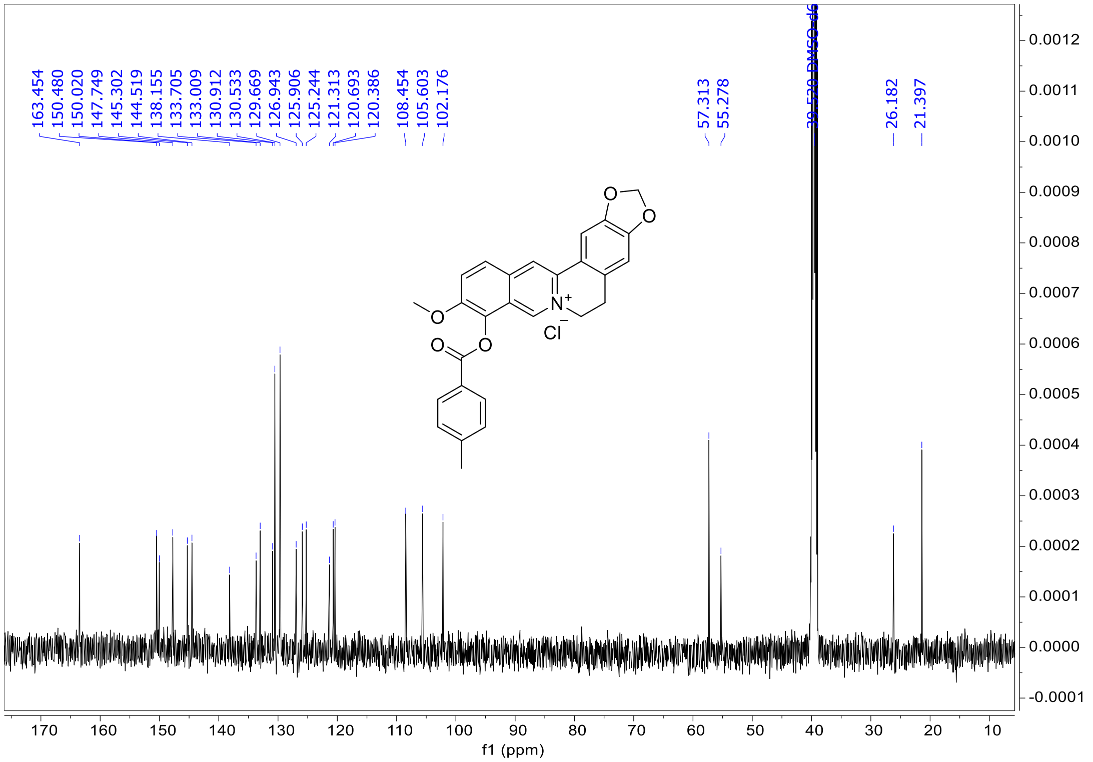

Figure S50: ^{13}C-NMR (125 MHz, DMSO-d_6) spectrum of compound **23**

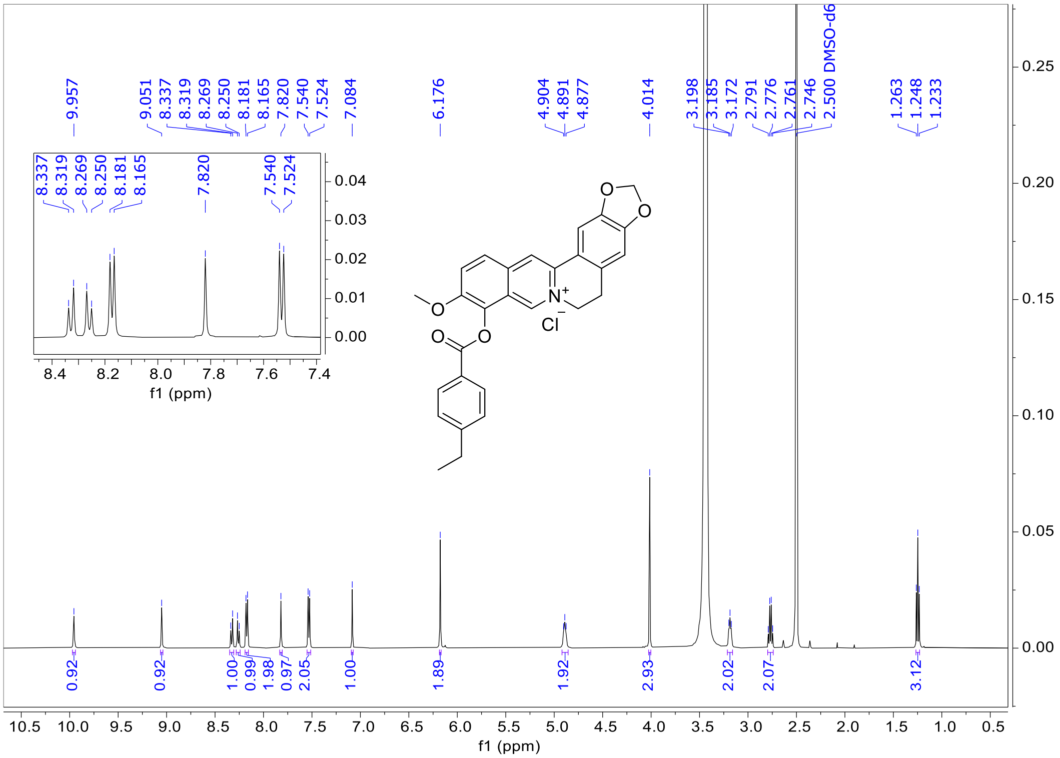

Figure S51: ^{1}H-NMR (500 MHz, DMSO-d_6) spectrum of compound **24**

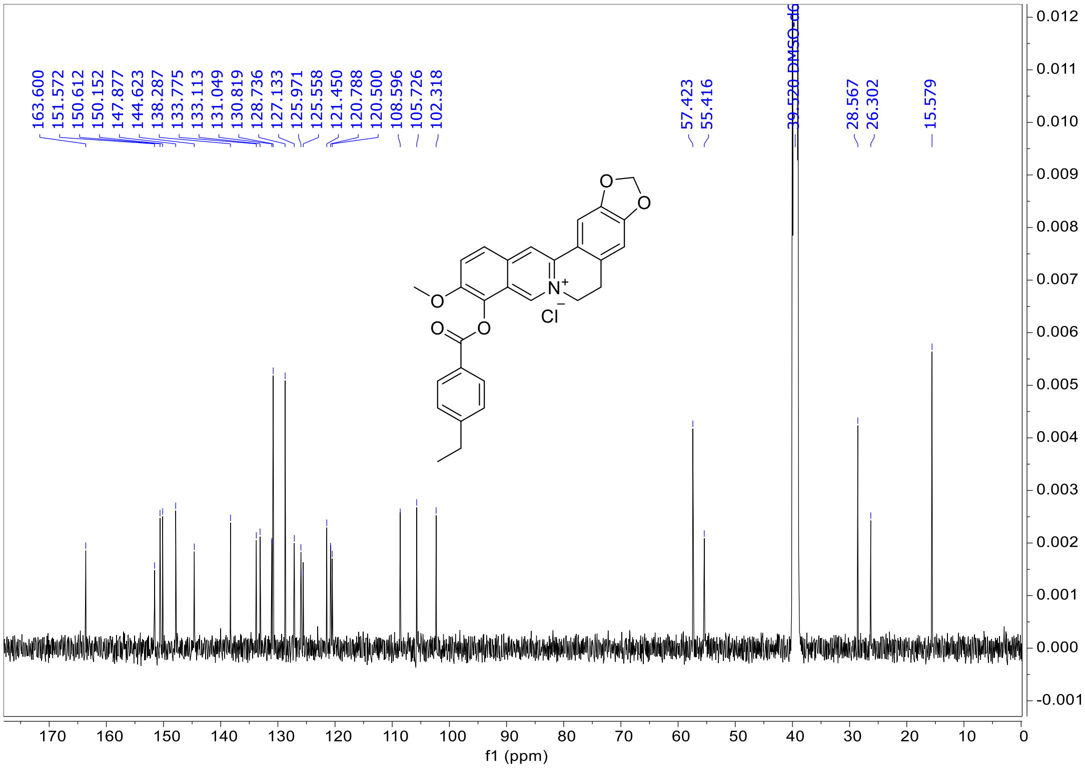

Figure S52: ^{13}C-NMR (125 MHz, DMSO-d_6) spectrum of compound **24**

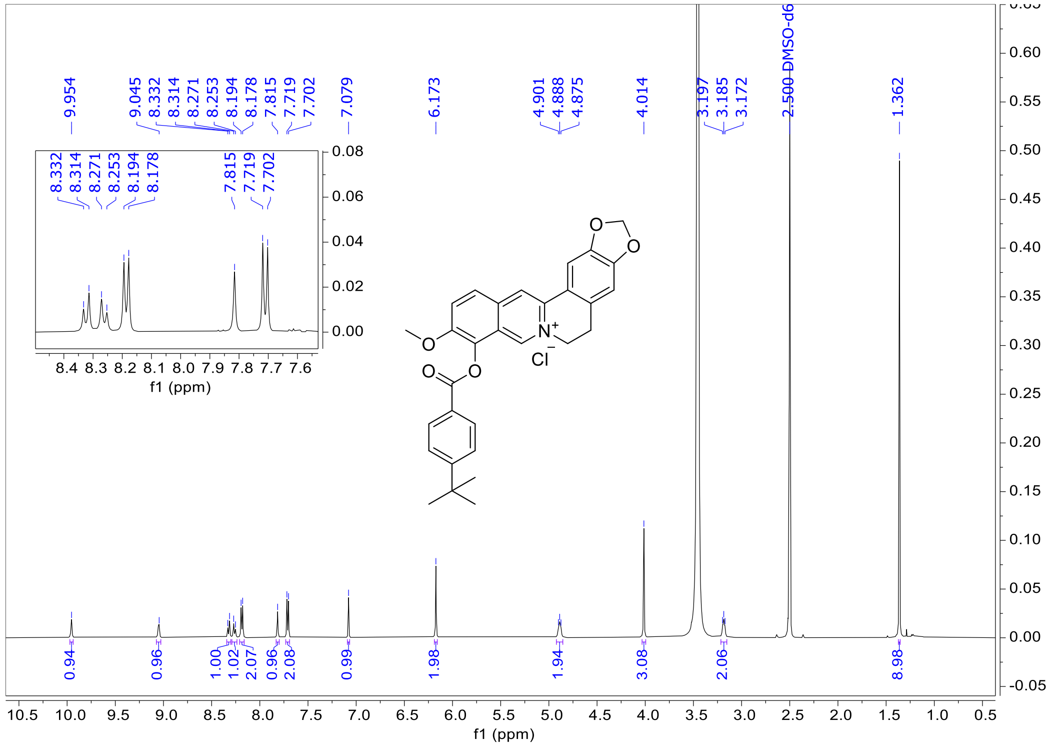

Figure S53: ^{1}H-NMR (500 MHz, DMSO-d_6) spectrum of compound **25**

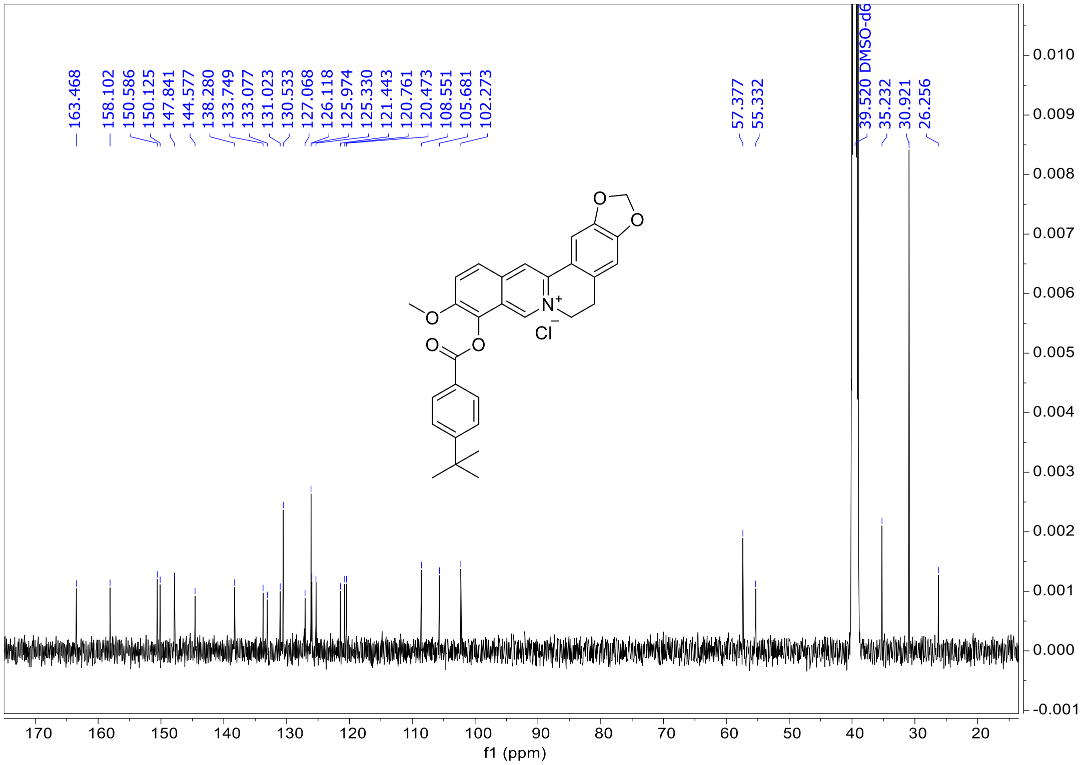

Figure S54: ^{13}C-NMR (125 MHz, DMSO-d_6) spectrum of compound **25**

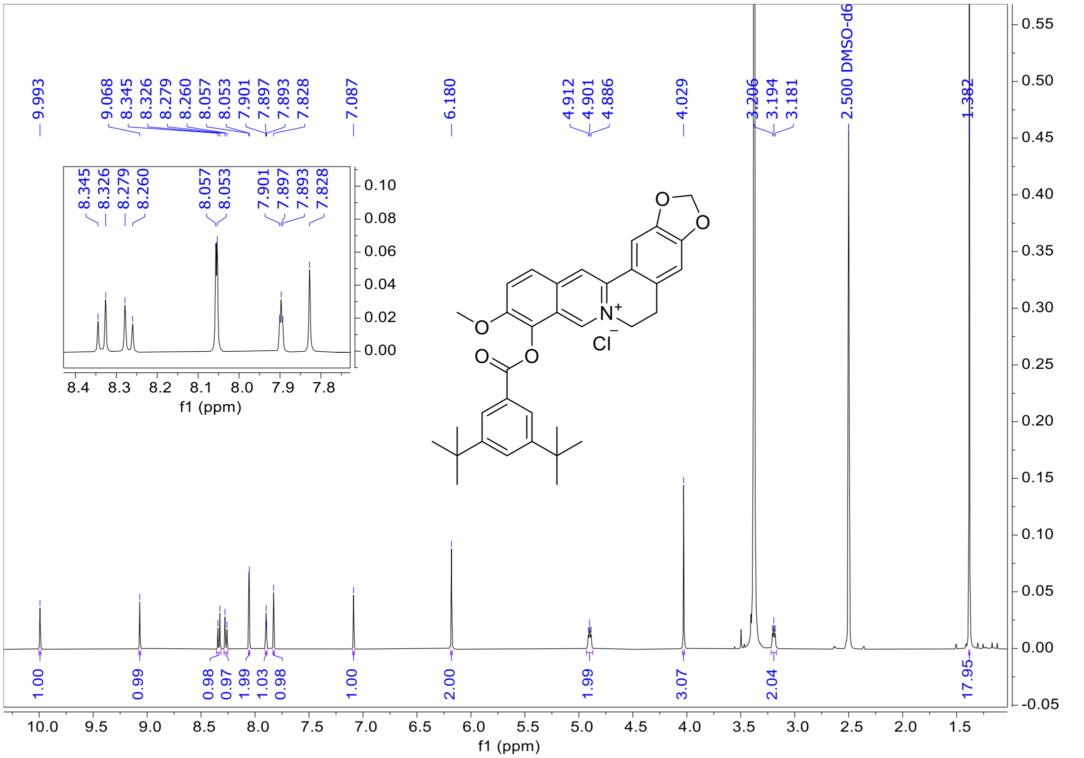

Figure S55: ^{1}H-NMR (500 MHz, DMSO-d_6) spectrum of compound **26**

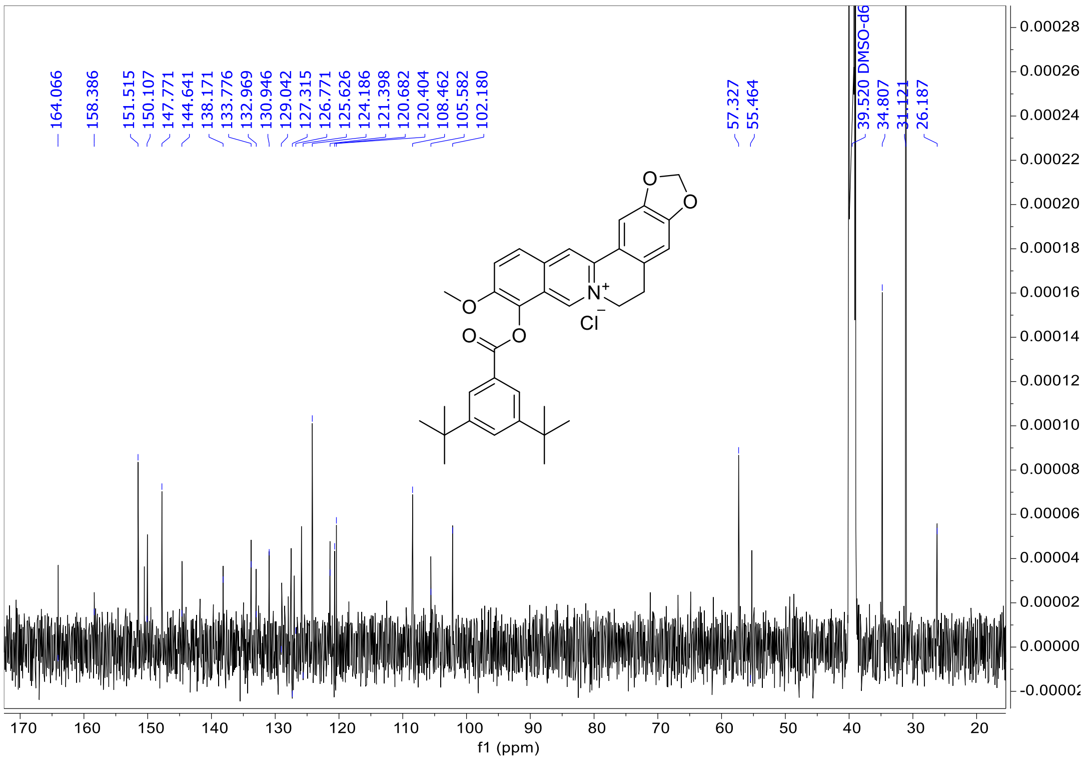

Figure S56: ^{13}C-NMR (125 MHz, DMSO-d_6) spectrum of compound **26**

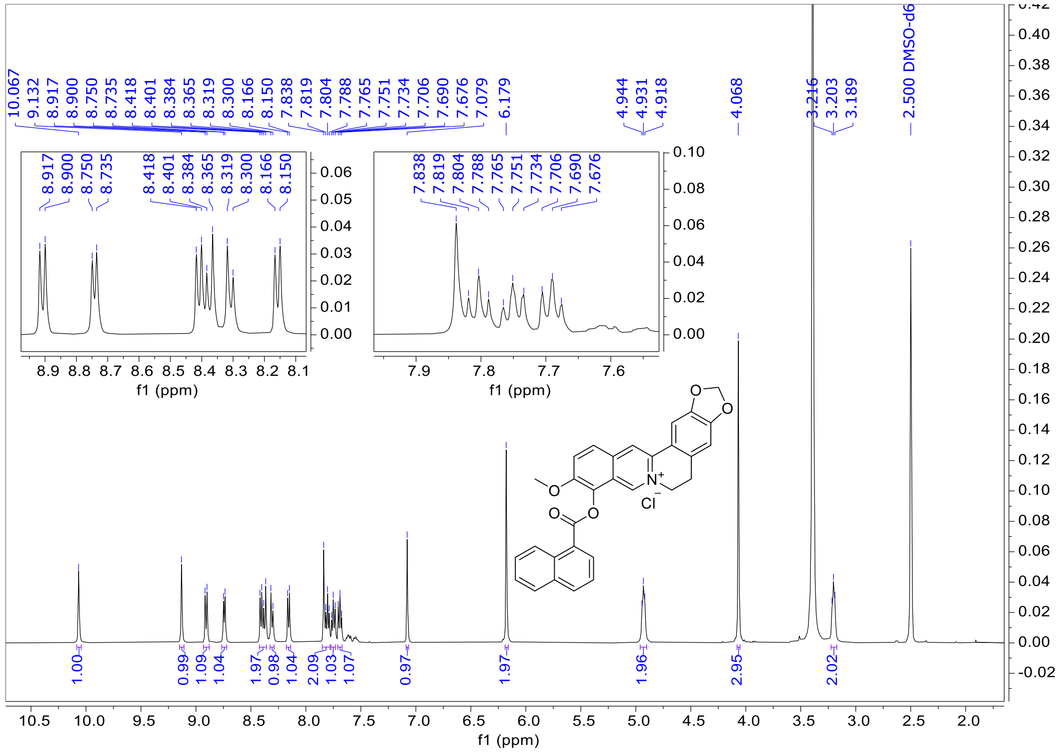

Figure S57: ^{1}H-NMR (500 MHz, DMSO-d_6) spectrum of compound **27**

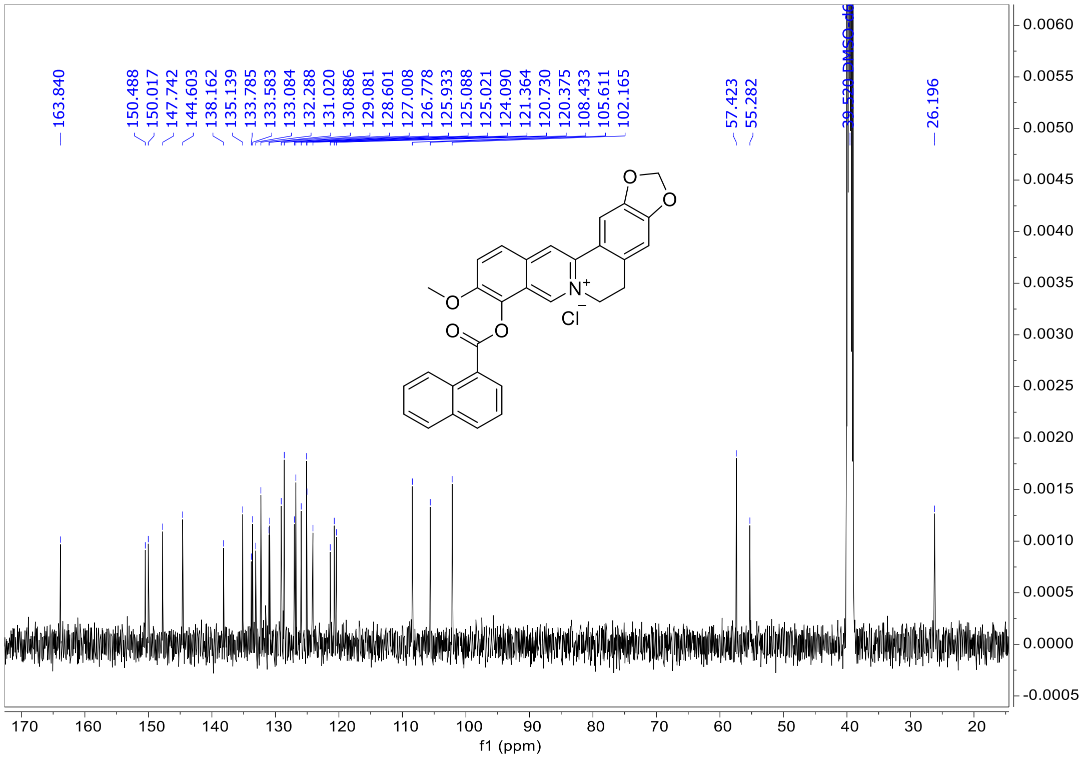

Figure S58: ^{13}C-NMR (125 MHz, DMSO-d_6) spectrum of compound **27**

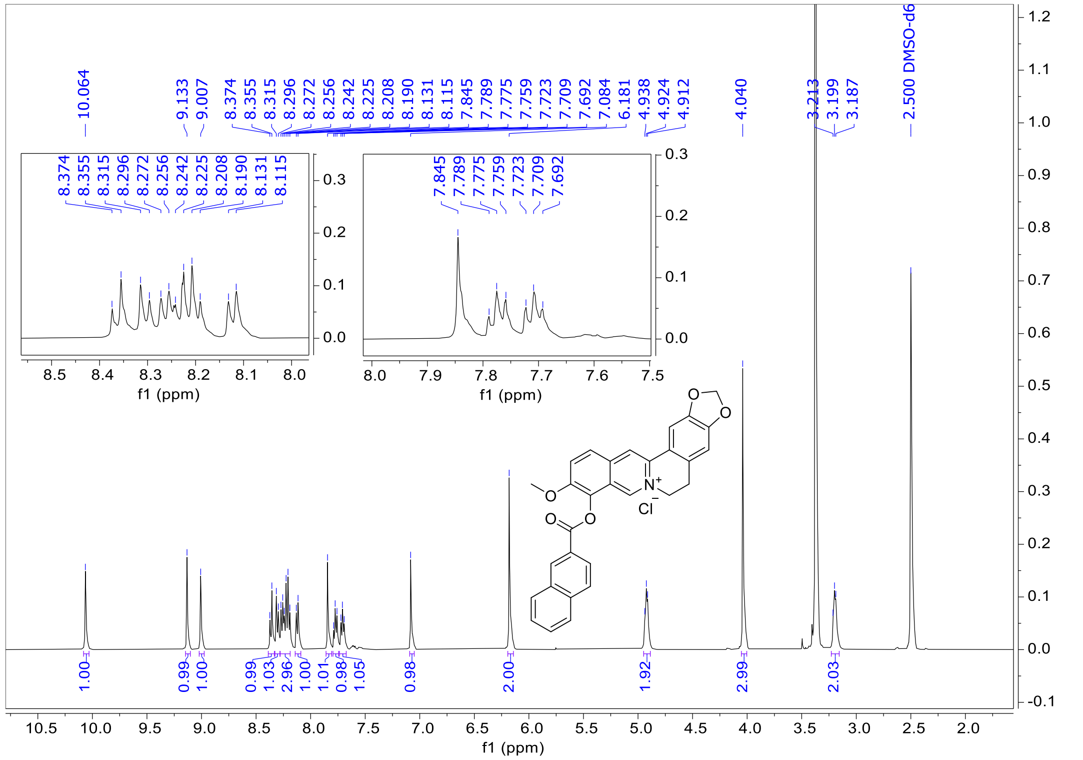

Figure S59: ^{1}H-NMR (500 MHz, DMSO-d_6) spectrum of compound **28**

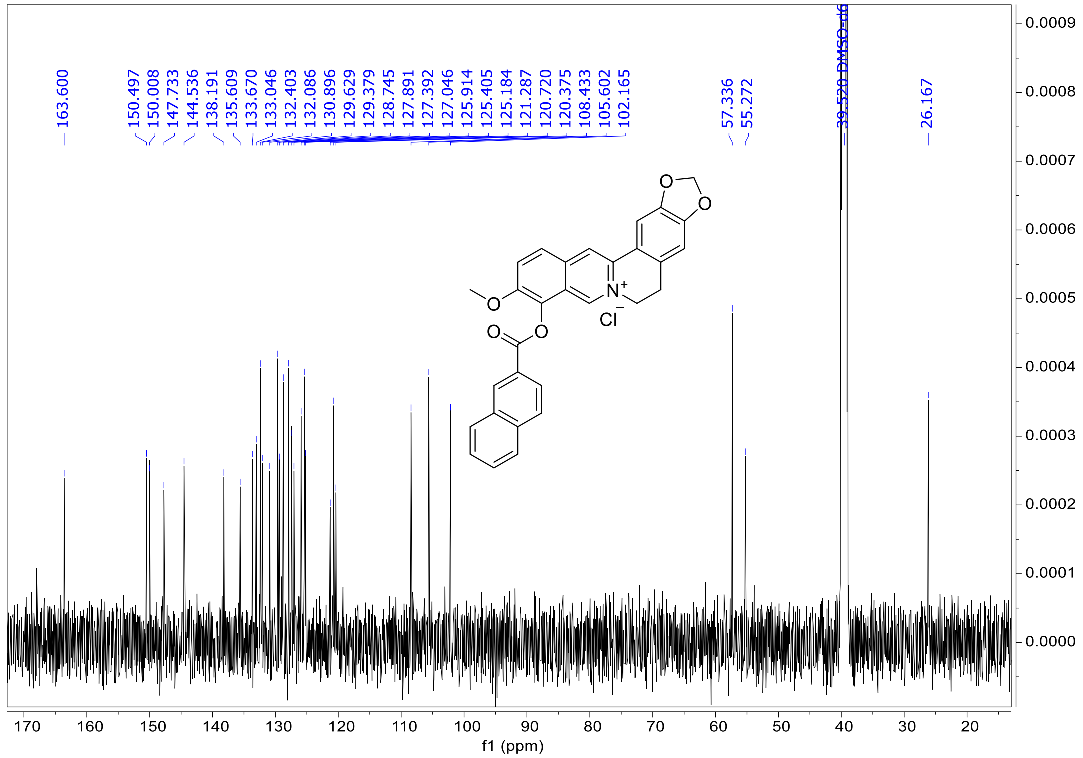

Figure S60: ^{13}C-NMR (125 MHz, DMSO-d_6) spectrum of compound **28**

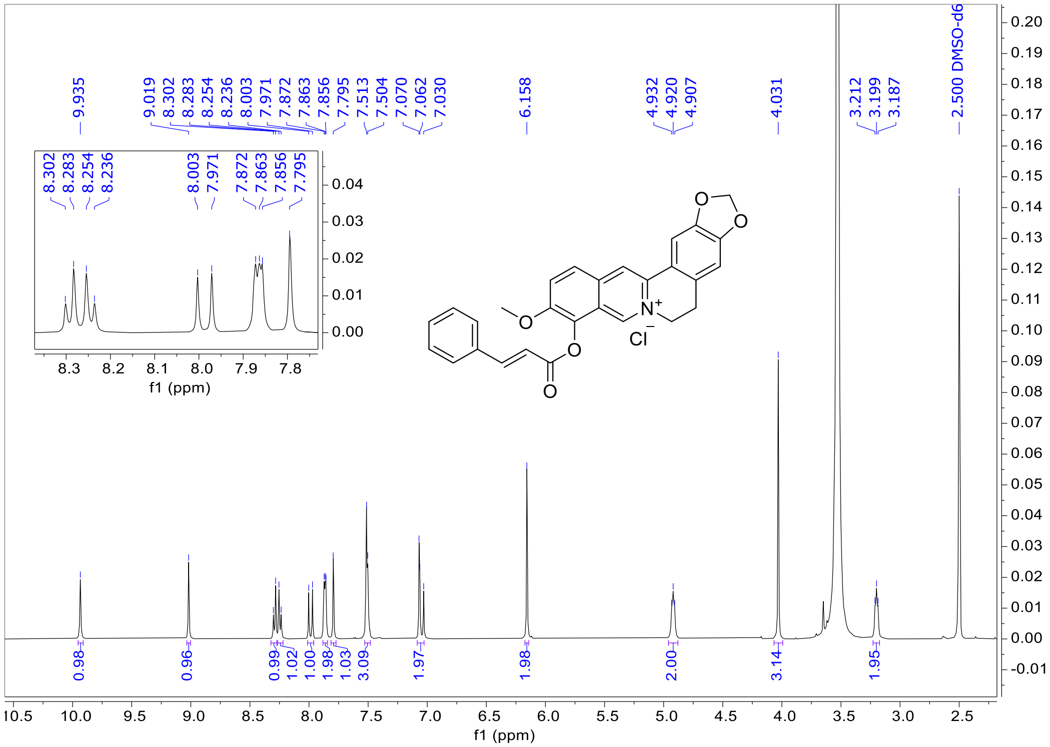

Figure S61: ^{1}H-NMR (500 MHz, DMSO-d_6) spectrum of compound **29**

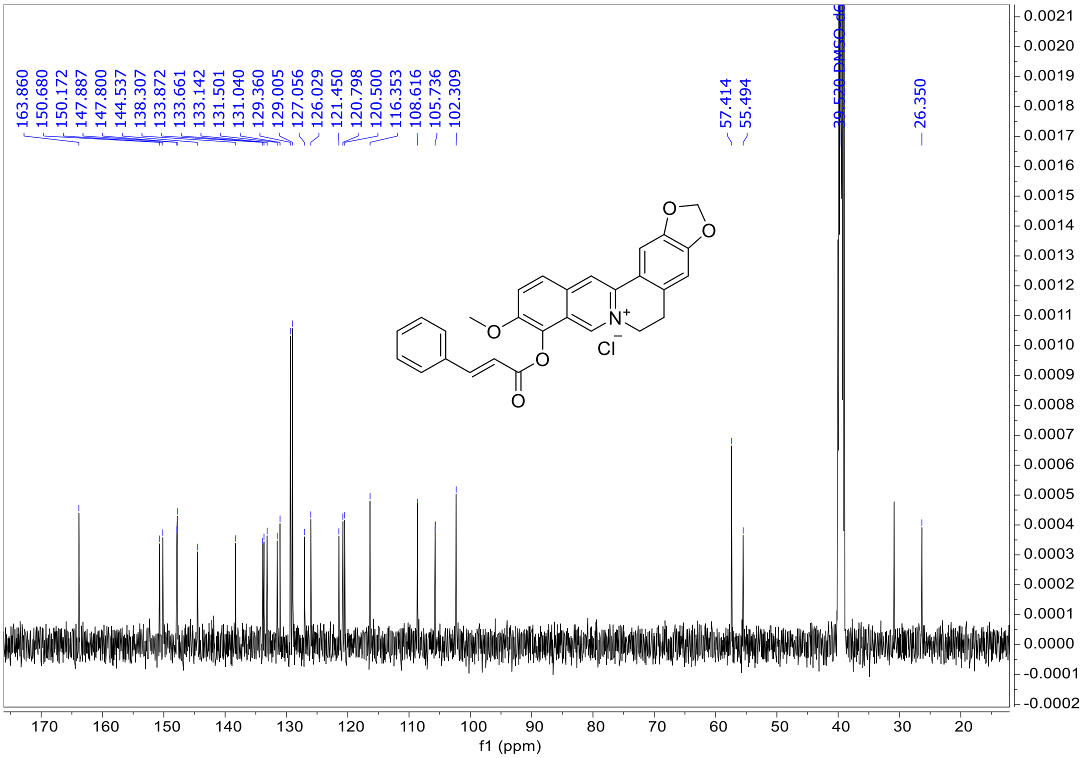

Figure S62: ^{13}C-NMR (125 MHz, DMSO-d_6) spectrum of compound **29**

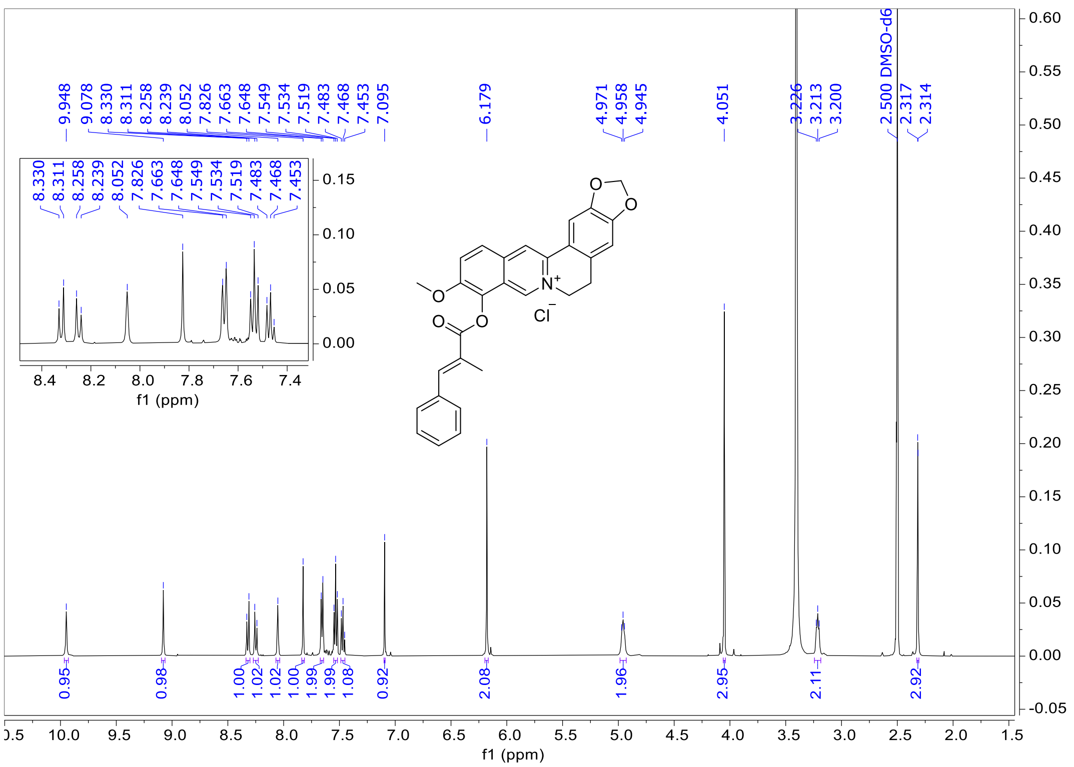

Figure S63: ^{1}H-NMR (500 MHz, DMSO-d_6) spectrum of compound **30**

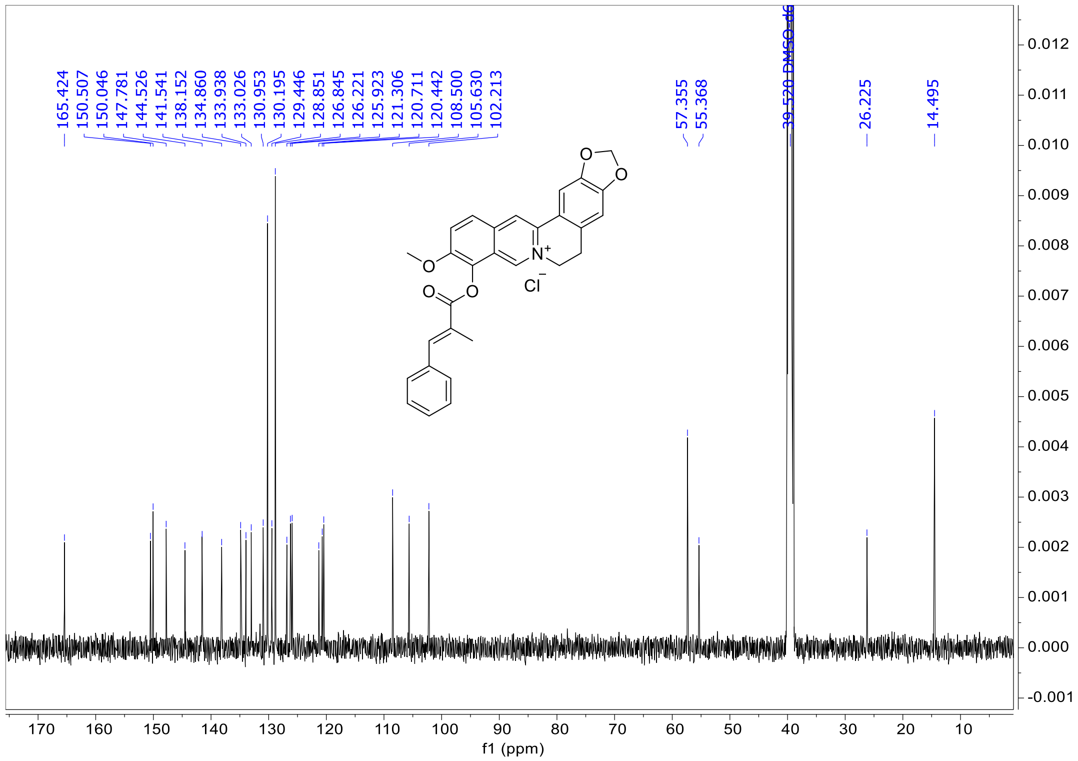

Figure S64: ^{13}C-NMR (125 MHz, DMSO-d_6) spectrum of compound **30**

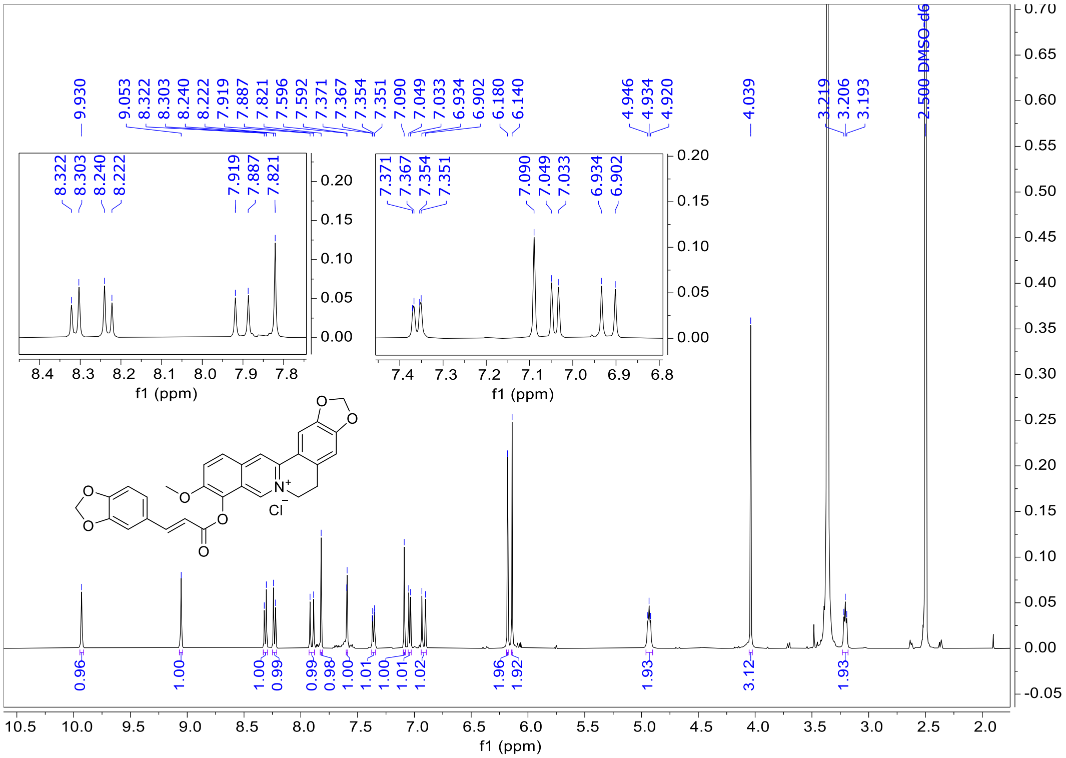

Figure S65: ^{1}H-NMR (500 MHz, DMSO-d_6) spectrum of compound **31**

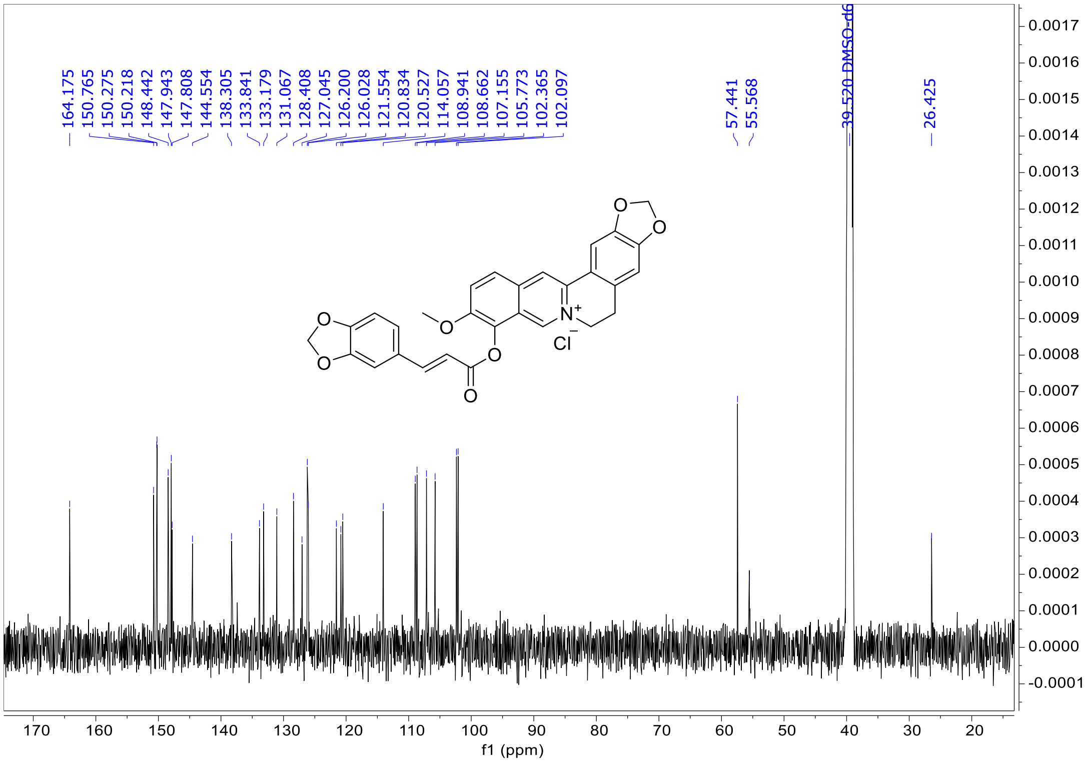

Figure S66: ^{13}C-NMR (125 MHz, DMSO-d_6) spectrum of compound **31**

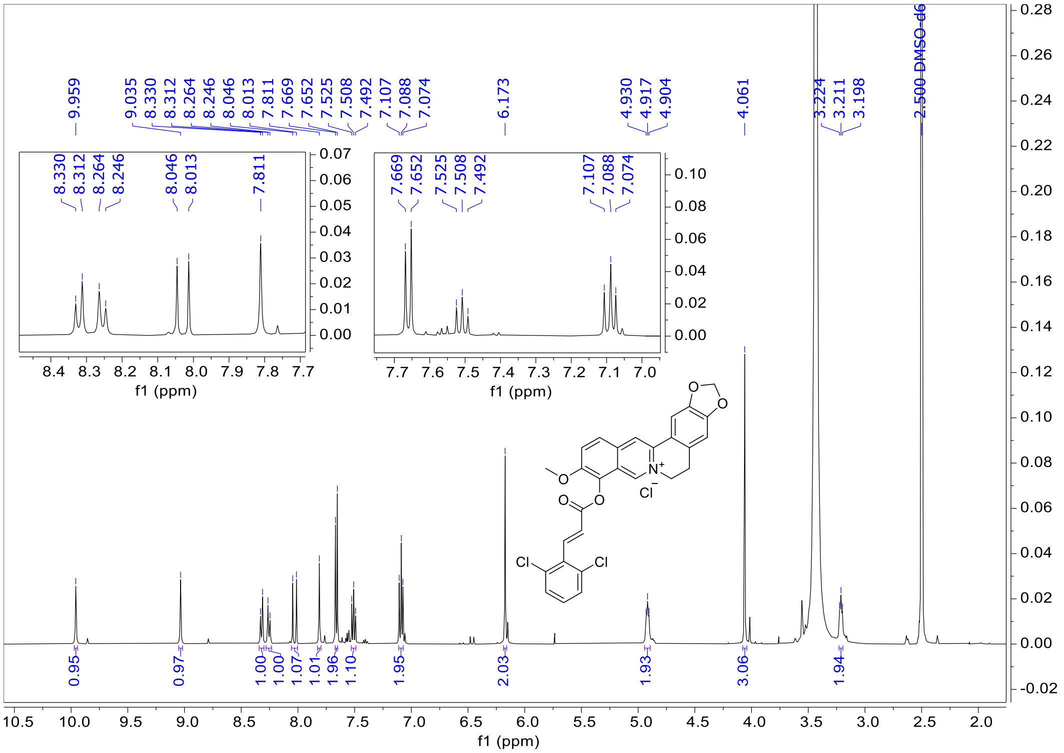

Figure S67: ^{1}H-NMR (500 MHz, DMSO-d_6) spectrum of compound **32**

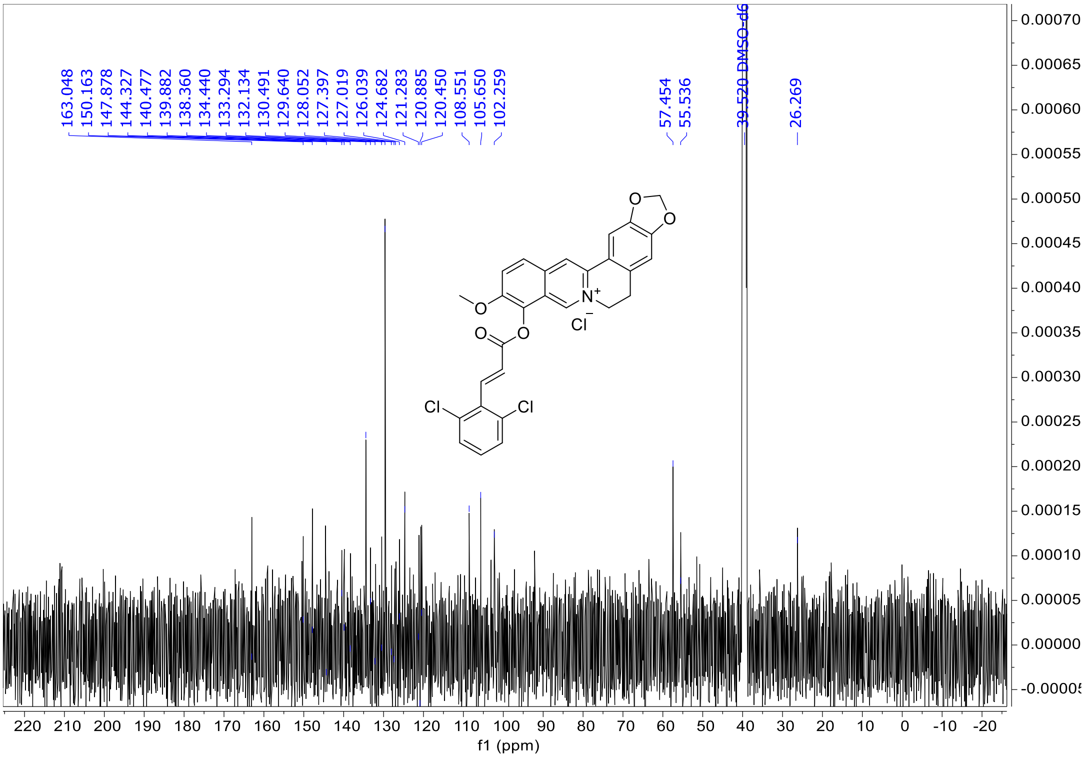

Figure S68: ^{13}C-NMR (125 MHz, DMSO-d_6) spectrum of compound **32**

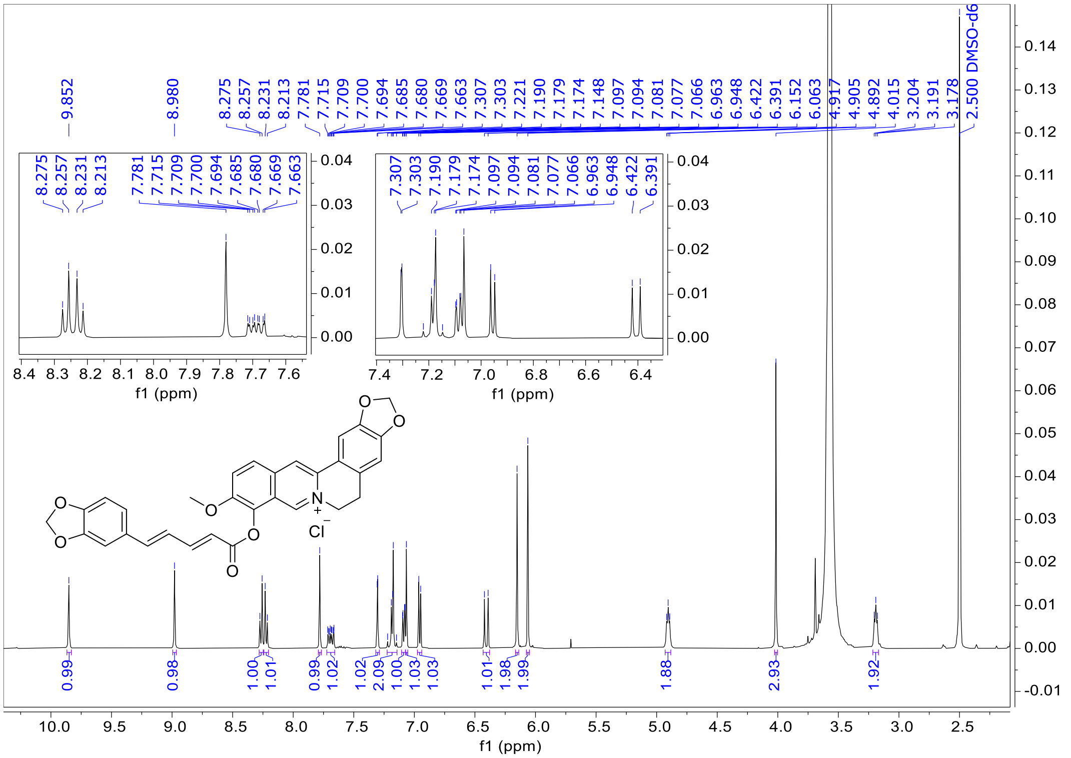

Figure S69: ^{1}H-NMR (500 MHz, DMSO-d_6) spectrum of compound **33**

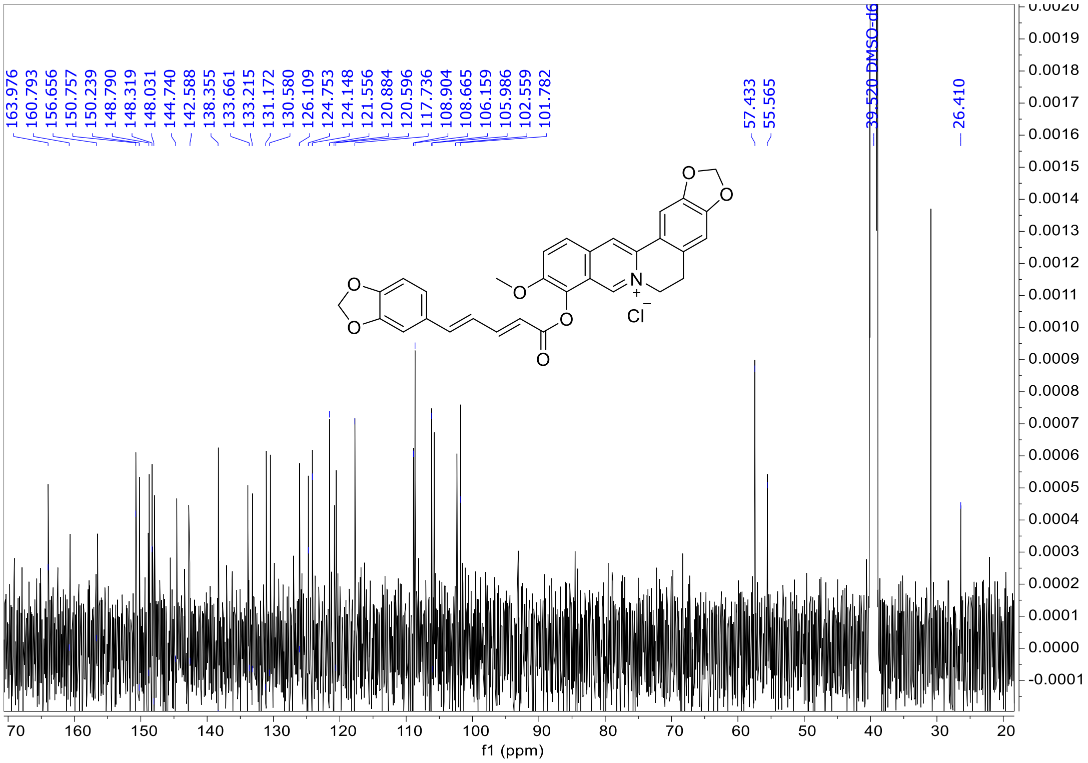

Figure S70: ^{13}C-NMR (125 MHz, DMSO-d_6) spectrum of compound **33**

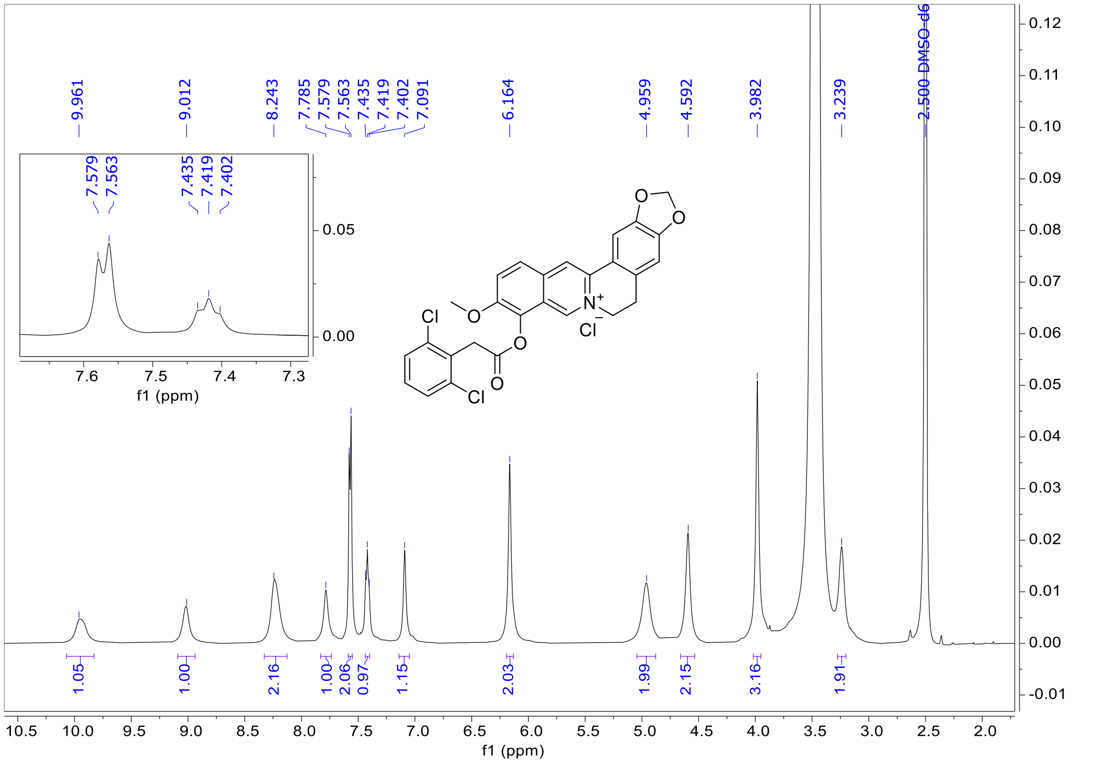

Figure S71: ^{1}H-NMR (500 MHz, DMSO-d_6) spectrum of compound **34**

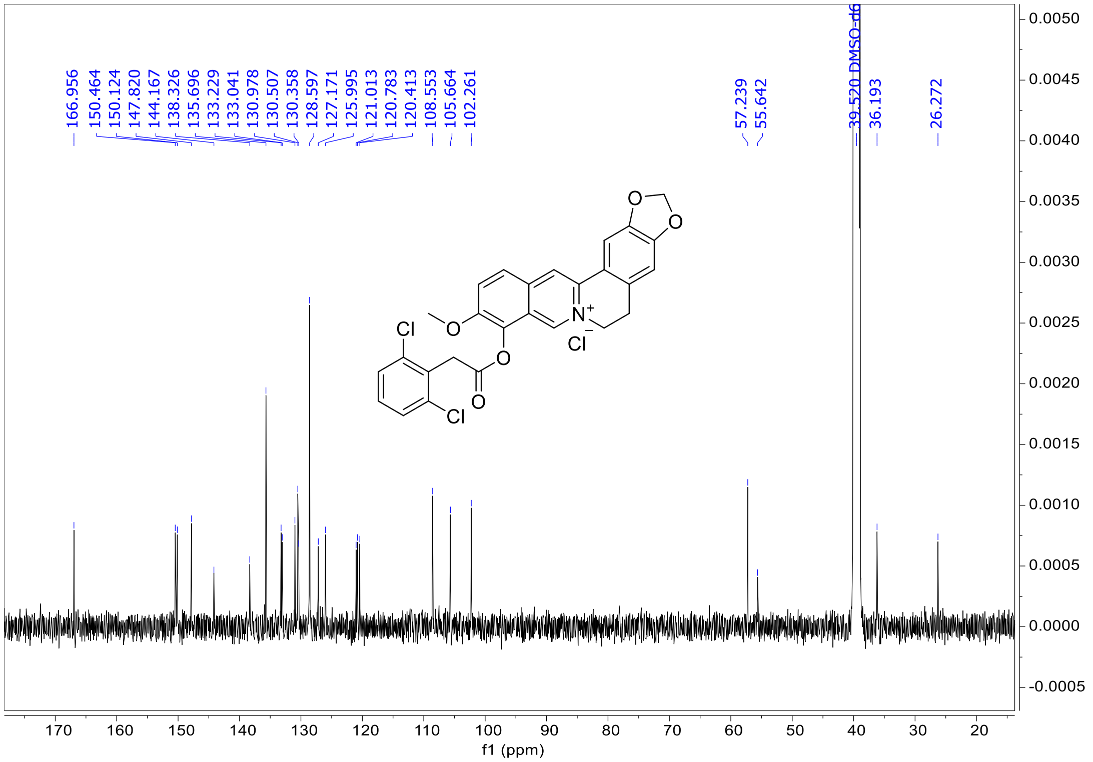

Figure S72: ^{13}C-NMR (125 MHz, DMSO-d_6) spectrum of compound **34**

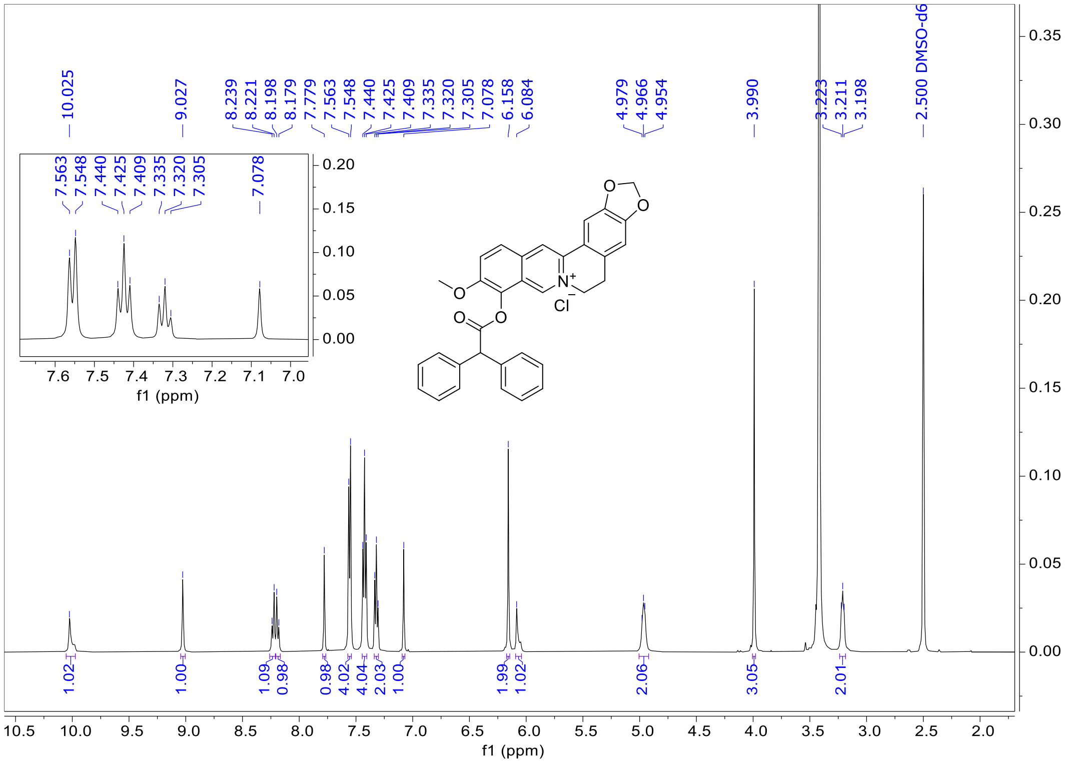

Figure S73: ^{1}H-NMR (500 MHz, DMSO-d_6) spectrum of compound **35**

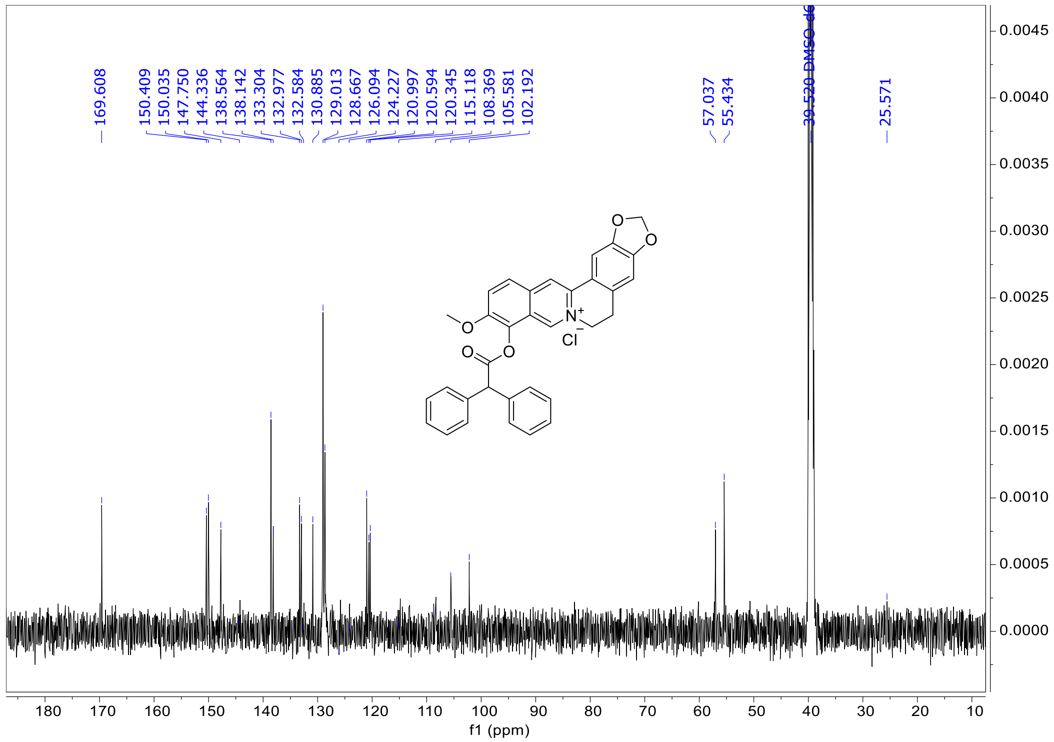

Figure S74: ^{13}C-NMR (125 MHz, DMSO-d_6) spectrum of compound **35**

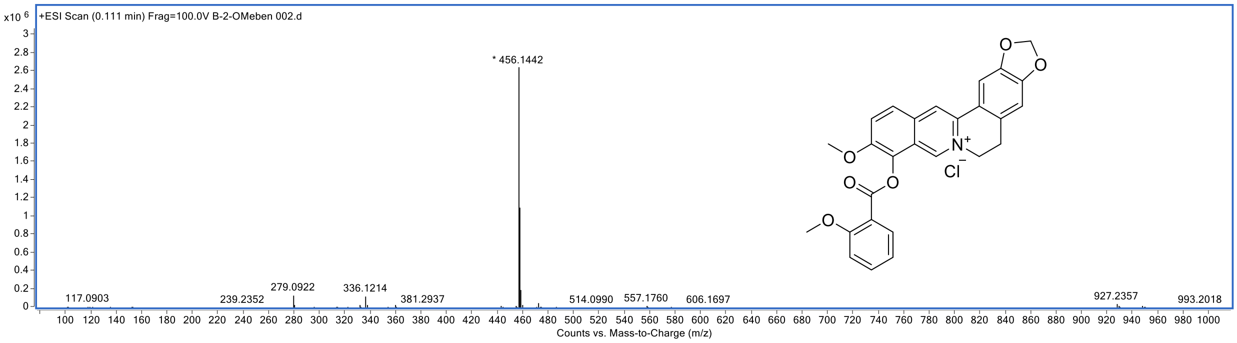

Figure S75: HRESIMS spectrum of compound **2**

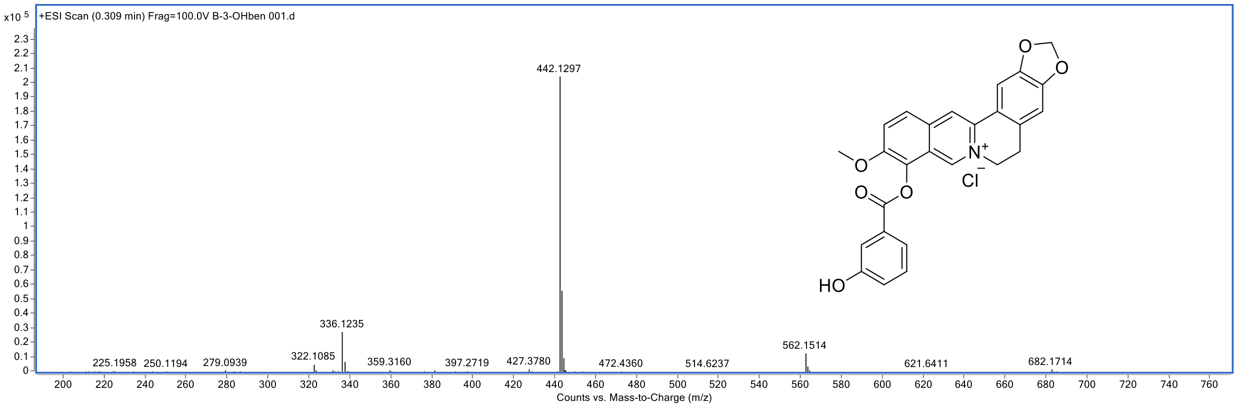

Figure S76: HRESIMS spectrum of compound **5**

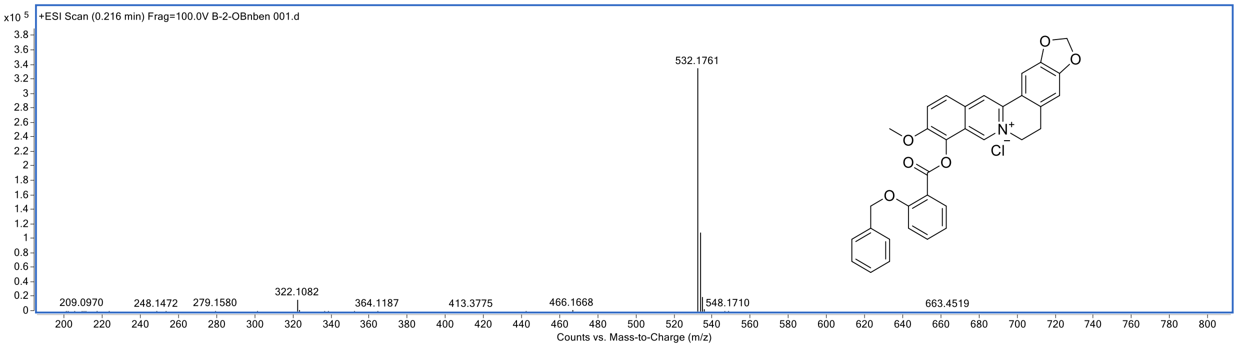

Figure S77: HRESIMS spectrum of compound **9**

Mass Spectrum List Report

Analysis Info

Analysis Name	D:\Data\Data Service\210118\B-2-Clben_RA7_01_5171.d	Acquisition Date	1/18/2021 4:13:45 PM
Method	nv_pos_5min_profile_190214.m	Operator	CU.
Sample Name	B-2-Clben	Instrument / Ser#	micrOTOF-Q II 10335
Comment			

Acquisition Parameter

Source Type	ESI	Ion Polarity	Positive	Set Nebulizer	3.0 Bar
Focus	Not active	Set Capillary	4000 V	Set Dry Heater	200 ℃
Scan Begin	100 m/z	Set End Plate Offset	-500 V	Set Dry Gas	8.0 l/min
Scan End	1500 m/z	Set Collision Cell RF	250.0 Vpp	Set Divert Valve	Waste

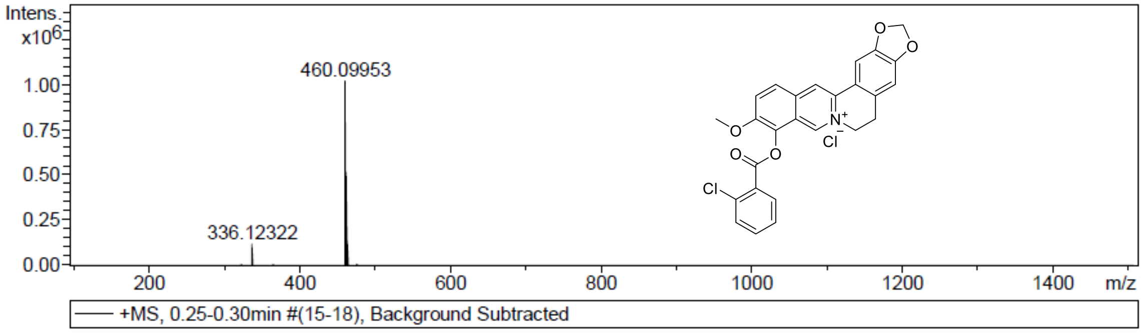

Figure S78: HRESIMS spectrum of compound **10**

Mass Spectrum List Report

Analysis Info

Analysis Name	D:\Data\Data Service\210118\B-3-Clben_RA6_01_5170.d	Acquisition Date	1/18/2021 4:07:21 PM
Method	nv_pos_5min_profile_190214.m	Operator	CU.
Sample Name	B-3-Clben	Instrument / Ser#	micrOTOF-Q II 10335
Comment			

Acquisition Parameter

Source Type	ESI	Ion Polarity	Positive	Set Nebulizer	3.0 Bar
Focus	Not active	Set Capillary	4000 V	Set Dry Heater	200 ℃
Scan Begin	100 m/z	Set End Plate Offset	-500 V	Set Dry Gas	8.0 l/min
Scan End	1500 m/z	Set Collision Cell RF	250.0 Vpp	Set Divert Valve	Waste

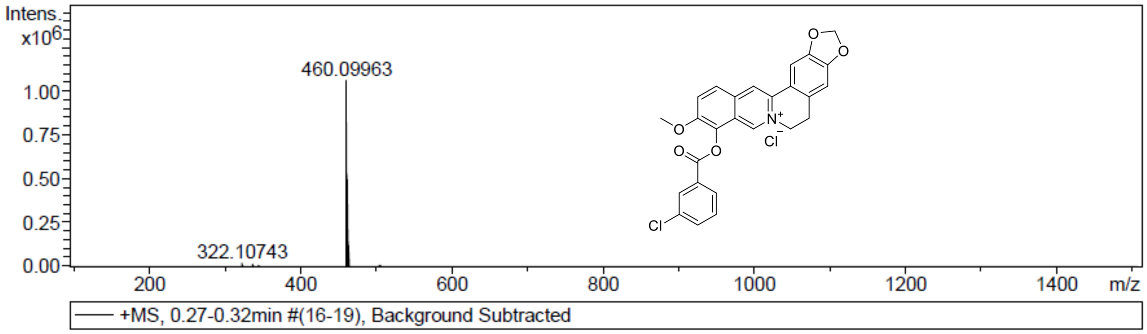

Figure S79: HRESIMS spectrum of compound **11**

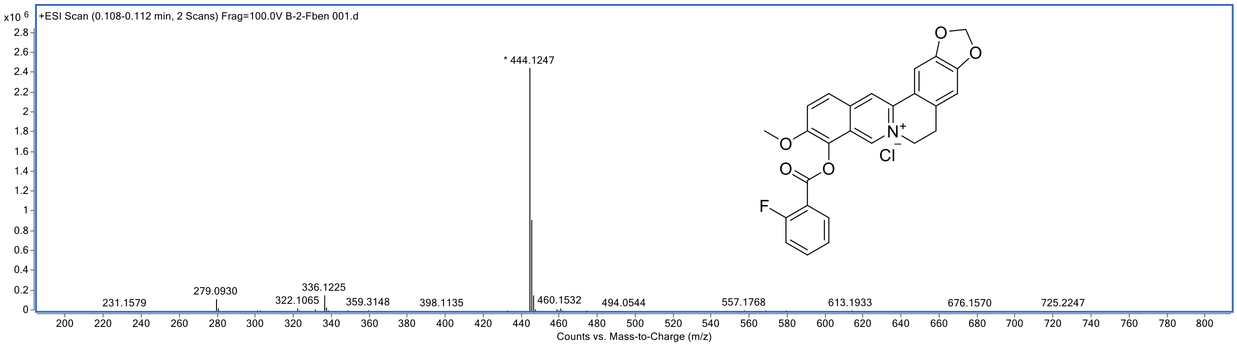

Figure S80: HRESIMS spectrum of compound **13**

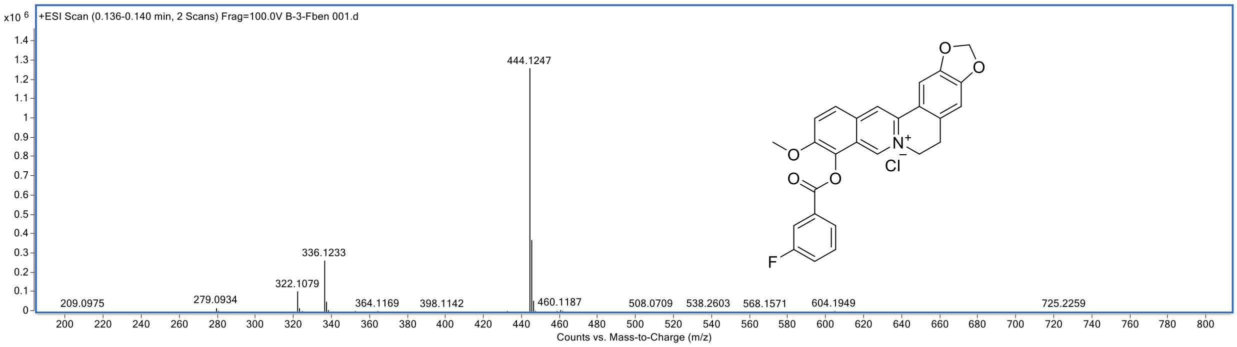

Figure S81: HRESIMS spectrum of compound **14**

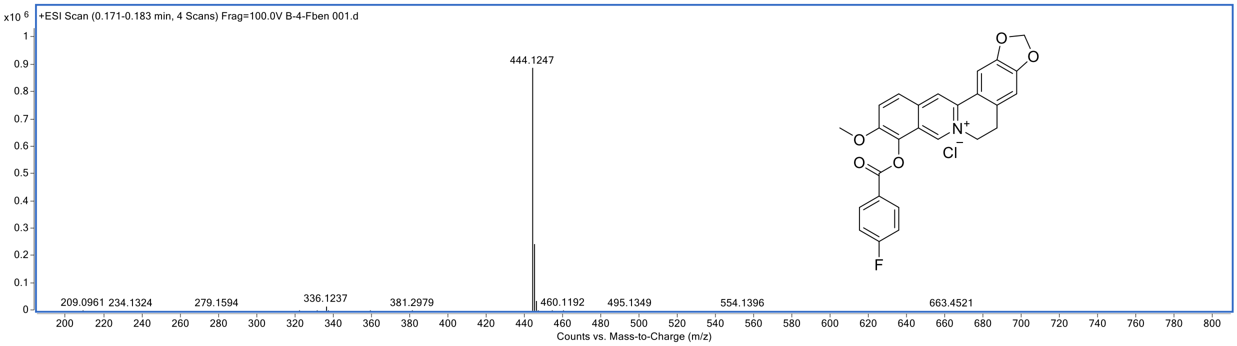

Figure S82: HRESIMS spectrum of compound **15**

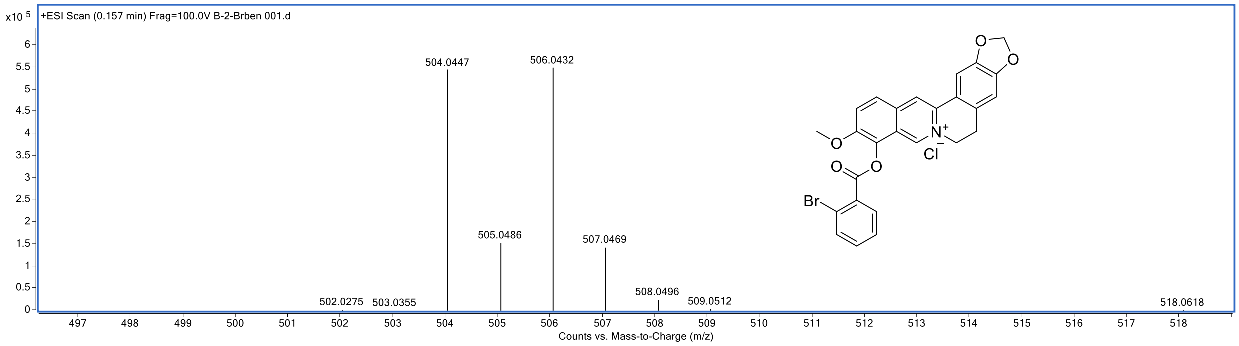

Figure S83: HRESIMS spectrum of compound **16**

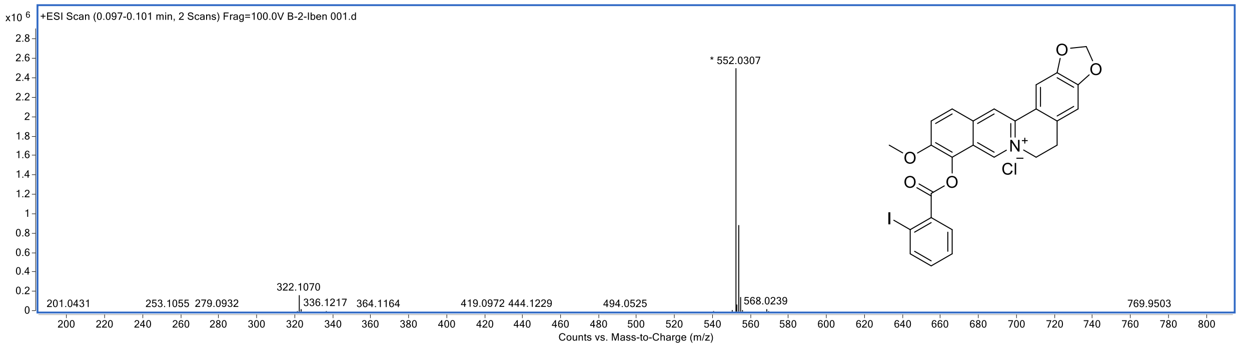

Figure S84: HRESIMS spectrum of compound **17**

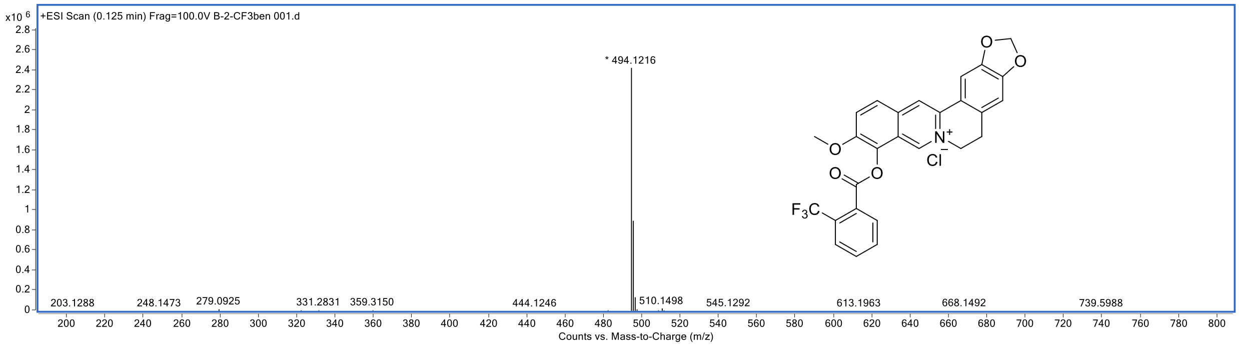

Figure S85: HRESIMS spectrum of compound **18**

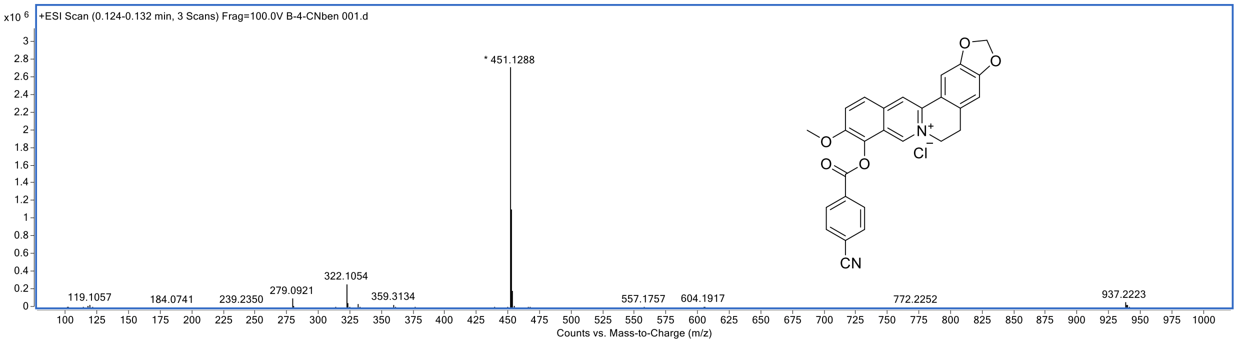

Figure S86: HRESIMS spectrum of compound **20**

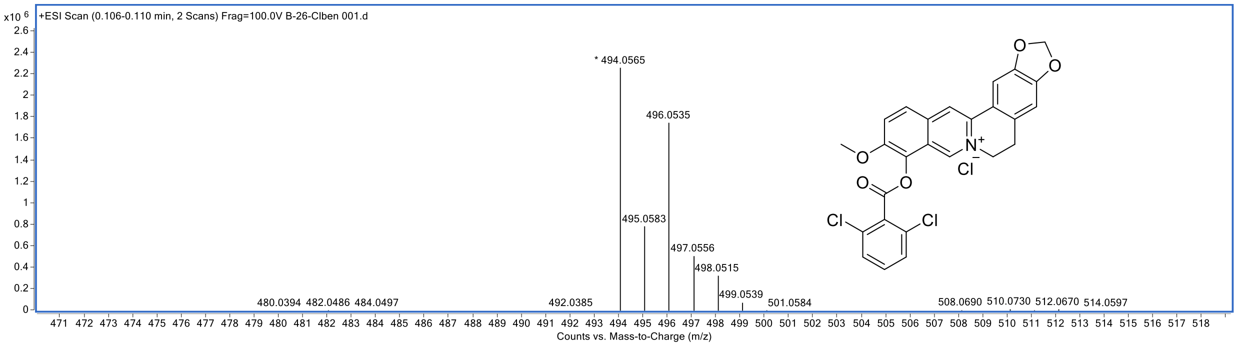

Figure S87: HRESIMS spectrum of compound **21**

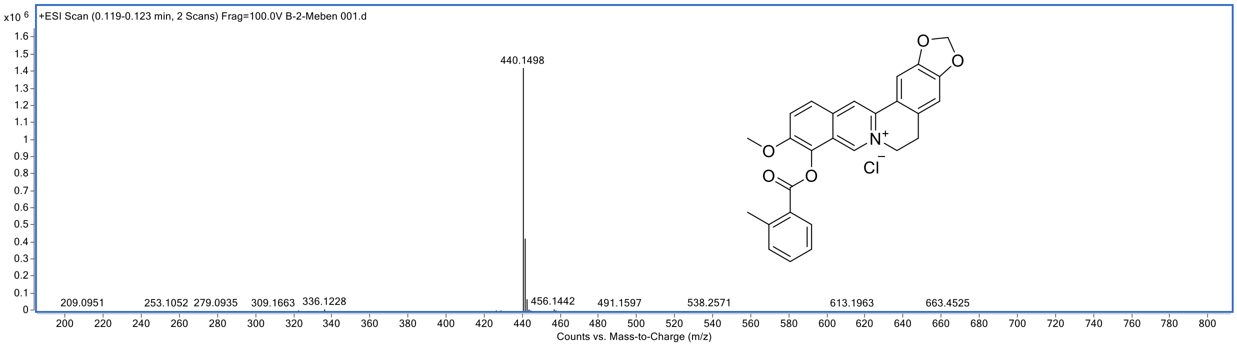

Figure S88: HRESIMS spectrum of compound **22**

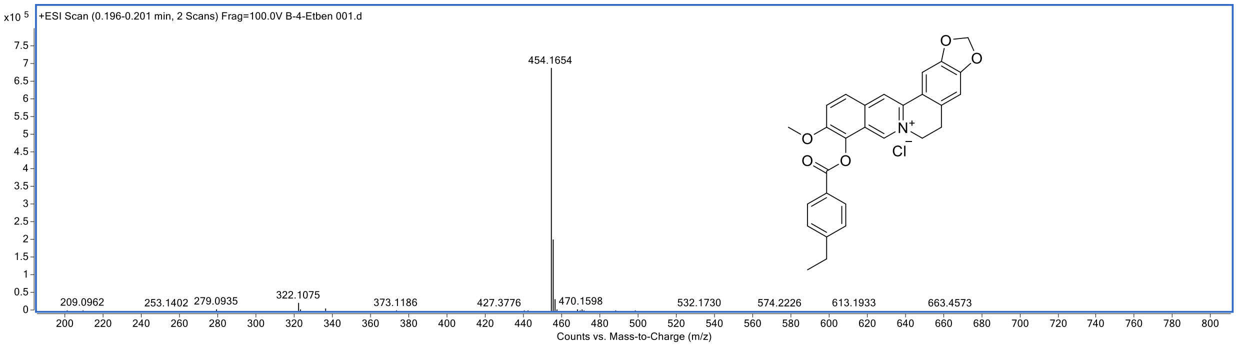

Figure S89: HRESIMS spectrum of compound **24**

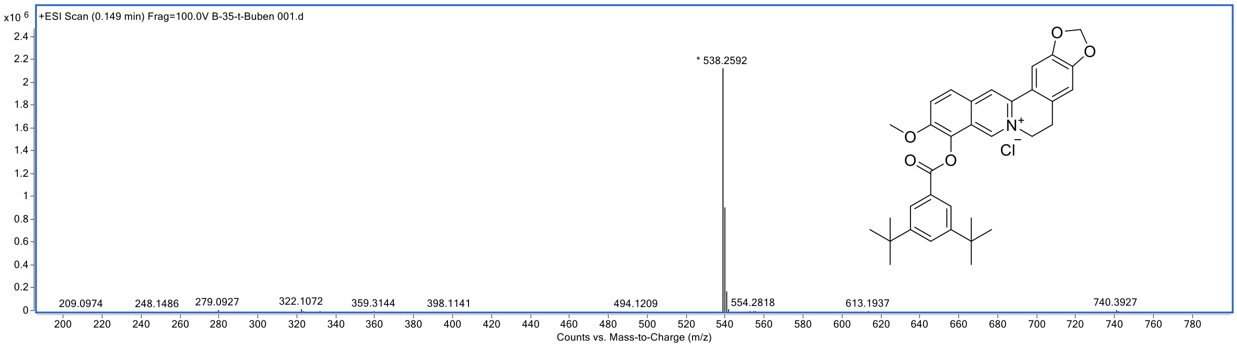

Figure S90: HRESIMS spectrum of compound **26**

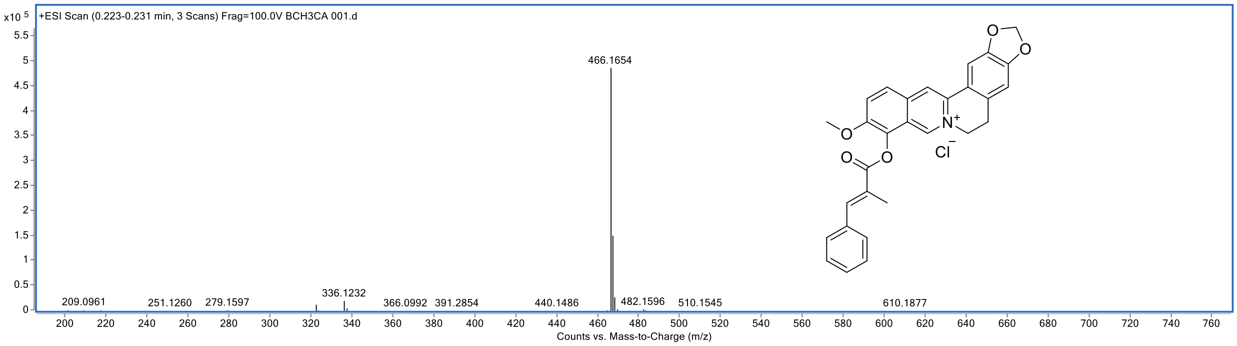

Figure S91: HRESIMS spectrum of compound **30**

Mass Spectrum List Report

Analysis Info

Analysis Name	D:\Data\Data Service\210111\B-26-ClCA_RD1_01_5152.d	Acquisition Date	1/11/2021 6:01:53 PM
Method	nv_pos_5min_profile_190214.m	Operator	CU.
Sample Name	B-26-ClCA	Instrument / Ser#	micrOTOF-Q II 10335
Comment			

Acquisition Parameter

Source Type	ESI	Ion Polarity	Positive	Set Nebulizer	3.0 Bar
Focus	Not active	Set Capillary	4000 V	Set Dry Heater	200 ℃
Scan Begin	100 m/z	Set End Plate Offset	-500 V	Set Dry Gas	8.0 l/min
Scan End	1500 m/z	Set Collision Cell RF	250.0 Vpp	Set Divert Valve	Waste

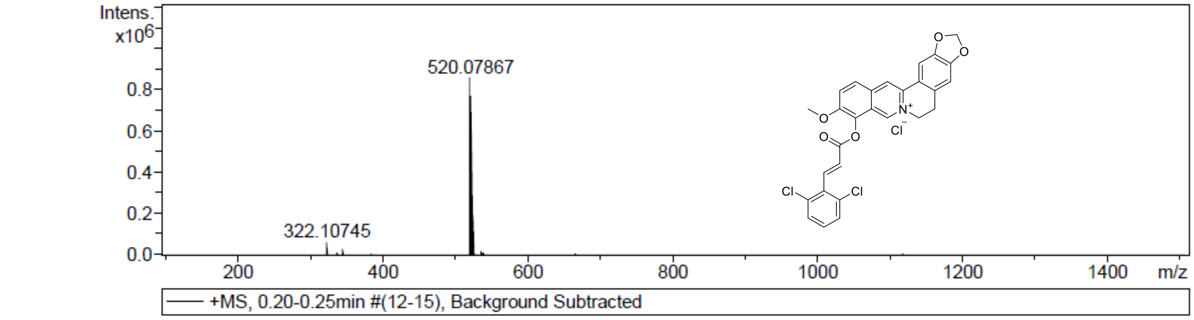

Figure S92: HRESIMS spectrum of compound **32**

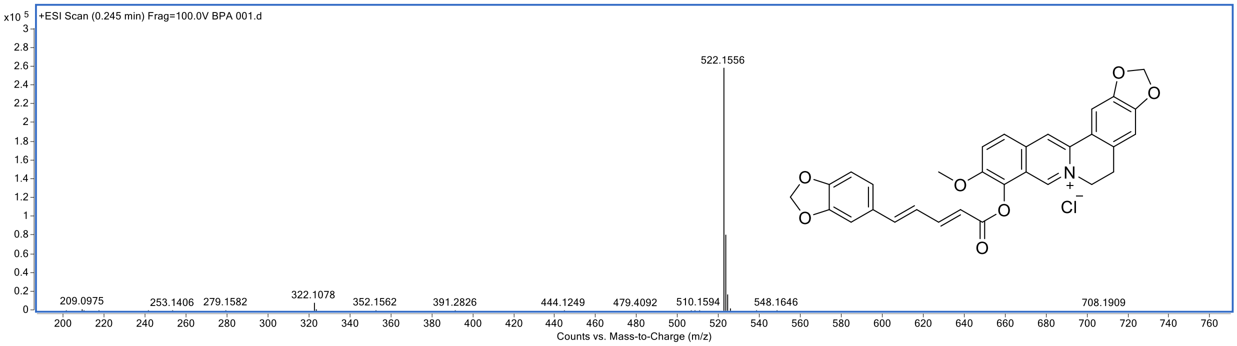

Figure S93: HRESIMS spectrum of compound **33**

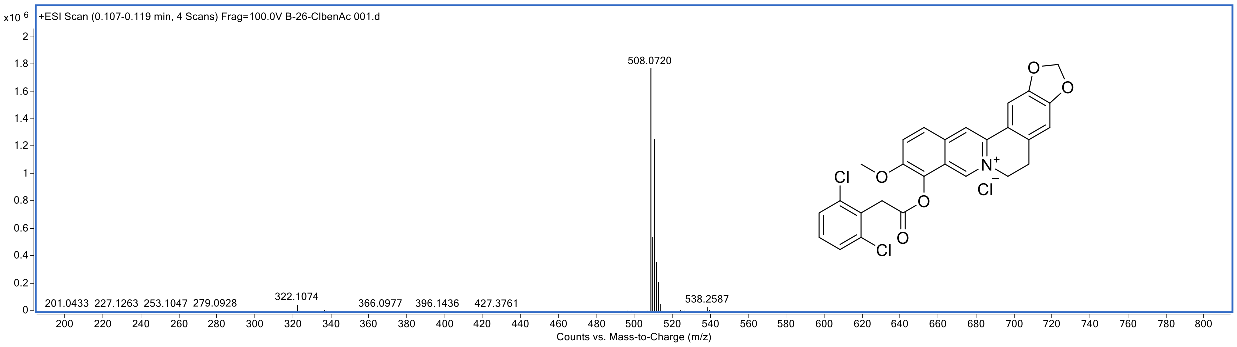

Figure S94: HRESIMS spectrum of compound **34**

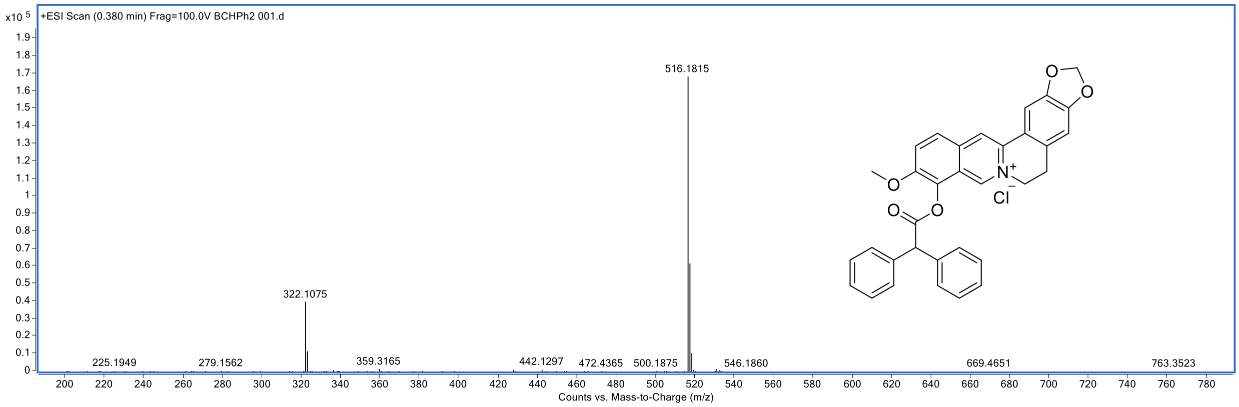

Figure S95: HRESIMS spectrum of compound **35**

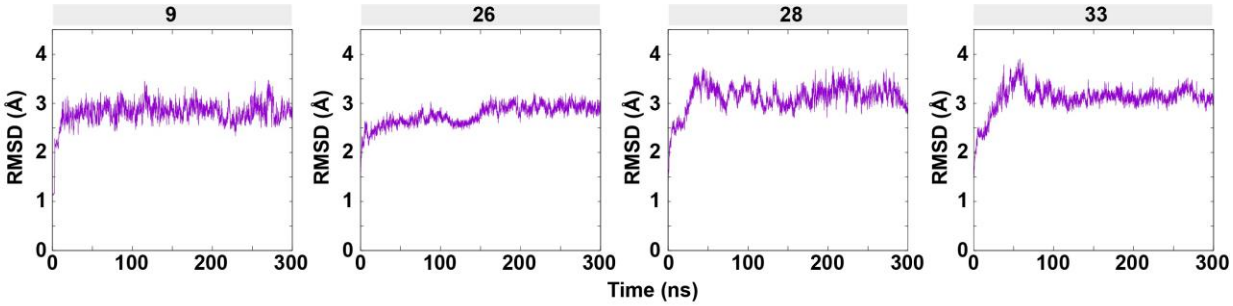

Figure S96: RMSD plot of ligand/protein complex during 300-MD simulations.

Sequence Alignment

```
########################################
# Program: needle
# Rundate: Thu  9 Mar 2023 06:52:32
# Commandline: needle
#    -auto
#    -stdout
#    -asequence emboss_needle-I20230309-065149-0668-26923266-p2m.asequence
#    -bsequence emboss_needle-I20230309-065149-0668-26923266-p2m.bsequence
#    -datafile EBLOSUM62
#    -gapopen 10.0
#    -gapextend 0.5
#    -endopen 10.0
#    -endextend 0.5
#    -aformat3 pair
#    -sprotein1
#    -sprotein2
# Align_format: pair
# Report_file: stdout
########################################

#=======================================
#
# Aligned_sequences: 2
# 1: PDB: 3A4A
# 2: UNIPROT: P53341
# Matrix: EBLOSUM62
# Gap_penalty: 10.0
# Extend_penalty: 0.5
#
# Length: 589
# Identity:     423/589 (71.8%)
# Similarity:   500/589 (84.9%)
# Gaps:           5/589 ( 0.8%)
# Score: 2408.0
#
#
#=======================================

PDB: 3A4A          1 MTISSAHPETEPKWWKEATFYQIYPASFKDSNDDGWGDMKGIASKLEYIK     50
                     ||||. |||||||||||||.||||||||||||:|||||:|||.|||:|||
Uniprot: P53341    1 MTISD-HPETEPKWWKEATIYQIYPASFKDSNNDGWGDLKGITSKLQYIK     49

PDB: 3A4A         51 ELGADAIWISPFYDSPQDDMGYDIANYEKVWPTYGTNEDCFALIEKTHKL    100
                     :||.||||:.|||||||.||||||:||||||||||||||||.||:|||||
UNIPROT: P53341   50 DLGVDAIWVCPFYDSPQQDMGYDISNYEKVWPTYGTNEDCFELIDKTHKL     99

PDB: 3A4A        101 GMKFITDLVINHCSSEHEWFKESRSSKTNPKRDWFFWRPPKGYDAEGKPI    150
                     |||||||||||||:||||||||||||||||||||||||||||||||||||
UNIPROT: P53341  100 GMKFITDLVINHCSTEHEWFKESRSSKTNPKRDWFFWRPPKGYDAEGKPI    149

PDB: 3A4A        151 PPNNWKSYFGGSAWTFDEKTQEFYLRLFCSTQPDLNWENEDCRKAIYESA    200
                     |||||||:||||||||||.|.|||||||.|.|.||||||||||:||:|||
UNIPROT: P53341  150 PPNNWKSFFGGSAWTFDETTNEFYLRLFASRQVDLNWENEDCRRAIFESA    199

PDB: 3A4A        201 VGYWLDHGVDGFRIDVGSLYSKVVGLPDAPVVDKNSTWQSSDPYTLNGPR    250
                     ||:||||||||||||...||||..||||:|:.||.|..|..:..:.||||
UNIPROT: P53341  200 VGFWLDHGVDGFRIDTAGLYSKRPGLPDSPIFDKTSKLQHPNWGSHNGPR    249

PDB: 3A4A        251 IHEFHQEMNQFIRNRVKDGREIMTVGEMQHASDETKRLYTSASRHELSEL    300
                     |||:|||:::|::||||||||||||||:.|.||..  |||||:|:|:||:
UNIPROT: P53341  250 IHEYHQELHRFMKNRVKDGREIMTVGEVAHGSDNA--LYTSAARYEVSEV    297
```

```
PDB: 3A4A        301 FNFSHTDVGTSPLFRYNLVPFELKDWKIALAELFRYINGTDCWSTIYLEN    350
                     |:|:|.:|||||.||||:|||.||.||.|:|..|.:|||||.|:|.|:||
UNIPROT: P53341  298 FSFTHVEVGTSPFFRYNIVPFTLKQWKEAIASNFLFINGTDSWATTYIEN    347

PDB: 3A4A        351 HDQPRSITRFGDDSPKNRVISGKLLSVLLSALTGTLYVYQGQELGQINFK    400
                     |||.||||||.|||||.|.||||||::|..:||||||||||||:||||||
UNIPROT: P53341  348 HDQARSITRFADDSPKYRKISGKLLTLLECSLTGTLYVYQGQEIGQINFK    397

PDB: 3A4A        401 NWPVEKYEDVEIRNNYNAIKEEHGENSEEMKKFLEAIALISRDHARTPMQ    450
                     .||:||||||:::|||..||:..|:||:|||.|.:.|||:||||:||||.
UNIPROT: P53341  398 EWPIEKYEDVDVKNNYEIIKKSFGKNSKEMKDFFKGIALLSRDHSRTPMP    447

PDB: 3A4A        451 WSREEPNAGFSGPSAKPWFYLNDSFREGINVEDEIKDPNSVLNFWKEALK    500
                     |::::|||||:||..||||.||:||.:|||||.|.:|.:|||||||.||:
UNIPROT: P53341  448 WTKDKPNAGFTGPDVKPWFLLNESFEQGINVEQESRDDDSVLNFWKRALQ    497

PDB: 3A4A        501 FRKAHKDITVYGYDFEFIDLDNKKLFSFTKKYNNKTLFAALNFSSDATDF    550
                     .||.:|::.:|||||:|||||:.::|||||:|.:||||||||||.:..:|
UNIPROT: P53341  498 ARKKYKELMIYGYDFQFIDLDSDQIFSFTKEYEDKTLFAALNFSGEEIEF    547

PDB: 3A4A        551 KIPNDDSSFKLEFGNYPKKEVDASSRTLKPWEGRIYISE    589
                     .:|.:.:|.....|||  .:.|.|||.|||||||||:.:
UNIPROT: P53341  548 SLPREGASLSFILGNY--DDTDVSSRVLKPWEGRIYLVK    584


#---------------------------------------
#---------------------------------------
```
